# Chemotaxonomic Markers for the Leaf Buds of Common Finnish Trees and Shrubs: A Rapid UHPLC MS Fingerprinting Tool for Species Identification

**DOI:** 10.3390/molecules27206810

**Published:** 2022-10-11

**Authors:** Marianna Manninen, Maarit Karonen, Juha-Pekka Salminen

**Affiliations:** Natural Chemistry Research Group, Department of Chemistry, University of Turku, FI-20014 Turku, Finland

**Keywords:** chemotaxonomy, fingerprint, liquid chromatography, mass spectrometry

## Abstract

In this study, a chemotaxonomic tool was created on the basis of ultra-high-performance liquid chromatography–mass spectrometry (UHPLC–MS) for the identification of 13 common Finnish deciduous trees and shrubs from their leaf bud metabolites. The bud extracts were screened with UHPLC–ESI–QqQ–MS and UHPLC–ESI–Q–Orbitrap–MS to discover suitable markers for each species. Two approaches were tested in the marker selection: (1) unique species-specific markers to obtain selective fingerprints per species and (2) major markers to maximise the sensitivity of the fingerprints. The markers were used to create two selected ion-recording-based fingerprinting tools with UHPLC–ESI–QqQ–MS. The methods were evaluated for their selectivity, repeatability, and robustness in plant species identification by analysing leaf buds from several replicates of each species. The created chemotaxonomic tools were shown to provide unique chromatographic profiles for the studied species in less than 6 min. A variety of plant metabolites, such as flavonoids, triterpenoids, and hydroxycinnamic acid derivatives, were found to serve as good chemotaxonomic markers for the studied species. In 10 out of 13 cases, species-specific markers were superior in creating selective and repeatable fingerprints.

## 1. Introduction

The chemical diversity of plants and the special chemical features of different plant families, genera, and species provide exciting possibilities for their discrimination and chemotaxonomic classification. Such discrimination is useful for not only educational [1] and species identification purposes [2] but also quality control [3,4,5] as different types of plant-based natural products increase in their popularity. Depending on the need, know-how, and available equipment, different types and levels of tools are needed to execute these discriminative or chemotaxonomic actions. 

The development of modern liquid chromatographic (LC), mass spectrometric (MS), and LC–MS fingerprinting tools for plant extracts offer a vast amount of information that can be used in multiple ways to reveal species similarities and differences. For instance, HPLC–UV was used by Lahtinen et al. [2] to distinguish *Betula pubescens-* and *Betula pendula*-type birch species, and HPTLC (high-performance thin-layer chromatography) was used by Melnyk et al. [3] for the identification of lime flowers. Direct Analysis in Real Time (DART) mass spectrometry has been shown to be a fast and reproducible tool in distinguishing *Datura* species from one another via their seeds and in differentiating several *Salvia* species from each other [6,7]. Furthermore, the LC–MS data of 74 medicinal plant extracts and machine learning were employed by Kharyuk et al. [8] for the identification of plant species and plant organs, with over 90% accuracy in classification. 

For common Finnish deciduous trees, there is a wide range of structurally different compounds described in the scientific literature that could serve as chemotaxonomic markers. *Populus tremula* is a good example, with phenolic acid glycerols characteristic for its leaf buds [5], whereas anthocyanins have been suggested as chemotaxonomic markers for the anthers of different *Populus* species [9]. In addition, Abreu et al. and Julkunen-Tiitto found that salicylate-like simple phenolic glucosides could serve as chemotaxonomic markers at the genus level for both *Populus* and *Salix* species [10,11]. Species-specific, genus-specific, and family-specific chemotaxonomic markers have also been identified for other common Finnish deciduous trees. Flavonoids have been shown to be a useful fool for distinguishing *B. pubescens-* and *B. pendula*-type birch species [2], as well as for characterising the flowers of different *Tilia* species [3]. Diarylheptanoids are characteristic of the genus *Alnus*, and diarylheptanoids of bark extracts of *Alnus incana* and *Alnus glutinosa* have been shown to be reliable indicators for identification and discrimination between the species [12]. Triterpenoids have potential to serve as chemotaxonomic markers in the genus of *Sorbus*, since some *Sorbus* species have provided structurally novel compounds and a substantial part of crude plant material (especially of fruits) is constituted by triterpenoids [13]. Almost all the genera and species of the family of Oleaceae contain iridoids, and the occurrence of iridoids from the different biosynthetic pathways correlate well with phylogenetic classification [14]. 

The identification of a tree species from a leaf bud is not a trivial task, but LC–MS/MS could be a useful tool for this identification task. However, only one study describing an LC–MS-based method for the identification of *B. pubescens* and *B. pendula* from leaf buds has been published thus far [1]. The primary aim of the current study was to compare two different approaches for the selection of the most suitable chemotaxonomic markers for the leaf buds of 13 common Finnish deciduous trees. Another aim was to utilise these markers in a simple, rapid, and repeatable LC-ESI-QqQ tool that produces such species-specific fingerprints that are easy to interpret.

## 2. Results and Discussion

### 2.1. Two Approaches in the Development of the LC–MS Fingerprinting Method and Selection of Marker Candidates

**Figure 1:**
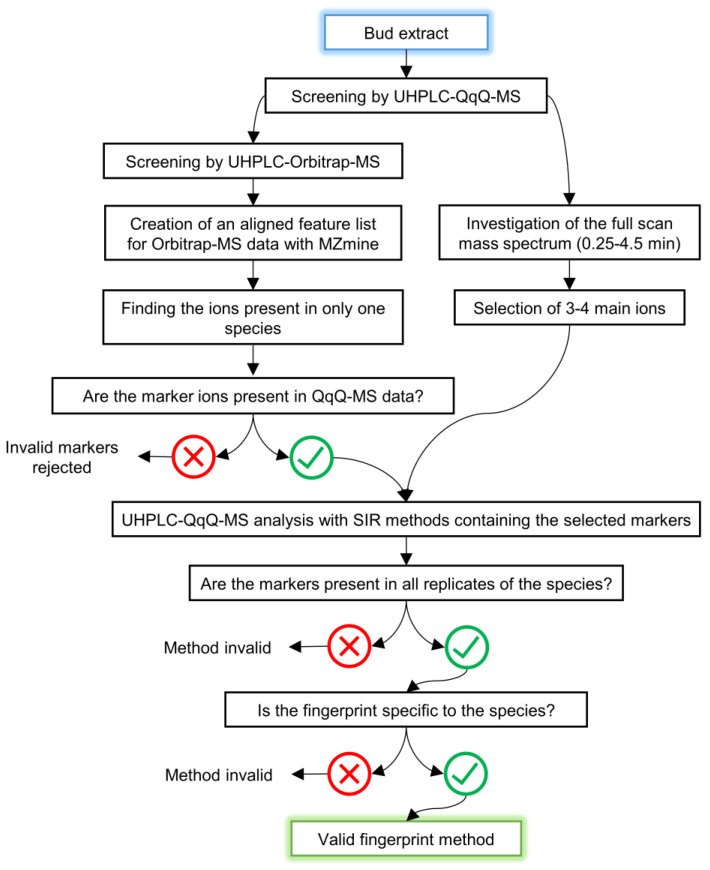
Flow chart of the method development of two different methods.

We used different steps in the method development, which are visualised as a flow chart in Figure 1. The leaf bud extracts were screened with two UHPLC–MS instruments to detect all potential marker candidates for each species. Two approaches were applied for the marker selection: one for the most species-specific markers and one for the most species-sensitive markers (described in more detail in Section 2.1.1. and Section 2.1.2.). The selected markers were used to create selected ion recording (SIR) methods for species fingerprinting with UHPLC–QqQ–MS. The individual SIR methods of a species were grouped together to enable the rapid acquisition of species-specific sum traces of all the selected SIR chromatograms. The repeatability and specificity of the fingerprints were estimated by analysing replicate leaf buds from 4–10 plant individuals per species and qualitatively comparing the acquired fingerprints.

#### 2.1.1. Method I: Species-Specific Markers from the High-Resolution MS Data Obtained with MZmine 2

**Figure 2:**
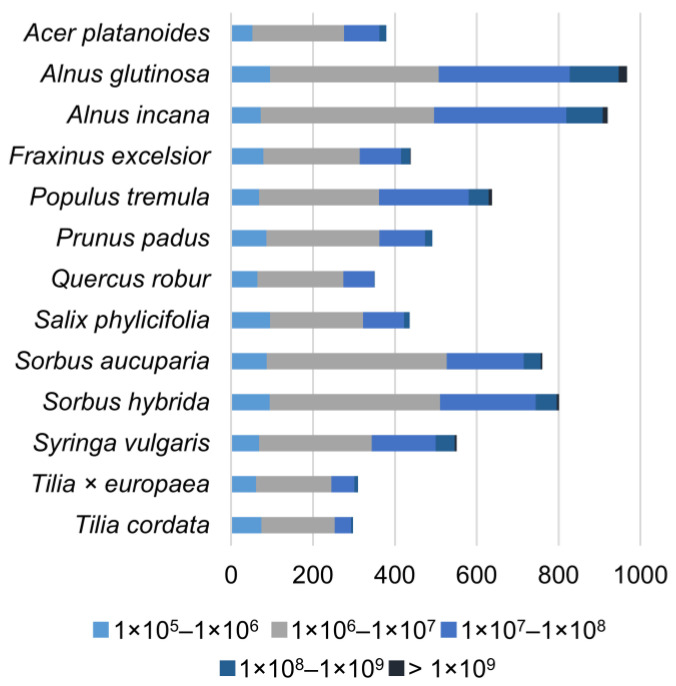
The number of features detected with MZmine 2 in the UHPLC-Orbitrap-MS data of the studied species. On the basis of the peak areas, the features were categorised into six categories that are denoted with different colours.

High-resolution MS data were obtained for one replicate of each species and transferred for further processing into MZmine 2, which is an open-source software for mass spectrometric data processing. It was used to create extracted ion chromatograms (EICs) for all ions with an intensity above 1×10^5^ and to align all features (i.e., detected variables with a retention time and an *m/z* ratio) into a feature list. The feature list enabled the comparison of the presence of detected ions in different leaf bud extracts. The data also revealed differences in the chemical diversity of the leaf bud extracts. The results showed that the chemical diversity between species from the same plant genus was similar. Both *Alnus* species produced a high number of detected features, making them the most chemically diverse species among the studied species (Figure 2). The smallest number of detected features was obtained for the *Tilia* species. In addition to the *Alnus* and *Tilia* species, the numbers of detected features from both *Sorbus* species were similar. Additionally, these species were similar in terms of the number of features in different categories according to the peak areas.

The marker candidates were chosen from the aligned feature list by excluding features that could be detected in multiple species. For each species, between three and seven marker candidates with the highest peak area were chosen for further testing. Since the goal was to create a simple fingerprinting method for QqQ–MS, the presence of the candidates in the QqQ–MS data was confirmed from the full scan MS data. To maximise the ion intensity of the chosen markers, a preliminary SIR test was performed with different cone voltages using four replicate leaf buds of the same plant individual. The cone voltage producing the highest ion intensity was used in the method for the testing of repeatability and specificity. 

With this approach, the markers specific to *S. aucuparia* and present in the QqQ–MS data could not be found, but common markers to both *S. hybrida* and *S. aucuparia* were found. However, markers specific to *S. hybrida* were discovered, so two SIR methods were created for *Sorbus* species. The first method was able to distinguish *Sorbus* species from other species, while the second method was able to identify *S. hybrida* species, thereby enabling us to separate *S. hybrida* and *S. aucuparia* samples from each other.

#### 2.1.2. Method II: Main Ions from the QqQ Full Scan Spectra

As a comparison to the previously described approach, a more straightforward approach for choosing marker candidates was applied. The full scan MS screening was conducted for all replicates of the studied species. The results showed that the full scan spectra were repeatable within species (data not shown). For each species, from three to six main ions from the full scan spectrum were chosen as the marker candidates. We omitted the time range of 0.00–0.25 min from the full scan spectrum to rule out the most polar and early eluting compounds such as sugars and other primary metabolites that are likely to be present in many species. This also enhanced the uniqueness and species-specificity of the fingerprints since most of the species would have otherwise had SIR detection at the same retention time window. 

The full scan spectra of both *Sorbus* species were similar in terms of the main ions (Supplementary Materials, Figures S9 and S10). However, the intensity ratios of the main ions were different between the species, indicating that the combined SIR traces would have different profiles. Thus, only one joint method was created for these species. Neither of the two approaches used were able to find markers to reliably differentiate *Tilia cordata* and *Tilia × europaea*. The same main ions were present in both species (Supplementary Materials, Figures S12 and S13), and the marker candidates from the MZmine data could not be reliably detected from the QqQ–MS data. Instead, marker candidates for the differentiation of *Tilia* species from other species were discovered with both approaches. Consequently, only two methods that were specific to both *Tilia* species were created, one using the marker candidates from MZmine and the other one using the main ions as markers.

#### 2.1.3. Repeatability of the Fingerprints

Fingerprint repeatability was evaluated by comparing the 4–10 replicate fingerprint profiles within species. The fingerprints were considered repeatable if the main peaks were the same in all replicates and no additional peaks were detected. A slight difference in the intensities of the main peaks compared to each other was considered acceptable. The fingerprints of all replicates of all species with the species-specific methods I and II can be found in the Supplementary Information.

**Figure 3:**
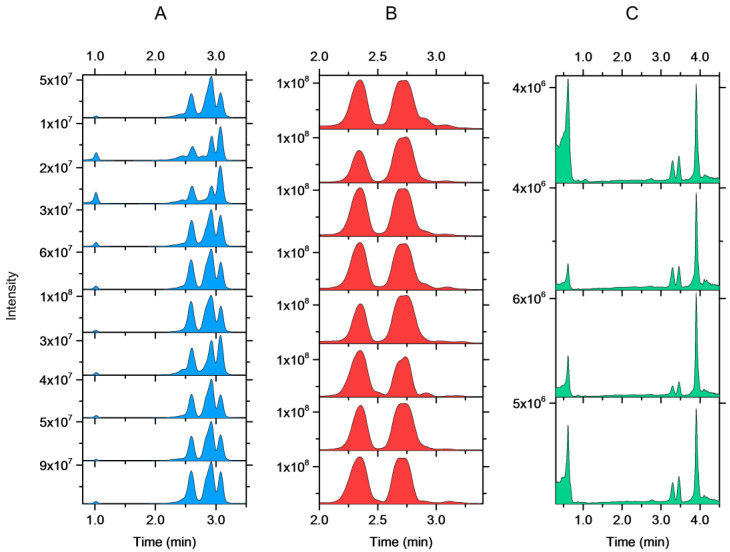
Within-species repeatability of the fingerprints in the different plant individuals of (**A**) *Sorbus hybrida*, (**B**) *Alnus glutinosa,* and (**C***) Salix phylicifolia* recorded with the species-specific method II.

As was expected on the basis of the good repeatability of the full scan mass spectra, good repeatability was obtained for all species with method II. For example, all replicates of *S. hybrida, A. glutinosa*, and *S. phylicifolia* produced the same main peaks with the fingerprinting methods using the main ions (Figure 3). With method I, fingerprints of 10 species were repeatable. Some challenges were detected with the remaining three species (*S. hybrida, A. glutinosa,* and *S. phylicifolia*). However, *A. glutinosa* and *S. phylicifolia* could still be correctly identified due to other peaks in the fingerprint.

**Figure 4:**
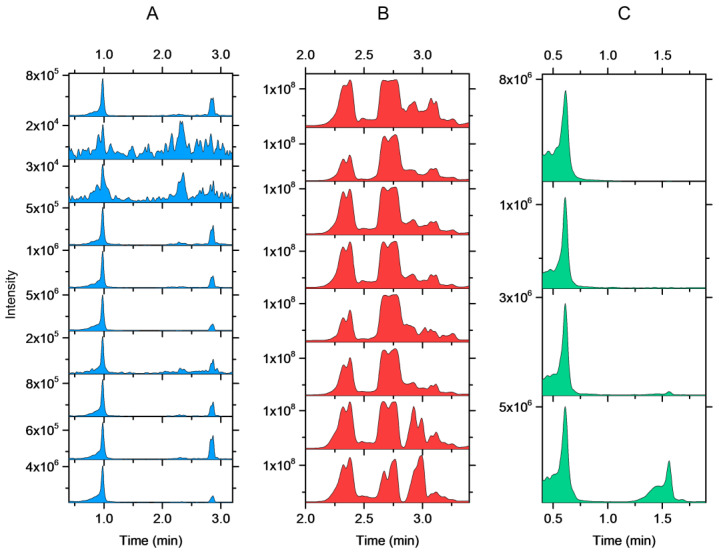
Within-species repeatability of the fingerprints in the different plant individuals of (**A**) *Sorbus hybrida*, (**B**) *Alnus glutinosa,* and (**C**) *Salix phylicifolia* recorded with the species-specific method I. The results were obtained for the same samples as in Figure 3.

Method I for separating *Sorbus* species from other species provided similar results for all *S. aucuparia* and *S. hybrida* samples. However, the method for *S. hybrida* provided the expected result for 8 out of 10 *S. hybrida* samples (Figure 4A). The fingerprints of the other two *S. hybrida* samples were noisy and lacked the characteristic peaks. Thus, the reliable identification of *S. hybrida* was not possible. An additional three leaf buds of both of these exceptional plant individuals were analysed, and two out of three replicates produced the correct fingerprint with the *S. hybrida* method. The repeatability issue could therefore be related to the low concentration of the marker compounds in the leaf buds.

The fingerprints of all replicates of *A. glutinosa* obtained with method I exhibited the same two main peaks. However, the fingerprints of two replicates showed additional peaks at a later retention time (Figure 4B). Therefore, the method was not 100% reliable. Identification was still possible, as the other species did not produce a fingerprint that could have been misinterpreted as *A. glutinosa*. Similarly, the fingerprints of all *S. phylicifolia* samples had the same main peak, which was not present in the fingerprints of any other species. The fingerprints of two replicates had an additional peak at 1.57 min, which was not observed in all the replicates (Figure 4C).

#### 2.1.4. Specificity of the Fingerprints

The specificity of the fingerprints was evaluated by comparing the fingerprint of a selected species to the fingerprints of other species with the same species-specific method. The specificity of the fingerprints was studied from two perspectives: (1) is the intensity of the peaks in the fingerprint of the studied species significantly higher compared to the other species and (2) does the profile of the fingerprint clearly differ from the fingerprint profiles of other samples with the same method? Good specificity regarding intensity would enhance the readability of the results, as scaling the y-axes of all samples according to the most intensive peak of the studied species would lead to the species in question standing out from other species. Good specificity regarding the fingerprint profile meant that the fingerprint profile of the species in question was unique, which made it easier to compare the fingerprints of different species. 

**Figure 5:**
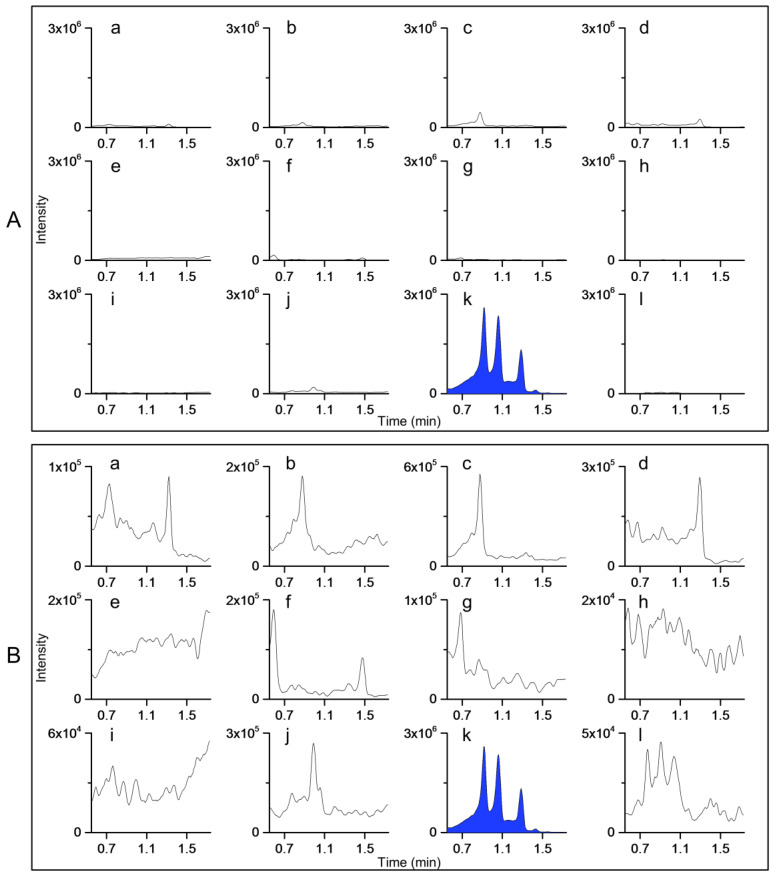
Fingerprints of all studied species with the species-specific method I for *Syringa vulgaris*. (**A**) The y-axes of all samples were scaled according to the most intensive peak of all samples, which was produced by *S. vulgaris*. (**B**) The y-axes were scaled to the most intensive peak of each sample. Plant species are as follows: (**a**) *Acer platanoides*, (**b**) *Alnus glutinosa*, (**c**) *Alnus incana*, (**d**) *Fraxinus excelsior*, (**e**) *Populus tremula*, (**f**) *Prunus padus*, (**g**) *Quercus robur*, (**h**) *Salix phylicifolia*, (**i**) *Sorbus aucuparia*, (**j**) *Sorbus hybrida*, (**k**) *Syringa vulgaris*, and (**l**) *Tilia cordata*. *Tilia × europaea* produced a similar fingerprint to that of *Tilia cordata*.

**Figure 6:**
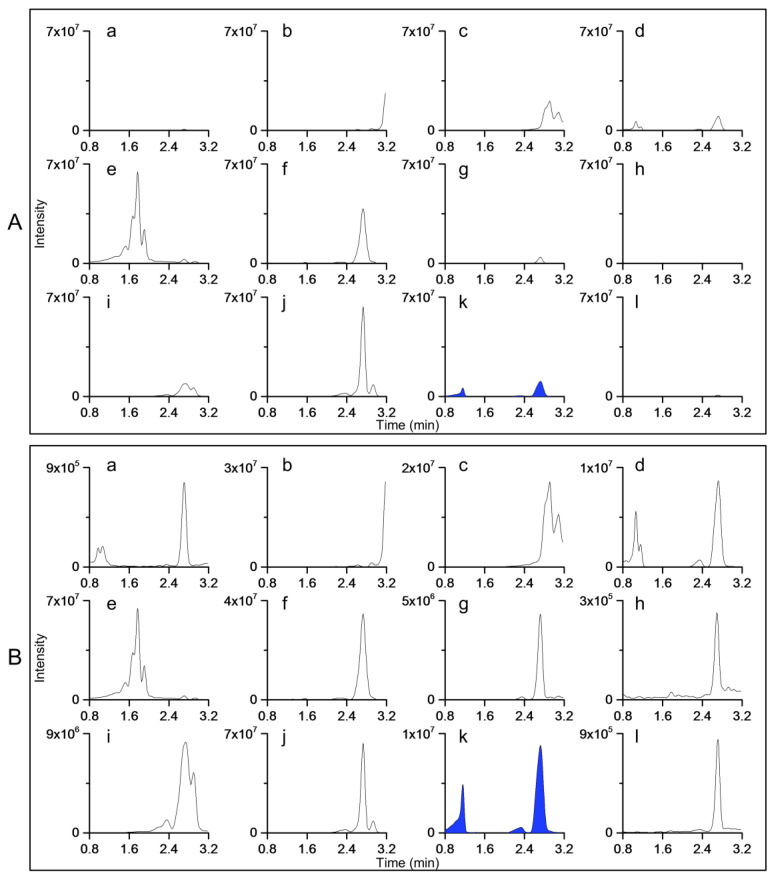
Fingerprints of all studied species with the species-specific method II for *Syringa vulgaris*. (**A**) The y-axes all samples were scaled according to the most intensive peak of all samples, which was produced by *Populus tremula* (e). (**B**) The y-axes were scaled to the most intensive peak of each sample. Plant species are as follows: (**a**) *Acer platanoides*, (**b**) *Alnus glutinosa*, (**c**) *Alnus incana*, (**d**) *Fraxinus excelsior*, (**e**) *Populus tremula*, (**f**) *Prunus padus*, (**g**) *Quercus robur*, (**h**) *Salix phylicifolia*, (**i**) *Sorbus aucuparia*, (**j**) *Sorbus hybrida*, (**k**) *Syringa vulgaris*, and (**l**) *Tilia cordata*. *Tilia × europaea* produced a similar fingerprint to that of *Tilia cordata*.

For example, method I for *S. vulgaris* was more specific regarding intensity, as the traces of other species dropped to baseline when the y-axes were scaled according to the most intensive peak of *S. vulgaris* (Figure 5A). Method I for *S. vulgaris* was also more specific regarding the fingerprint profile. *S. vulgaris* was the only species that produced three clear peaks, while the fingerprint profiles of other species were noisier and exhibited none of the main peaks of the fingerprint of *S. vulgaris* (Figure 5B). With method II for *S. vulgaris*, the fingerprints of other species were more dominant (Figure 6A). Furthermore, the profile-based specificity was poorer compared with that of method I, as most of the fingerprints of other species had the same peak at 2.73 min, which was also present in the fingerprint of *S. vulgaris* (Figure 6B).

### 2.2. Comparison between Different Fingerprinting Methods

Information on the repeatability and specificity of different fingerprinting methods is summarised in Table 1. For most of the species, both methods were valid. For some species, even the same ions were chosen as markers with both methods. The better specificity of method I resulted in its selection as the final method for most species. However, method II produced clearer and more repeatable results for both *Sorbus* species, and for that reason, method II was chosen as the final method for *Sorbus* species. Similarly, method II was chosen as the final approach for *A. glutinosa* due to its better repeatability.

**Figure 7:**
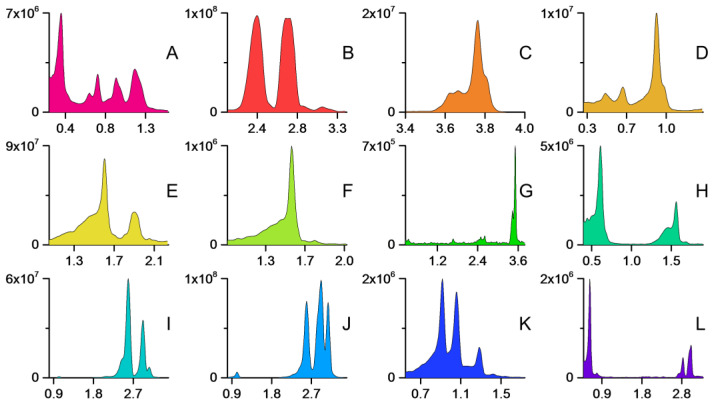
The LC–MS fingerprints of the studied species with the species-specific methods. (**A**) *Acer platanoides*, (**B**) *Alnus glutinosa*, (**C**) *Alnus incana*, (**D**) *Fraxinus excelsior*, (**E**) *Populus tremula*, (**F**) *Prunus padus*, (**G**) *Quercus robur*, (**H**) *Salix phylicifolia*, (**I**) *Sorbus aucuparia*, (**J**) *Sorbus hybrida*, (**K**) *Syringa vulgaris*, and (**L**) *Tilia cordata*. *Tilia × europaea* produced a similar fingerprint to that of *Tilia cordata*. The LC–MS fingerprints consisted of selected ion recording traces of 1–6 markers (Table 2).

The final fingerprinting method resulted in unique chromatographic profiles for each plant species with the species-specific SIR method (Figure 7). The results reinforced the observation of the chemical similarity of the two *Sorbus* and *Tilia* species, which was also noted from the number of features in the MZmine data. In contrast, no difficulties were encountered in finding markers to *Alnus* species, indicating that, even though the chemical diversity of both species was significant, qualitative differences could easily be found.

### 2.3. Identification of the Markers Based on the UHPLC–DAD–QOrbitrap–MS/MS Data

Among the studied species, the Orbitrap data of the markers led to the identification of 42 individual compounds that were analysed in detail. The characterisation was based on the comparison of UV spectra, mass spectra, and MS/MS fragmentation with published data. The data revealed a diverse range of phenolic compounds and triterpenoids. Table 2 summarises the UHPLC–DAD–QOrbitrap-MS/MS data of the markers including UV maxima, retention times, exact masses, and main fragment ions. However, these data could not reveal the exact positions for the substituents and functional groups nor the stereochemistry, so other isomers are possible. The Supplementary Information includes the MS/MS spectra for all markers and molecular formulas for the main fragment ions, along with the proposed fragmentation patterns for the suggested compounds.

#### 2.3.1. Flavonoids

Some of the markers were identified as flavonoids. The main peak in the LC–MS fingerprints of *S. phylicifolia* samples was marker **3** at *m/z* 319. It was identified as dihydromyricetin, which has been previously reported in *S. phylicifolia* [15,16]. The fragments in the MS/MS spectrum at *m*/z 125 and 193, resulting from the loss of the B ring, were consistent with those reported for dihydromyricetin [17]. 

A similar UV spectrum to that of dihydromyricetin was obtained for marker **4**, which was the main peak in the LC–MS fingerprints of *Tilia* samples. The exact mass matched dihydroquercetin glucoside, the main fragments at *m/z* 285 could correspond to the loss of glucose and water, and the fragment at *m/z* 151 could correspond to the loss of the A ring, all of which have been previously reported for dihydroquercetin derivatives [18,19]. The flowers of different *Tilia* species have been shown to contain different flavonoids such as flavonols and flavanones [20], but dihydroflavonols have not been reported in *Tilia* species. 

Another proposed flavonoid derivative is marker **21**, which was a minor peak in the fingerprints of *P. padus*. The MS/MS spectrum indicated a flavanone aglycone naringenin with the [A–H]^−^ ion at *m/z* 271. The other fragments at *m/z* 119 and 151 could be attributed to the loss of the B and C rings and the loss of the A ring, respectively [21]. Naringenin and its derivatives have been previously found in different plant parts of the *Prunus* species [22,23].

#### 2.3.2. Hydroxycoumarins

Marker **5** was a minor peak in the fingerprints of *F. excelsior* samples. The MS/MS spectrum showed fragments at *m/z* 163, 191, 206, and 221, which were consistent with those reported for fraxidin derivatives [24], but no further conclusions could be drawn about the side group. Fraxidin derivatives were previously found in *F. excelsior* [25].

#### 2.3.3. Glycerol Esters of Cinnamic Acid Derivatives

All four markers of *P. tremula* were found to be phenolic acid glycerols, and (except for 2-acetyl-1,3-di-feruloyl glycerol (**24**)) they have previously been reported in *P. tremula* leaf buds [5]. Marker **22** was identified as 2-acetyl-1,3-di-*p*-coumaroyl glycerol, and its MS/MS spectrum included fragment ions characteristic of coumaric acid at *m/z* 117, 119, 145, and 163. Marker **23** was 2-acetyl-3-*p*-coumaroyl-1-feruloylglycerol, and marker **24** was 2-acetyl-1,3-di-feruloylglycerol. Marker **23** shared the same fragments with marker **22**, both containing coumaroyl moieties. The additional fragments of marker **23** at *m/z* 134, 160, 175, and 193, were found in the MS/MS spectrum of marker **24**, which also contained feruloyl moieties. Marker **19** was identified as 2-acetyl-1,3-di-caffeoylglycerol, and it showed fragments characteristic of caffeic acid at *m/z* 133, 135, 161, and 179. The observed fragments and the UV spectra of the markers were also consistent with those reported in previous studies [5,26]. In the fingerprints of *P. tremula* samples, 2-acetyl-1,3-di-caffeoylglycerol was shown as a separate, more intensive peak, while the other three phenolic acid glycerols formed a less intensive hump at a later retention time.

#### 2.3.4. Other Cinnamic Acid Derivatives

Marker **13** was identified as monocaffeoylquinic acid, and it was a minor peak in the fingerprints of the *Sorbus* samples. Due to the coelution of other compounds, a reliable UV spectrum could not be obtained. However, the MS/MS spectrum showed typical fragments to caffeoylquinic acids: a [quinic acid–H]^−^ ion at *m/z* 191 and a [caffeic acid–H]^−^ ion at *m/z* 179, as well as its fragments at *m/z* 135 and 161. Monocaffeoylquinic acids have been reported in many species of the genus *Sorbus* [13]. 

Marker **18**, the main peak in the fingerprints of *P. padus* samples, could also include a coumaric acid moiety due to fragments at *m/z* 117, 145, and 163. The UV spectra with a maximum at 315 nm indicates the same. The molecular formula matched penta-*O*-acetyl-*p*-coumaroylsucrose, whose isomers have been identified in *Prunus mume* [27]. However, the identification is speculative since the fragments only evidence a coumaroyl moiety. 

The marker of *Q. robur* (**39**) was also tentatively identified as a caffeic acid derivative on the basis of the similarity of its fragments to those of other compounds identified with a caffeic acid moiety. The late retention time at 3.55 min suggests that the compound has a side chain that increases the retention.

#### 2.3.5. Salicylate-like Phenolic Glycosides

Marker **20** was identified as tremulacin, a known compound in *Salix* species [11,28,29]. The fragmentation matched with that reported by Kammerer et al. [28]. However, it was not present in all *S. phylicifolia* samples, and dihydromyricetin (**3**) was therefore the main marker for the species. Tremulacin has also been reported in *P. tremula* [10], and it was also observed in the *P. tremula* samples of this study, although at a lower intensity.

#### 2.3.6. Secoiridoids

All four markers of *S. vulgaris* were secoiridoids, commonly known markers in Oleaceae [14]. Two of the markers, demethyloleuropein (**10**) and demethylligstroside (**14**), are oleuropein-like secoiridoids, and they have been characterised in *S. vulgaris* flower and fruit extracts [30]. The other two markers were 2″-epi-frameroside (**16**) and hydroxyframoside (**17**). Additionally, both of these have been observed in different parts of *S. vulgaris* previously [31]. Demethyloleuropein, demethylligstroside, and 2″-epi-frameroside were the three main peaks observed in the fingerprints of the *S. vulgaris* samples.

#### 2.3.7. Phenylethanoids

Markers **9** and **12** were identified as calceolariosides A and B, respectively, which were shown in the *F. excelsior* LC–MS fingerprints as the main peak with a shoulder. In both cases, the MS/MS spectrum showed caffeic acid at *m/z* 179 and its fragments at *m/z* 133 and 161. Additionally, an [M–H–162]^−^ ion at *m/z* 315 was observed, indicating the loss of a caffeic acid moiety. Eyles et al. reported calceolariosides A and B in other *Fraxinus* species, and according to their data, calceolarioside A elutes before B in reverse-phase chromatography [24]. Therefore, markers **9** and **12** are proposed to be calceolarioside A and calceolarioside B, respectively.

#### 2.3.8. Benzoic Acid Glycosides

All four markers of *A. platanoides* were gallic acid and/or syringic acid glycosides. Glucosyringic acid (**1**) was observed as an [M–H]^−^ ion at *m/z* 359, and it has been found in *Acer saccharum* Marsh. buds previously [32]. The MS/MS spectrum showed an [M–H–162]^−^ or [syringic acid–H]^−^ ion at *m/z* 197, and an [M–H–44–30]^−^ ion at *m/z* 123 due to carboxyl and methoxyl losses. Markers **6**, **7**, and **8** shared some fragment ions with the glucosyringic acid. Additionally, the MS/MS spectrum showed an [M–H–198]^−^ ion at *m/z* 313 from the cleavage of syringic acid, an [M–H–152–14]^−^ ion at *m/z* 345, and a [gallic acid–H]^−^ ion at *m/z* 169. Therefore, the compounds were tentatively identified as monogalloyl syringyl glucoses. They eluted as three not fully separated peaks at 0.70, 0.79, and 0.88 min, suggesting three isomers. Marker **11** also showed evidence of galloyl and syringyl moieties. The MS/MS spectrum showed an [M–H–152]^−^ ion at *m/z* 511 from the loss of gallic acid, an [M–H–198]^−^ ion at *m/z* 465 from the loss of syringic acid, and an [M–H–152–198]^−^ ion at *m/z* 313 (loss of galloyl group and syringic acid). The compound was suggested to be digalloyl syringyl glucose. 

Marker **15** at *m/z* 621 was the [M–2H]^2–^ ion of heptagalloyl glucose previously identified in *A. platanoides* [33]. The MS spectrum also showed an [M–H]^−^ ion at *m/z* 1243. It eluted at 1.15 min and had UV maxima at 221 and 278 nm. The MS/MS spectrum showed an [M–H–152–152–170]^−^ ion at *m/z* 769, an [M–2H–152–152]^2–^ ion at *m/z* 469, an [M–H–152–152–152–170]^−^ ion at *m/z* 617, an [M–2H–152–152–152]^2–^ ion at *m/z* 393, and a [gallic acid–H]^−^ ion at *m/z* 169. 

All four markers contributed to the LC–MS fingerprints of *A. platanoides* samples. Glucosyringic acid was the most intensive peak at the earliest retention time. The second peak with a shoulder was monogalloyl syringyl glucose, the third peak was digalloyl syringyl glucose, and the fourth peak was heptagalloyl glucose.

#### 2.3.9. Triterpenoids and Triterpenoid Derivatives

Several markers of *Sorbus* samples were triterpenoids or triterpenoid derivatives. The UV and MS/MS spectra of markers **27** and **29** indicated that both of them included an additional hydroxycinnamic acid moiety. Marker **27** had a UV maximum at 309 nm and fragment ions characteristic of coumaric acid. The additional fragment ions of [M–H–18]^−^ at *m/z* 615 resulting from the cleavage of water, [M–H–44] ^−^ at *m/z* 589 resulting from the loss of a carboxyl group, and [M–H–18–44]^−^ at *m/z* 571 resulting from the loss of both could correspond for the triterpenoid part, which was tentatively identified as rotundic acid. De Tommasi et al. found similar triterpenoid derivatives in *Eriobotrya japonica*, including 3-*O*-*trans*-*p*-coumaroylrotundic acid [34]. Marker **29** had the same molecular formula but different UV and MS/MS spectra. The UV maximum was at 322 nm, and the fragment ions were characteristic of caffeic acid. The molecular formula matched that of 2-*O*-caffeoylmaslinic acid, as reported by Yang et al. in *Hippophae rhamnoides* [35]. Marker **27** was shown in the fingerprints of both *Sorbus* samples as a small shoulder before the main peak at 2.59 min. Marker **29** did not contribute to the overall fingerprint, as marker **30** eluted at the same retention time and produced a more intensive peak. 

Marker **30** exhibited the first intensive peak in the fingerprints of both *Sorbus* species. It was tentatively identified as cashmirol B. The loss of carboxylic acid could be observed from the MS/MS spectrum by an ion at *m/z* 423 with a low intensity. Cashmirol B has been previously reported in *Sorbus cashmiriana* [36]. 

Markers **35**, **36**, and **38** were identified as either oleanane- or ursane-type triterpenoids, both of which are common in *Sorbus* species [13]. It has been shown that these types of pentacyclic triterpenes produce few fragment ions when negative ionisation is used [37]. However, a minor fragment ion at *m/z* 451 was observed for marker **35**, which could have been due to the cleavage of the acetyl group. On the basis of the molecular formula, the compound could be oxoursolic acid acetate or oxooleanolic acid acetate. No fragments could be obtained for marker **36**. The exact mass matched with oleanonic acid or ursonic acid. The fragment ion at *m/z* 437 in the MS/MS spectrum of marker **38** also indicated the cleavage of an acetyl group, and the compound could therefore be tentatively assigned as acetyl ursolic acid or acetyl oleanolic acid. Marker **36** exhibited one of the main peaks in the fingerprints of both *Sorbus* species. Marker **38** was the third main peak at 3.06 min in the fingerprints of *S. hybrida* samples, but it was only detected in the *S. aucuparia* samples as a small shoulder. Marker **35** was observed in the fingerprints of *Sorbus hybrida* as a small shoulder at 2.82 min, but it did not contribute to the overall fingerprint of the *S. aucuparia* samples, even though it could be observed in the EIC. 

In addition to the *Sorbus* species, the markers of *A. glutinosa* were identified as triterpenoid derivatives. In the fingerprints of the *A. glutinosa* samples, markers **31** and **33** exhibited one intensive peak without a proper separation between the two isomers. They were tentatively identified as the isomers of curculigosaponin B. The MS spectrum showed both an [M–H]^−^ ion at *m/z* 605.4 and an [2M–H]^−^ ion at *m/z* 1211.8. The MS/MS spectrum showed an [M–H–132]^−^ ion at *m/z* 473 resulting from the cleavage of arabinose and a minor [M–H–132–18]^−^ ion at *m/z* 455 resulting from the additional cleavage of water. Although curculigosaponin B has only been previously reported in *Curculigo orchioides* [38], other tetracyclic triterpenes have been reported in several *Alnus* species [39]. Markers **32** and **34** did not contribute to the overall fingerprints of the *A. glutinosa* samples because of the higher intensity of the other markers. They were also identified as saponins, isomers, of alnustic acid arabinoside. The MS/MS spectrum showed aglycone at *m/z* 487. The loss of the carbon side chain yielding a fragment ion at *m/z* 389 and the following cleavage of carboxyl group or water resulting in the ions at *m/z* 345 and 371, respectively, were also detected in the MS/MS spectrum. Alnustic acid arabinoside was initially characterised in *Alnus serrulatoides* [40], and later discovered in some other *Alnus* species as well [41]. 

Compared with the other markers of *A. glutinosa*, the MS/MS spectrum of markers **25** and **26** had similar characteristics. The lack of a UV spectrum and similar retention times suggested that markers **25** and **26** could be structurally similar to markers **31**, **32**, **33**, and **34**, meaning that they could also be triterpenoid saponins, although with a different sugar part because the neutral loss of glucose or arabinose could not be detected from the MS/MS spectrum. Markers **25** and **26** produced the first main peak in the fingerprints of the *A. glutinosa* samples.

#### 2.3.10. Other Compounds

Markers **2**, **28**, **37**, and **40**–**42** could not be reliably assigned to any of the compound classes discussed above. The molecular ion of marker **2** had an even *m/z* value, which suggests that it contains and odd number of nitrogen atoms. Marker **2** was a minor peak in the fingerprints of *F. excelsior*. Marker **28** was detected in all of the studied species. However, the combination of **28** and **37** detected as two peaks was not obtained for any other species than the *Tilia* species. Markers **40**–**42**, found in *A. incana*, shared similar MS/MS characteristics, which could indicate that they belong to the same molecular family. The smallest fragments could have been derived from a coumaroyl moiety.

## 3. Materials and Methods

### 3.1. Chemicals

LC–MS-grade acetonitrile was purchased from Merck (KGaA, Darmstadt, Germany), and LC–MS-grade formic acid was purchased from VWR International (Fontenay-Sous-Bois, Paris, France). Water was purified with the Millipore Synergy water purification system (Merck KGaA, Darmstadt, Germany). Ethanol (99.5%, Aa grade) was purchased from Altia (Rajamäki, Finland).

### 3.2. Plant Samples

Leaf bud samples were collected from the Turku area, Southwest Finland, in spring of 2019, 2020, and 2021. After collection, the samples were frozen, lyophilised, and stored in a freezer. The extraction protocol was modified from Lahtinen et al. [2]. The extracts were prepared by dropping one leaf bud into 2 mL of ethanol–water (95/5, *v*/*v*) solution and shaking for 10 min. The extract was filtered with a 0.20 µm PTFE filter.

### 3.3. UHPLC–QqQ Full Scan Screening and Fingerprinting Analysis

The screening for markers and final fingerprinting analysis was performed using an Acquity UPLC system (Waters Corporation, Milford, MA, USA) coupled with a Xevo TQ triple−quadrupole mass spectrometer (Waters Corp.). The UPLC system consisted of a sample manager, a binary solvent manager, a column (Acquity UPLC BEH Phenyl 30 mm × 2.1 mm, 1.7 μm, Waters Corporation, Ireland), and a diode array detector. The mobile phase consisted of acetonitrile (A) and water/formic acid (99.9:0.1, *v/v*) (B). The elution profile was as follows: 0–0.3 min, 10% A in B; 0.3–3.1 min, 10–75% A in B (linear gradient); 3.1–3.5 min, 75% A in B; 3.5–3.6 min, 75–95% A in B; and 3.6–5.0 min column wash and stabilisation. The flow rate was 0.65 mL/min, and the injection volume was 5 μL. Mass analyses were performed using an ESI source and negative ionisation. The ESI conditions were as follows: capillary voltage, 1.8 kV; source temperature, 150 °C; desolvation temperature, 650 °C; desolvation and cone gas (N_2_), 1000 and 100 l/h, respectively; and collision gas, argon. The mass range for the full scan screening was set to *m/z* 150–2000. The selected ion recording (SIR) parameters are presented in Table 3.

### 3.4. UHPLC–QOrbitrap–MS/MS Analysis

A similar Acquity UPLC system was configured with a hybrid quadrupole–Orbitrap mass spectrometer (QExactive, Thermo Fisher Scientific GmbH, Bremen, Germany). The column and gradient were similar to the ones used in the QqQ analysis. The injection volume was 5 μL, and the flow rate was 0.65 mL/min. The heated ESI source (H-ESI II, Thermo Fisher Scientific GmbH, Bremen, Germany) was operated in the negative ion mode. The parameters were set at as follows: spray voltage, −3.0 kV; sheath gas (N_2_) flow rate, 60 (arbitrary units); aux gas (N_2_) flow rate, 20 (arbitrary units); sweep gas flow rate, 0 (arbitrary units); and capillary temperature, +380 °C. The in-source collision-induced dissociation energy was 30 eV. A resolution of 35,000 and an automatic gain control of 3×10^6^ were used for full scan MS data. The mass range was set to *m/z* 150−2250. MS/MS data, namely, dd-MS2 (Top N) data, were acquired using a resolution of 17,500; an automatic gain control of 1×10^5^; a TopN of 7; and stepped normalised collision energies (NCEs) of 30, 50, and 80. The calibration was performed with a Pierce ESI Negative Ion Calibration Solution (Thermo Fisher Scientific Inc., Waltham, MA, USA) and was the most accurate at *m/z* > 250. The data were processed with Thermo Xcalibur Qual Browser software (Version 4.1.31.9, Thermo Fisher Scientific Inc., Waltham, MA, USA).

### 3.5. Data Analysis by MZmine 2

The MS/MS data files were converted from the Thermo Scientific .raw data format to .mzXML with the MS Convert Software included in the ProteoWizard package [42]. The .mzXML files were submitted to the MZmine 2.53 [43]. The mass detection was performed with noise levels of 1×10^5^ (for MS scans) and 1×10^4^ (for MS/MS scans). MS chromatograms were built using the ADAP chromatogram builder [44], with a minimum group scan size of 5, a group intensity threshold of 1×10^4^, a minimum highest intensity of 3×10^5^, and an *m/z* tolerance of 0.01. The chromatographic deconvolution was achieved by the local minimum search algorithm with the following settings: *m/z* range for MS/MS scan pairing of 0.01, RT range for MS/MS scan pairing of 0.1 min, chromatographic threshold of 75%, search minimum in RT range of 0.05 min, minimum relative height of 1%, minimum absolute height of 1×10^5^, minimum ratio of peak top/edge of 1.5, and peak duration range of 0.2–1.0 min. Chromatograms were deisotoped using the isotopic peaks grouper algorithm with an *m/z* tolerance of 0.01 and an RT tolerance of 0.01 min. The chromatograms were then aligned together into a feature list with the join aligner module at an *m/z* tolerance of 0.01 (weight for *m/z* = 75; weight for RT = 25; absolute RT tolerance = 0.1 min). The aligned feature list was gap-filled using the multithreaded peak finder module (intensity tolerance of 10%, *m/z* tolerance of 0.001, retention time tolerance of 0.05 min) and filtered using the peak filter module by area (5 × 10^5^–1 × 10^10^) and height (1×10^5^–1×10^10^). Finally, the feature list was filtered using the feature list rows filter to remove peaks with fewer than two peaks in an isotope pattern.

## 4. Conclusions

In this study, two alternative approaches were applied in the selection of marker candidates for the leaf buds of 13 common Finnish tree species. The approach using MZmine was found to be suitable for discovering species-specific markers, and it demonstrated differences in the chemical diversity of the studied species. The main ions chosen by the second approach were less specific to the species, but they produced repeatable fingerprints. The final UHPLC–MS fingerprinting tool utilised a combination of markers obtained with the different approaches. The fingerprinting tool enabled species identification from a single leaf bud in less than 6 min. The high-resolution mass data revealed that the markers belonged to many different plant metabolite subclasses such as flavonoids, hydroxycinnamic acid derivatives, and triterpenoids. The structural diversity of the markers qualitatively demonstrated the diversity in the leaf bud chemistry of different species.

## Figures and Tables

**Table 1:** Comparison of two fingerprinting methods. Repeatability is denoted as the number of replicates that produced similar fingerprints for all replicates. Intensity-based specificity was ranked as good if the species in question produced the most intensive signal, OK if one other species produced as intensive a peak as the species in question, and poor if the fingerprint of some other species was the most intensive. Profile-based specificity was ranked as good if the fingerprint profile of the species in question was unique and poor if the fingerprint profile of the species in question showed the same peaks as other species.

	Method I (Species-Specific Ions)			Method II (Main Ions)			
Species	Repeatability	Specificity		Repeatability	Specificity		Final Method
		Intensity-Based	Profile-Based		Intensity-Based	Profile-Based	
*Acer platanoides*	8/8	OK	good	8/8	poor	poor	I
*Alnus glutinosa*	6/8 *	good	good	8/8	good	good	II
*Alnus incana*	8/8	good	good	8/8	poor	poor	I
*Fraxinus excelsior*	9/9	good	good	9/9	poor	poor	I
*Populus tremula*	10/10	good	good	10/10	good	good	I
*Prunus padus*	5/5	good	good	5/5	poor	poor	I
*Quercus robur*	9/9	OK	good	9/9	poor	poor	I
*Salix phylicifolia*	3/4 *	good	good	4/4	poor	poor	I
Sorbus aucuparia †	9/9	poor	good	9/9	good	good	II
*Sorbus hybrida*	8/10	good	good	10/10	good	good	II
*Syringa vulgaris*	5/5	good	good	5/5	poor	poor	I
*Tilia cordata* and *Tilia × europaea*	15/15	poor	good	15/15	good	poor	I

* = There was some deviation in the fingerprint profiles, but the identification of species was still possible; † = Method I produced a similar fingerprint for both *S. aucuparia* and *S. hybrida.*

**Table 2:** Chromatographic, UV, and mass spectral data of the species-specific markers obtained from the UHPLC–DAD–ESI–QOrbitrap–MS.

No.	Species	UV Max (nm)	Retention Time (min)	Molecular Formula	[M−H]^−^	*m/z* Values of Main Fragment Ions	Exact Mass, Calculated	Exact Mass, Measured	Error (ppm)
1	*Acer platanoides*	212, 257, 300 (sh)	0.40	C_15_H_20_O_10_	359.09864	123, 182, 166, 197	360.10565	360.10594	0.29
2	*Fraxinus excelsior*	nd.	0.52	*	792.08522	191, 248, 263, 298, 354, 422, 438, 453, 468, 630			
3	*Salix phylicifolia*	205, 290	0.66	C_15_H_12_O_8_	319.04573	109, 125, 151, 193	320.05322	320.05303	−0.19
4	*Tilia sp.*	230, 291	0.67	C_21_H_22_O_12_	465.10378	107, 151, 285, 303	466.11113	466.11108	−0.05
5	*Fraxinus excelsior*	205, 225 (sh), 329	0.67	C_18_H_22_O_12_	429.10425	163, 191, 206, 221	430.11113	430.11155	0.42
6	*Acer platanoides*	258	0.70	C_22_H_24_O_14_	511.1103	125, 169, 182, 197, 313, 345	512.11661	512.11760	0.99
7	*Acer platanoides*	258	0.79	C_22_H_24_O_14_	511.11011	125, 169, 182, 197, 313, 345	512.11661	512.11741	0.8
8	*Acer platanoides*	258	0.88	C_22_H_24_O_14_	511.11000	125, 169, 182, 197, 313, 345	512.11661	512.11730	0.69
9	*Fraxinus excelsior*	198, 230, 290 (sh), 328	0.94	C_23_H_26_O_11_	477.14055	133, 161, 179, 315	478.14752	478.14785	0.335
10	*Syringa vulgaris*	195, 237, 282 (sh), 333 (sh)	0.96	C_24_H_30_O_13_	525.16106	121, 139, 165, 183, 209, 227, 249, 363, 389	526.16865	526.16836	−0.285
11	*Acer platanoides*	225, 278	0.98	C_29_H_28_O_18_	663.12130	125, 169, 313, 465, 511	664.12757	664.12860	1.03
12	*Fraxinus excelsior*	203, 231, 295 (sh), 330	1.00	C_23_H_26_O_11_	477.14055	133, 161, 179, 315	478.14752	478.14785	0.335
13	*Sorbus sp.*	nd.	1.04	C_16_H_18_O_9_	353.08783	135, 161, 179, 191	354.09509	354.09513	0.045
14	*Syringa vulgaris*	193, 237, 278 (sh), 333 (sh)	1.10	C_24_H_30_O_12_	509.16640	121, 139, 165, 233, 277, 347	510.17373	510.17370	−0.03
15	*Acer platanoides*	221, 278	1.15	C_55_H_40_O_34_	1243.13081 ^a^	125, 169, 393 ^b^, 469 ^b^, 617, 769	1244.14011	1244.13990	−0.21
16	*Syringa vulgaris*	nd.	1.31	C_27_H_38_O_15_	601.21324	153, 197	602.22108	602.22054	−0.535
17	*Syringa vulgaris*	243, 278 (sh)	1.44	C_32_H_38_O_14_	645.21930	121, 139, 149, 165, 209, 275, 329, 413	646.22616	646.22660	0.44
18	*Prunus padus*	315	1.56	C_31_H_38_O_18_	697.19783	117, 145, 163	698.20582	698.20513	−0.69
19	*Populus tremula*	193, 221, 243, 297 (sh), 328	1.60	C_23_H_22_O_10_	457.11453	133, 135, 161, 179, 235, 295, 397	458.12130	458.12183	0.53
20	*Salix phylicifolia*	240, 270	1.60	C_27_H_28_O_11_	527.15605	93, 109, 121, 137, 155, 405	528.16317	528.16335	0.185
21	*Prunus padus*	nd.	1.75	C_28_H_26_O_11_	537.14196	119, 151, 177, 271	538.14752	538.14926	1.745
22	*Populus tremula*	233, 290 (sh), 314	1.89	C_23_H_22_O_8_	425.12500	93, 117, 119, 145, 163, 219, 365	426.13147	426.13230	0.83
23	*Populus tremula*	236, 296 (sh), 319	1.93	C_24_H_24_O_9_	455.13543	117, 119, 134, 145, 160, 163, 175, 193, 395	456.14204	456.14273	0.695
24	*Populus tremula*	240, 293 (sh), 325	1.97	C_25_H_26_O_10_	485.14578	134, 149, 160, 175, 193, 207, 425	486.15260	486.15308	0.48
25	*Alnus glutinosa*	nd.	2.35	C_35_H_58_O_10_	637.39620	389, 477, 521, 605	638.40300	638.40350	0.5
26	*Alnus glutinosa*	nd.	2.41	C_35_H_58_O_10_	637.39641	389, 477, 521, 605	638.40300	638.40371	0.71
27	*Sorbus sp.*	309	2.49	C_39_H_54_O_7_	633.38042	117, 119, 145, 163, 571, 589, 615	634.38696	634.38772	0.765
28	*Tilia sp.*	nd.	2.64	C_13_H_28_O_8_	311.16894	119, 183	312.17842	312.17624	−2.18
29	*Sorbus sp.*	322	2.66	C_39_H_54_O_7_	633.38031	133, 135, 161, 179	634.38696	634.38761	0.655
30	*Sorbus sp.*	nd.	2.67	C_30_H_46_O_4_	469.33235	423	470.33961	470.33965	0.04
31	*Alnus glutinosa*	nd.	2.72	C_35_H_58_O_8_	605.40623	283, 345, 455, 473	606.41317	606.41353	0.36
32	*Alnus glutinosa*	nd.	2.79	C_36_H_60_O_8_	619.42097	343, 345, 371, 389, 487	620.42882	620.42827	−0.55
33	*Alnus glutinosa*	nd.	2.82	C_35_H_58_O_8_	605.40664	283, 345, 455, 473	606.41317	606.41394	0.77
34	*Alnus glutinosa*	nd.	2.88	C_36_H_60_O_8_	619.42214	343, 345, 371, 389, 487	620.42882	620.42944	0.62
35	*Sorbus sp.*	nd.	2.93	C_32_H_48_O_5_	511.34265	451	512.35018	512.34995	−0.225
36	*Sorbus sp.*	nd.	3.00	C_30_H_46_O_3_	453.33786	nd.	454.34470	454.34516	0.465
37	*Tilia sp.*	nd.	3.04	C_18_H_32_O_4_	311.22300	111, 155, 171	312.23006	312.23030	0.24
38	*Sorbus sp.*	nd.	3.15	C_32_H_50_O_4_	497.36354	437	498.37091	498.37084	−0.07
39	*Quercus robur*	nd.	3.55	C_27_H_44_O_4_	431.31652	133, 161, 179	432.32396	432.32382	−0.14
40	*Alnus incana*	nd.	3.81	C_53_H_80_O_11_	891.56326	117, 145, 163, 271, 331, 399, 417, 491, 745	892.57007	892.57056	0.495
41	*Alnus incana*	nd.	3.85	C_57_H_83_O_8_	895.60993	117, 119, 145, 163, 749	896.61662	896.61723	0.61
42	*Alnus incana*	nd.	3.89	C_46_H_76_O_6_	723.56173	117, 145, 223, 267, 285, 297, 455, 705	724.56419	724.56903	4.84

^a^ = Utilised in the fingerprinting method as a [2M–H]^2–^ ion at *m/z* 621.06265. ^b^ = Doubly charged ion. * = The exact molecular formula could not be predicted, but the compound seems to contain an odd number of nitrogen atoms. sh = shoulder. nd. = not detected.

**Table 3:** Selected ion recording parameters for the LC–MS fingerprinting methods.

	Method I			Method II		
Species	*m/z* Value	Cone Voltage (V)	RT Range (min)	*m/z* Value	Cone Voltage (V)	RT Range (min)
*Acer platanoides*	359.0	30	0.25–1.50	359.0	30	0.25–4.00
	511.0	50		469.0	30	
	621.0	30		545.0	50	
	663.0	50		712.6	50	
*Alnus glutinosa*	605.4	50	2.00–3.40	605.4	50	2.00–3.40
	637.4	50		619.0	50	
	661.4	50		637.4	50	
*Alnus incana*	723.5	70	3.40–4.00	439.4	30	2.40–4.00
	891.5	70		453.4	30	
	895.6	70		471.4	30	
*Fraxinus excelsior*			0.30–1.30	369.0	30	0.20–3.50
	429.0	10		455.0	30	
	477.0	50		471.0	30	
	792.0	50		477.0	30	
				623.0	50	
*Populus tremula*	425.0	30	1.00–2.25	441.0	30	1.20–2.25
	455.0	30		457.0	30	
	457.0	30		471.0	30	
	485.0	30				
*Prunus padus*			1.00–2.00	455.0	30	1.00–3.50
	537.0	30		471.0	30	
	697.0	50		497.0	30	
				697.0	50	
*Quercus robur*	431.0	50	0.25–3.80	217.0	30	0.20–4.50
				289.0	30	
				431.0	50	
				439.0	30	
*Salix phylicifolia*			0.40–1.90	217.0	30	0.30–4.50
	319.0	30		301.0	30	
	527.0	30		503.4	30	
				712.5	30	
*Sorbus hybrida*	653.0	50	0.40–3.20			
	1045.6	50				
*Sorbusaucuparia* and *Sorbus hybrida*			0.60–2.80	353.0	30	0.80–3.50
				453.4	30	
	425.0	30		469.4	30	
	569.0	50		497.4	30	
				511.5	30	
				633.5	30	
*Syringa vulgaris*	509.0	30	0.60–1.70	455.4	30	0.80–3.20
	525.0	30		471.0	30	
	601.0	30		539.0	30	
	645.0	30				
*Tilia* sp.			0.50–3.25	217.0	30	0.25–4.50
	311.0	30		289.0	30	
	465.0	30		475.4	30	
				503.6	30	

## Data Availability

The data presented in this study are available on request from the corresponding author.

## Supplementary Materials

The following supporting information can be downloaded at https://www.mdpi.com/article/10.3390/molecules27206810/s1, Figures S1–S13: Orbitrap and QqQ full scan spectra for all species and the repeatability of fingerprinting. Figures S14–S60: Fingerprints of all studied species with species-specific methods I and II. Figures S61–S95 and Tables S1–S36: MS/MS spectra, fragmentation patterns, *m/z* values, and other mass spectrometric information of the fragments of the markers (1–42) used in the final fingerprinting methods.

## Sample Availability

Samples of the compounds are not available from the authors.
