# Supplementary material for: Chemotaxonomic Markers for the Leaf Buds of Common Finnish Trees and Shrubs: A Rapid UHPLC MS Fingerprinting Tool for Species Identification
Source: Molecules. 2022 Oct 11;27(20):6810. doi: 10.3390/molecules27206810 (PMC9611062; doi:10.3390/molecules27206810)
Supplement: Supplementary file 1 [file molecules-27-06810-s001.zip › molecules-1941198-supplementary.pdf]

Supplementary Information for

Chemotaxonomic markers for the leaf buds of common Finnish tree species: a rapid UHPLC-MS fingerprinting method for species identification

Marianna Manninen, Maarit Karonen and Juha-Pekka Salminen

Natural Chemistry Research Group, Department of Chemistry, University of Turku, FI-20014, Turku, Finland

Table of Contents

Section 1. Orbitrap and QqQ full scan spectra for all species and the repeatability of fingerprinting 1

Section 2. Fingerprints of all studied species with each species-specific method I and II..... 14

Section 3. MS/MS spectra, fragmentation patterns, *m/z* values and other mass spectrometric information of the fragments of the markers (**1-42**) used in the final fingerprinting methods 37

Section 1. Orbitrap and QqQ full scan spectra for all species and the repeatability of fingerprinting.

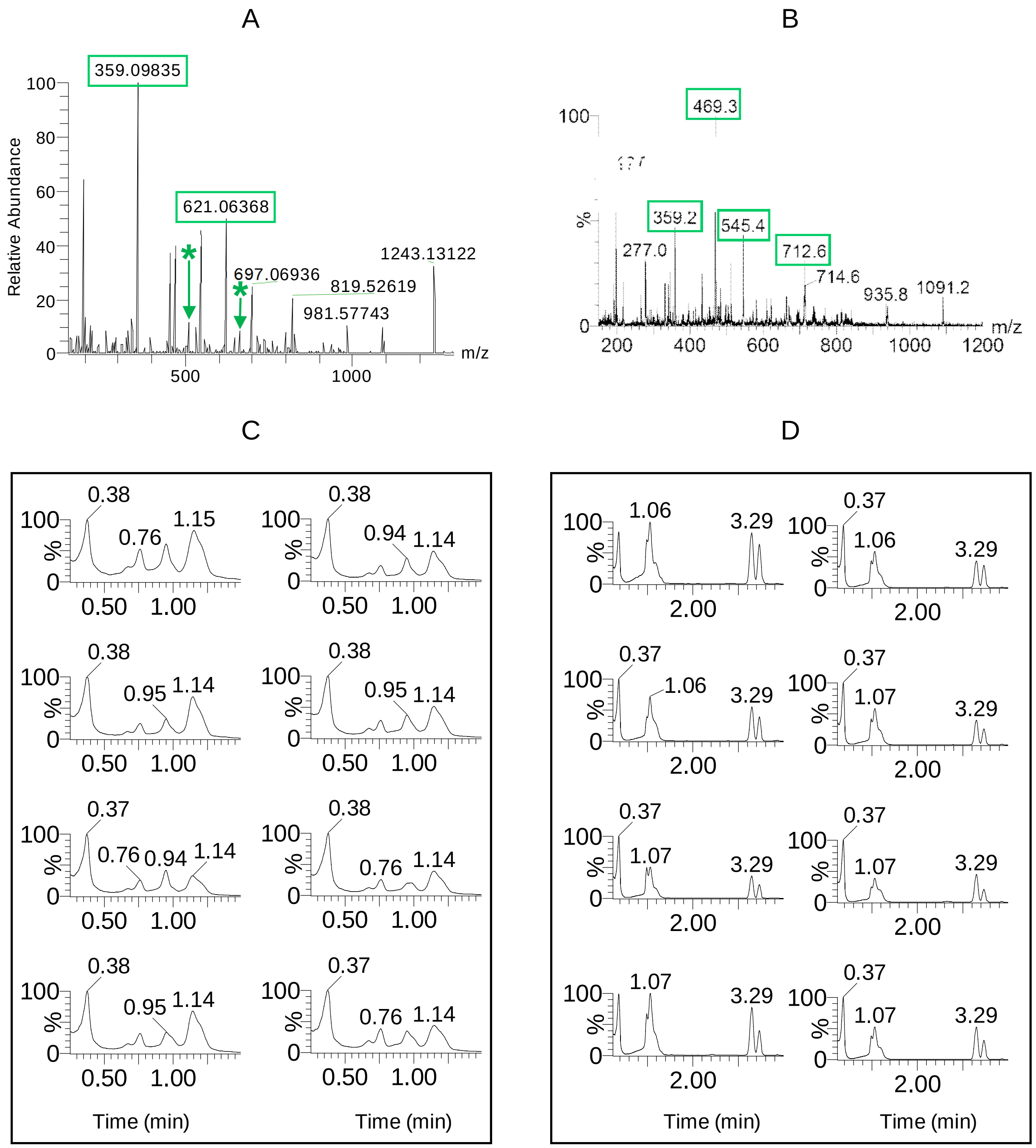

Figure S1. Full scan spectra of *Acer platanoides* from (A) Orbitrap-MS and (B) QqQ-MS. Markers used in the fingerprinting method are highlighted with a green box or an asterisk. Fingerprints of eight replicate *Acer platanoides* leaf bud extracts with (C) method I and (D) method II.

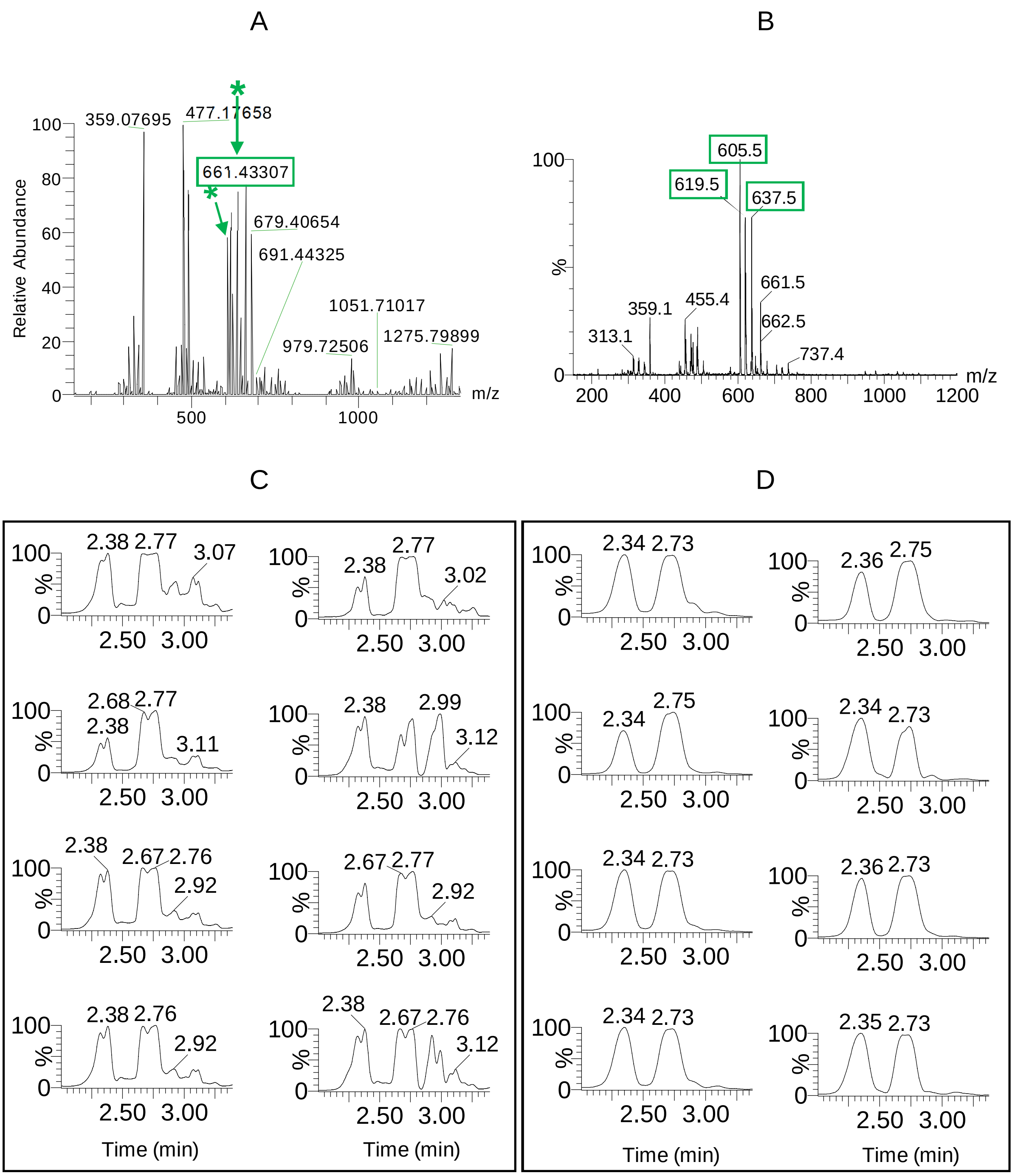

Figure S2. Full scan spectra of *Alnus glutinosa* from (A) Orbitrap-MS and (B) QqQ-MS. Markers used in the fingerprinting method are highlighted with a green box or an asterisk. Fingerprints of eight replicate *Alnus glutinosa* leaf bud extracts with (C) method I and (D) method II.

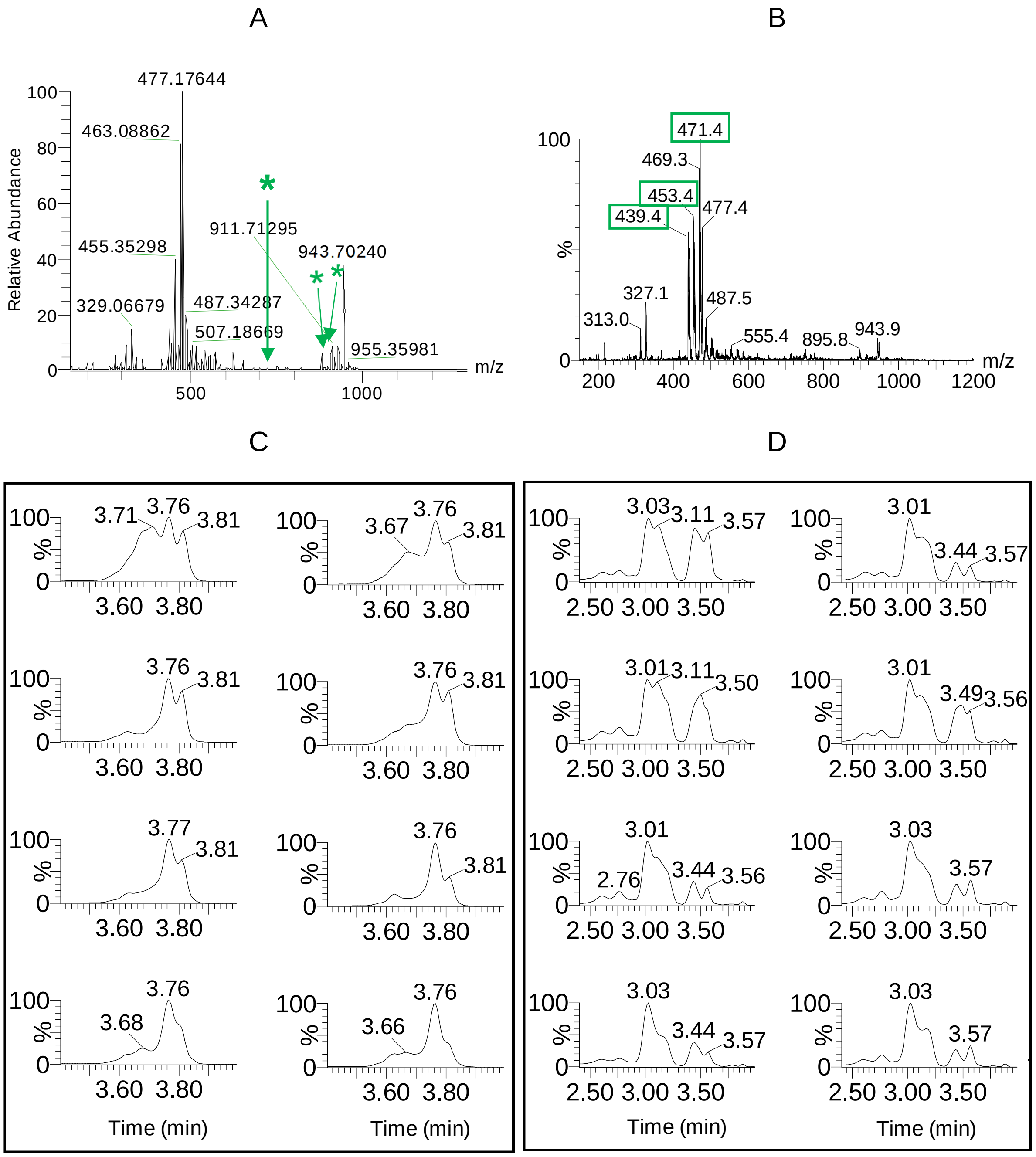

Figure S3. Full scan spectra of *Alnus incana* from (A) Orbitrap-MS and (B) QqQ-MS. Markers used in the fingerprinting method are highlighted with a green box or an asterisk. Fingerprints of eight replicate *Alnus incana* leaf bud extracts with (C) method I and (D) method II.

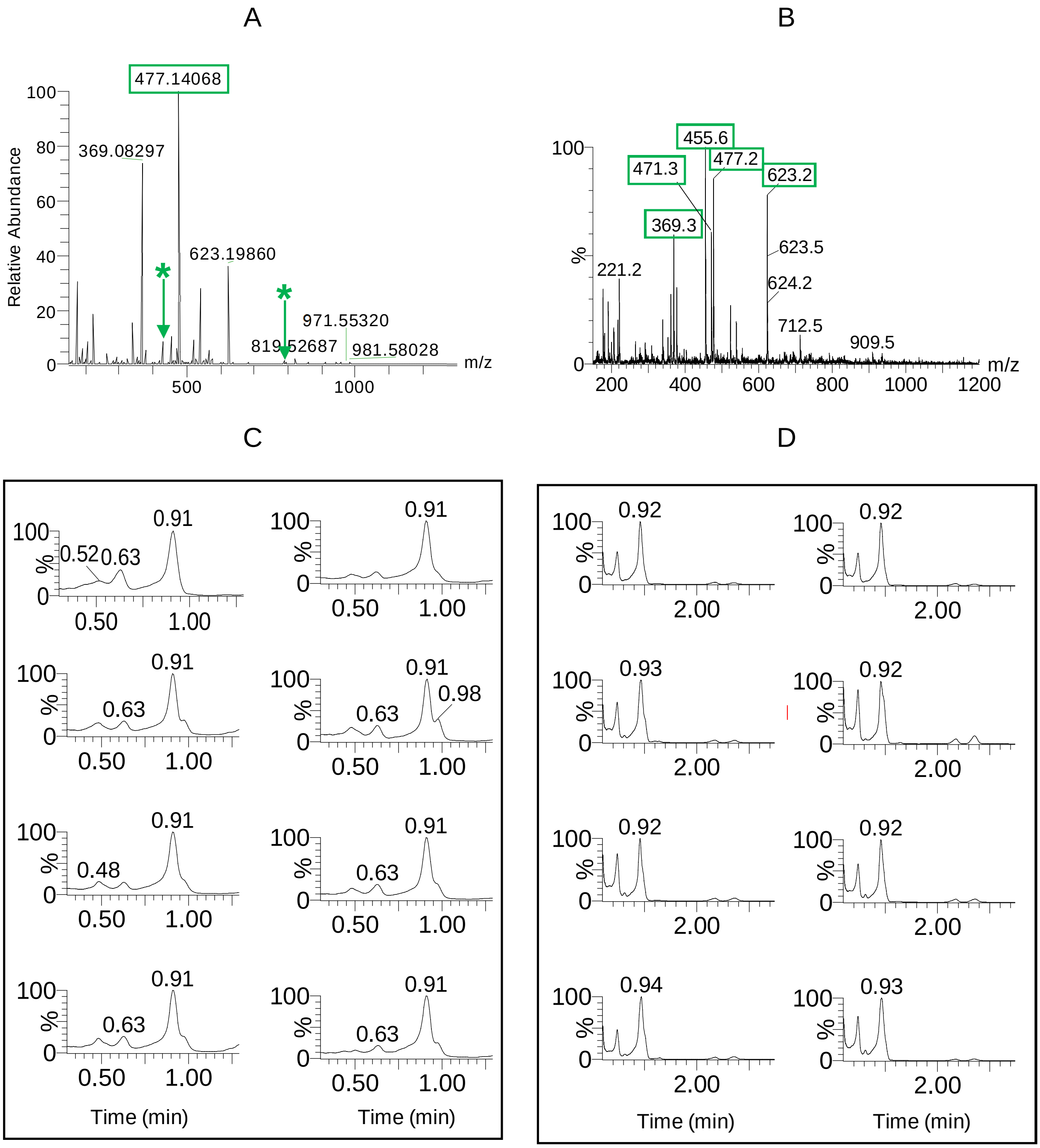

Figure S4. Full scan spectra of *Fraxinus excelsior* from (A) Orbitrap-MS and (B) QqQ-MS. Markers used in the fingerprinting method are highlighted with a green box or an asterisk. Fingerprints of eight replicate *Fraxinus excelsior* leaf bud extracts with (C) method I and (D) method II.

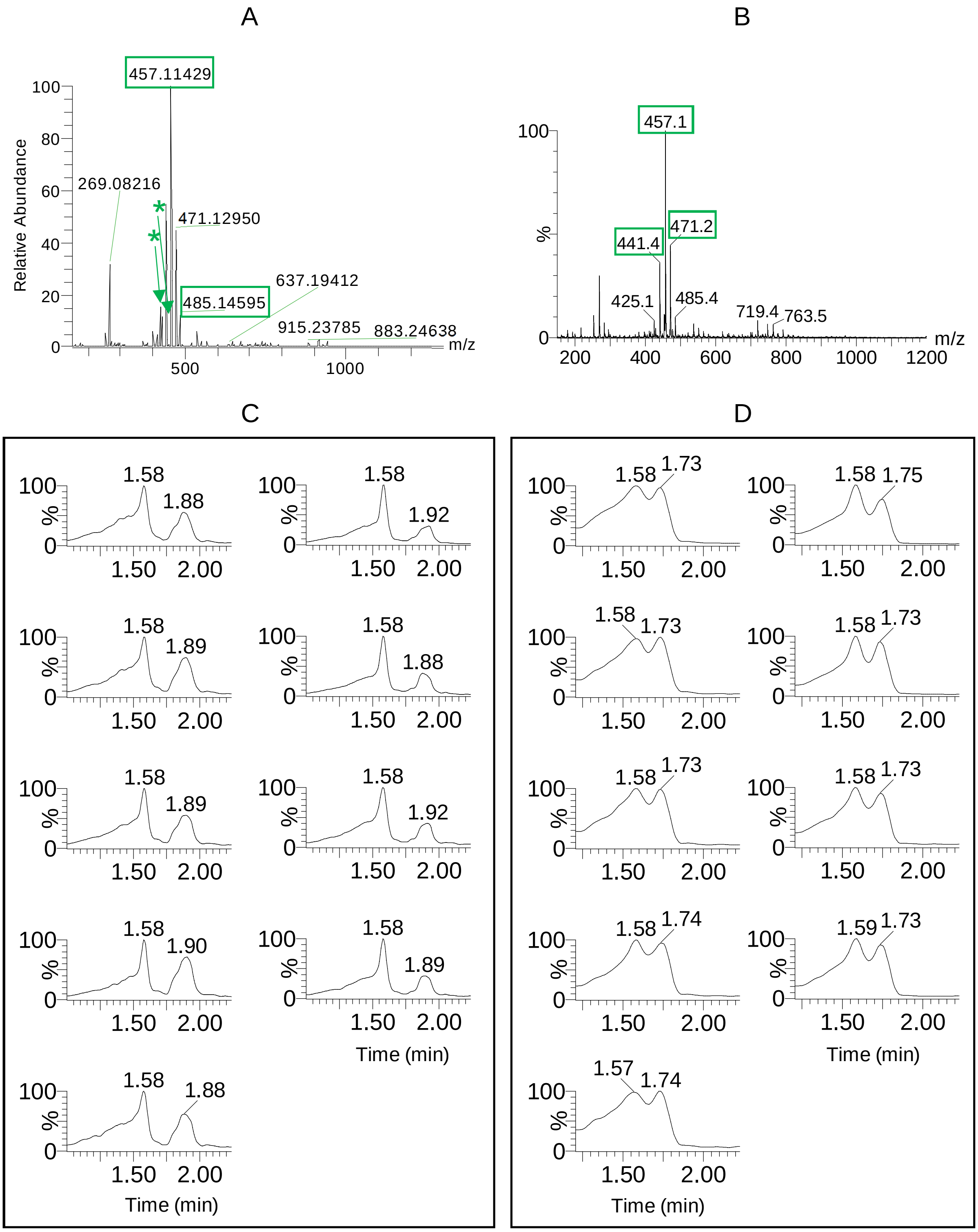

Figure S5. Full scan spectra of *Populus tremula* from (A) Orbitrap-MS and (B) QqQ-MS. Markers used in the fingerprinting method are highlighted with a green box or an asterisk. Fingerprints of nine replicate *Populus tremula* leaf bud extracts with (C) method I and (D) method II.

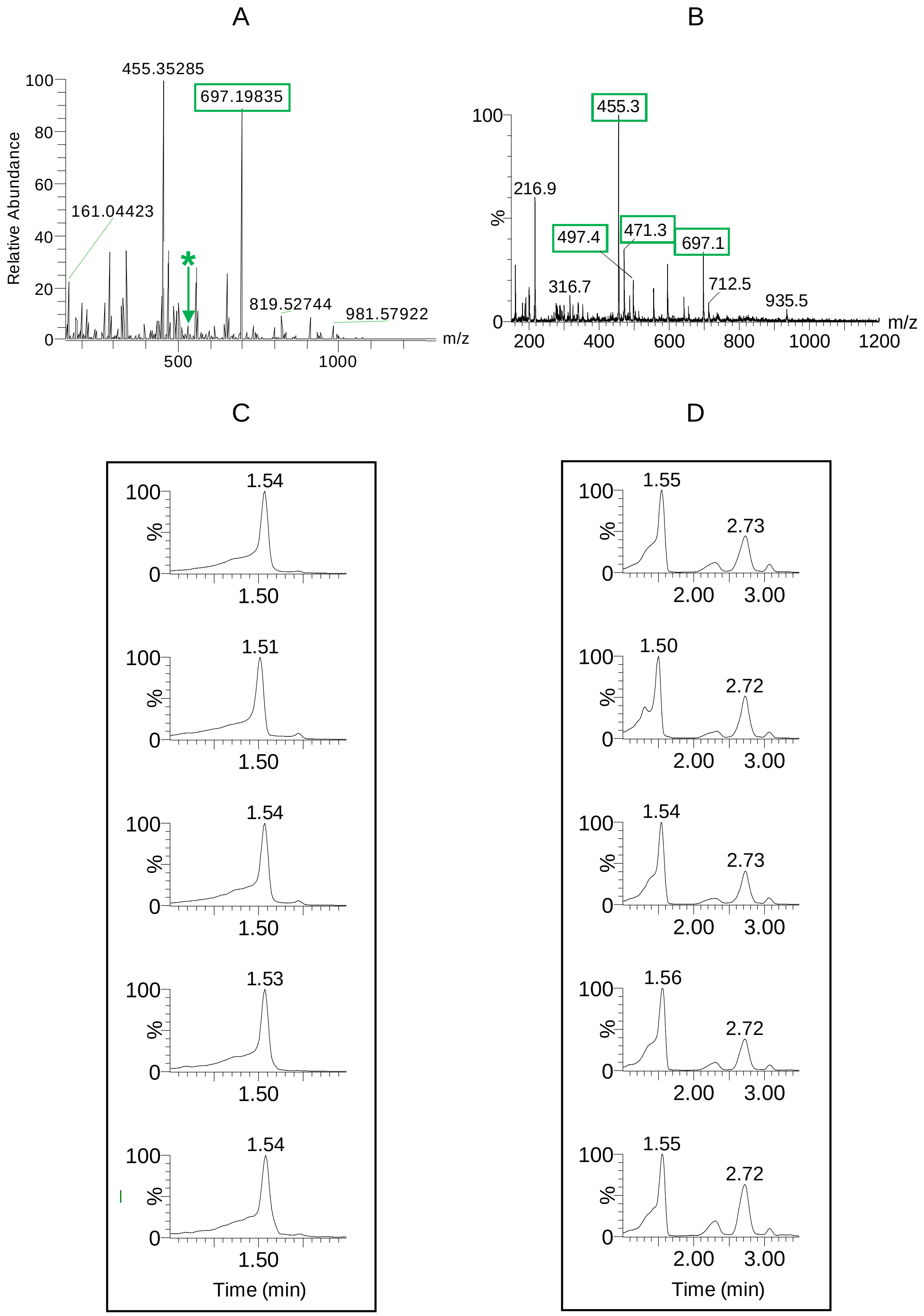

Figure S6. Full scan spectra of *Prunus padus* from (A) Orbitrap-MS and (B) QqQ-MS. Markers used in the fingerprinting method are highlighted with a green box or an asterisk. Fingerprints of five replicate *Prunus padus* leaf bud extracts with (C) method I and (D) method II.

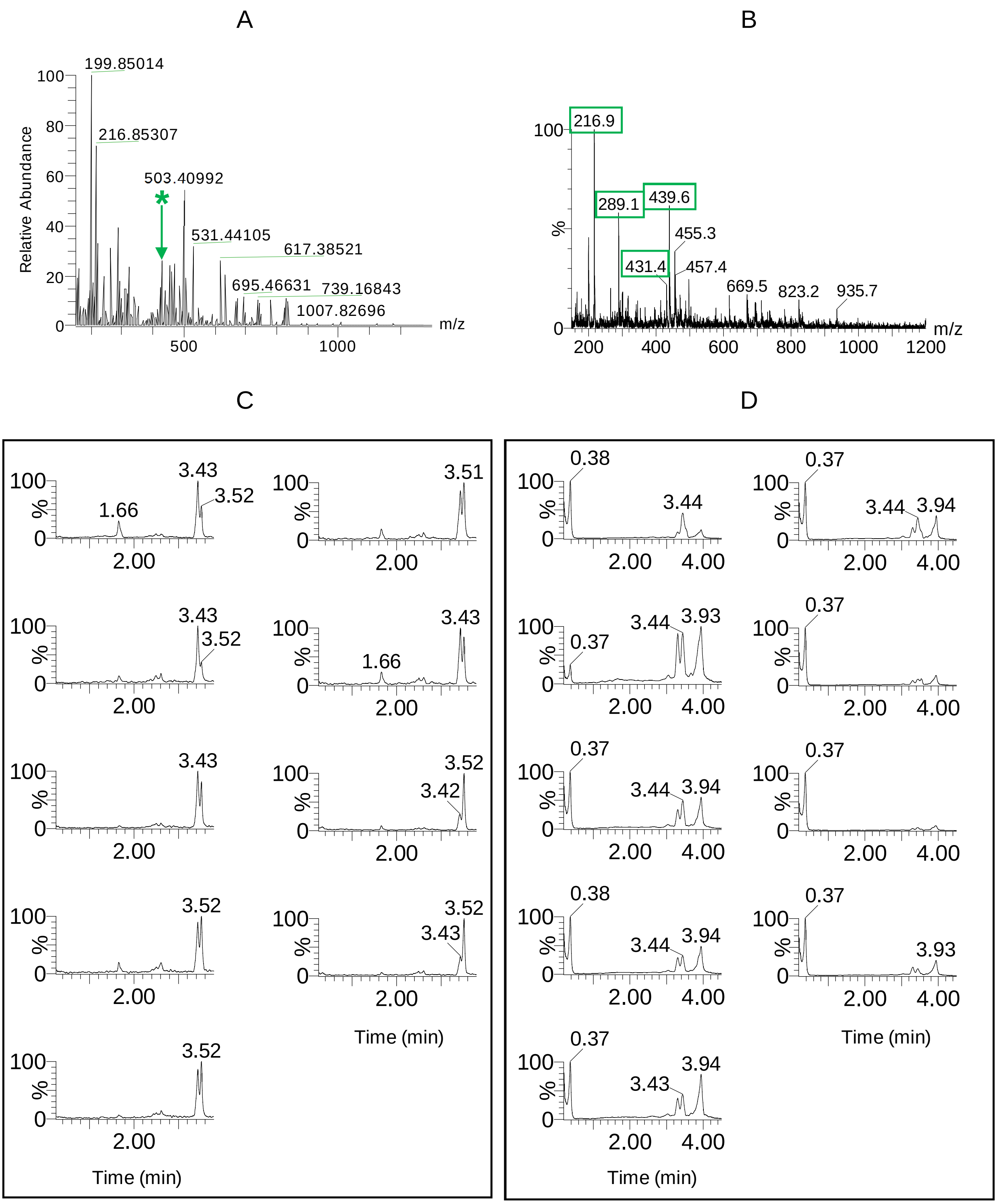

Figure S7. Full scan spectra of *Quercus robur* from (A) Orbitrap-MS and (B) QqQ-MS. Markers used in the fingerprinting method are highlighted with a green box or an asterisk. Fingerprints of nine replicate *Quercus robur* leaf bud extracts with (C) method I and (D) method II.

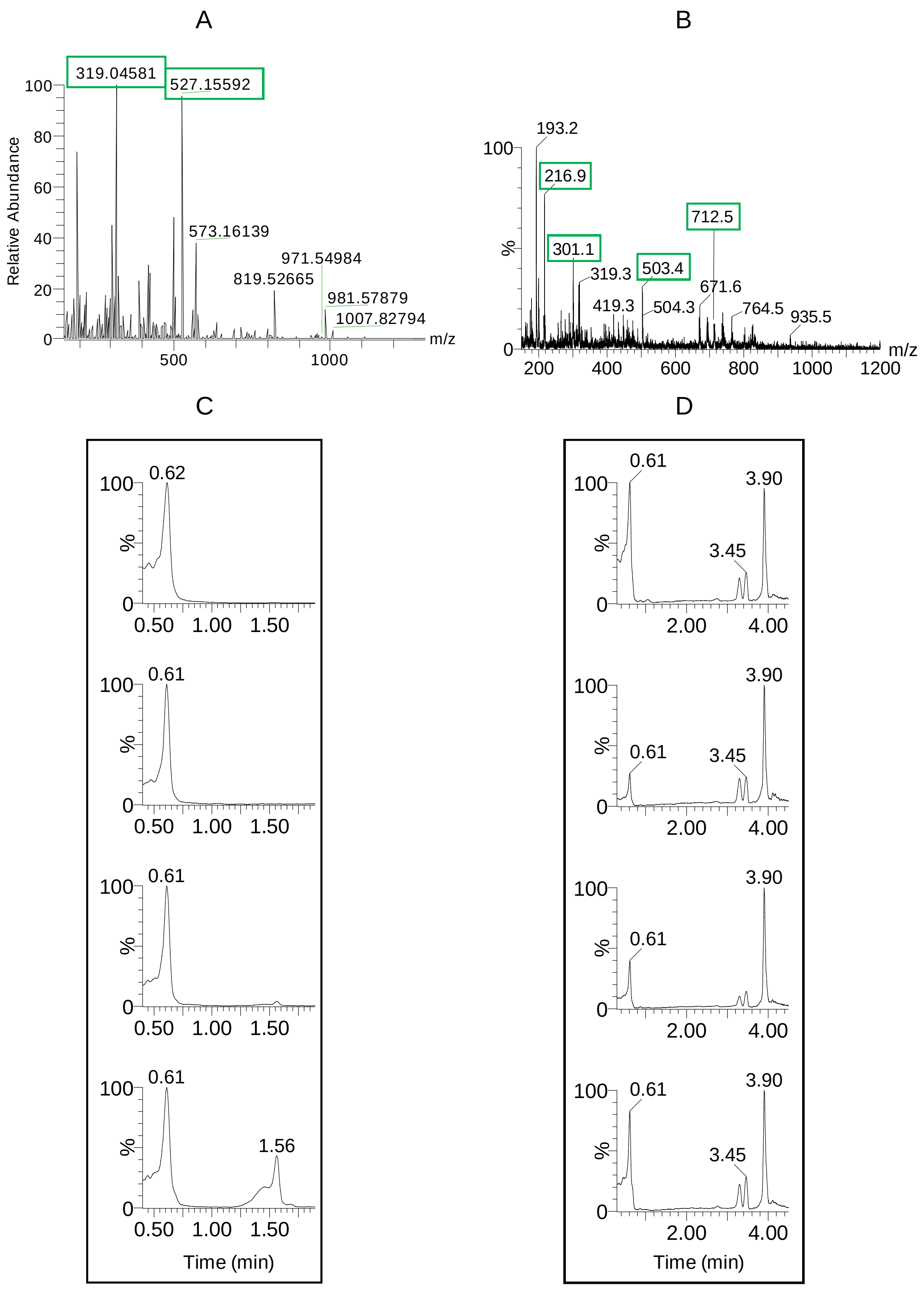

Figure S8. Full scan spectra of *Salix phylicifolia* from (A) Orbitrap-MS and (B) QqQ-MS. Markers used in the fingerprinting method are highlighted with a green box or an asterisk. Fingerprints of four replicate *Salix phylicifolia* leaf bud extracts with (C) method I and (D) method II.

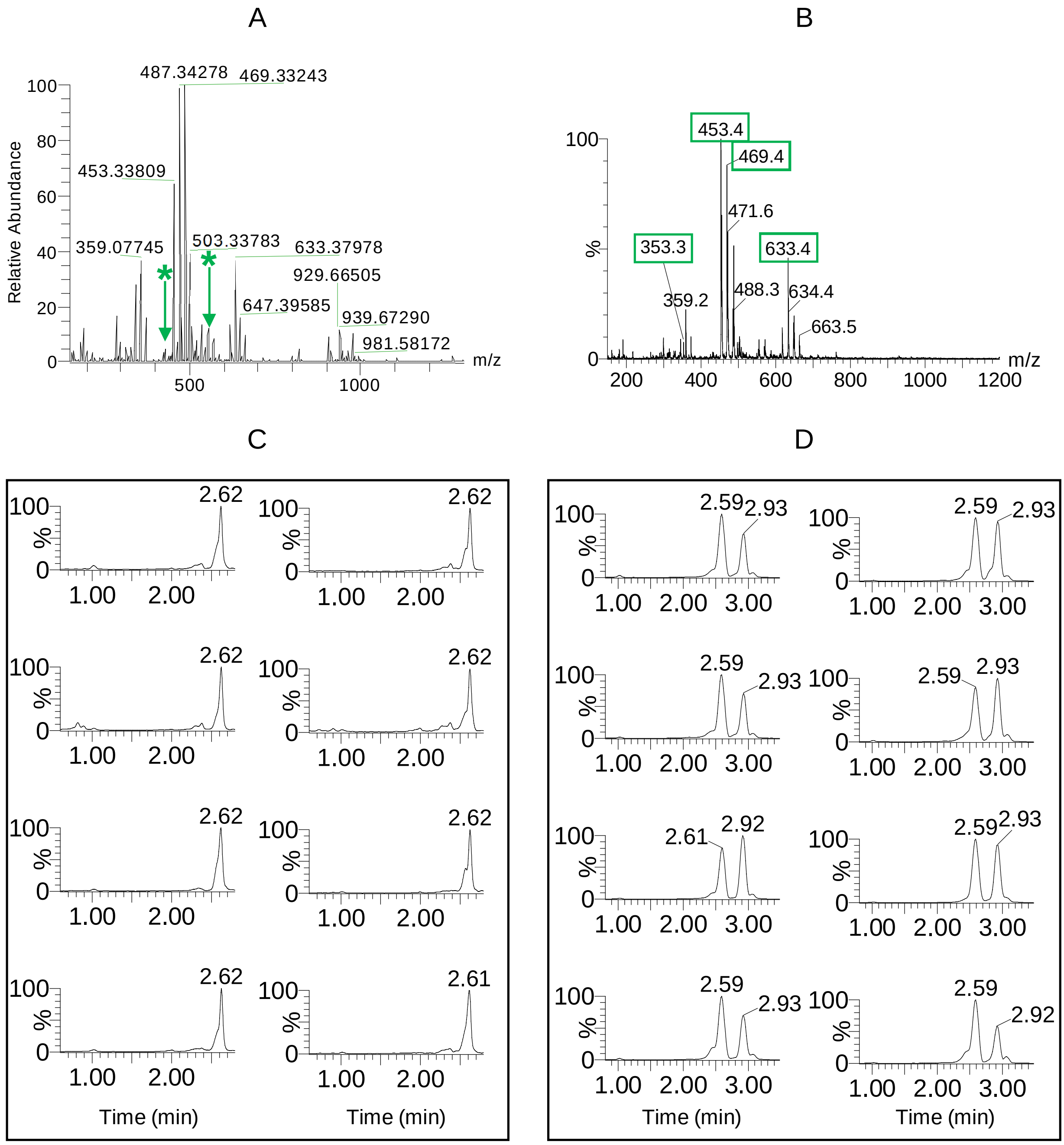

Figure S9. Full scan spectra of *Sorbus aucuparia* from (A) Orbitrap-MS and (B) QqQ-MS. Markers used in the fingerprinting method are highlighted with a green box or an asterisk. Fingerprints of eight replicate *Sorbus aucuparia* leaf bud extracts with (C) method I and (D) method II.

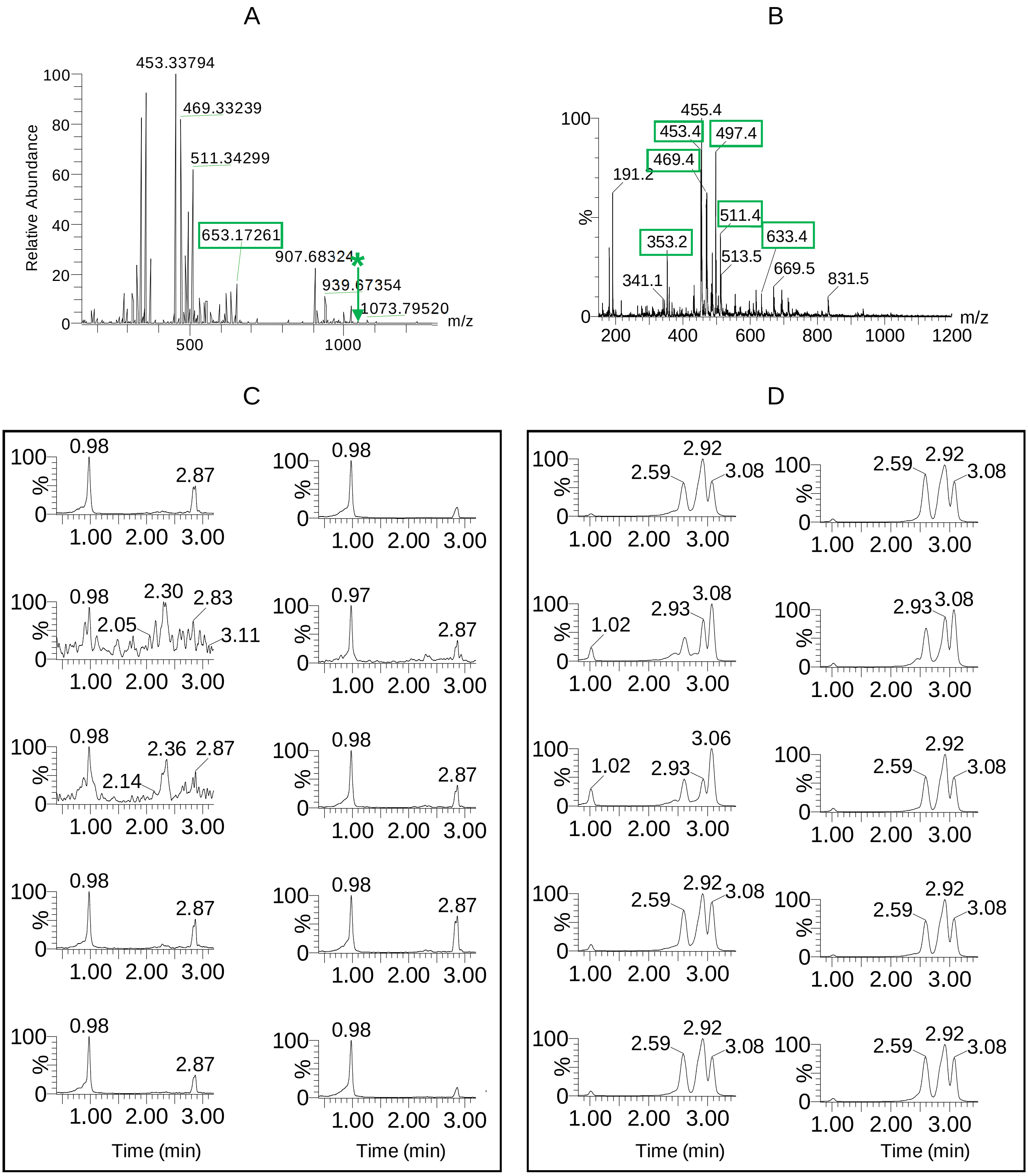

Figure S10. Full scan spectra of *Sorbus hybrida* from (A) Orbitrap-MS and (B) QqQ-MS. Markers used in the fingerprinting method are highlighted with a green box or an asterisk. Fingerprints of ten replicate *Sorbus hybrida* leaf bud extracts with (C) method I and (D) method II.

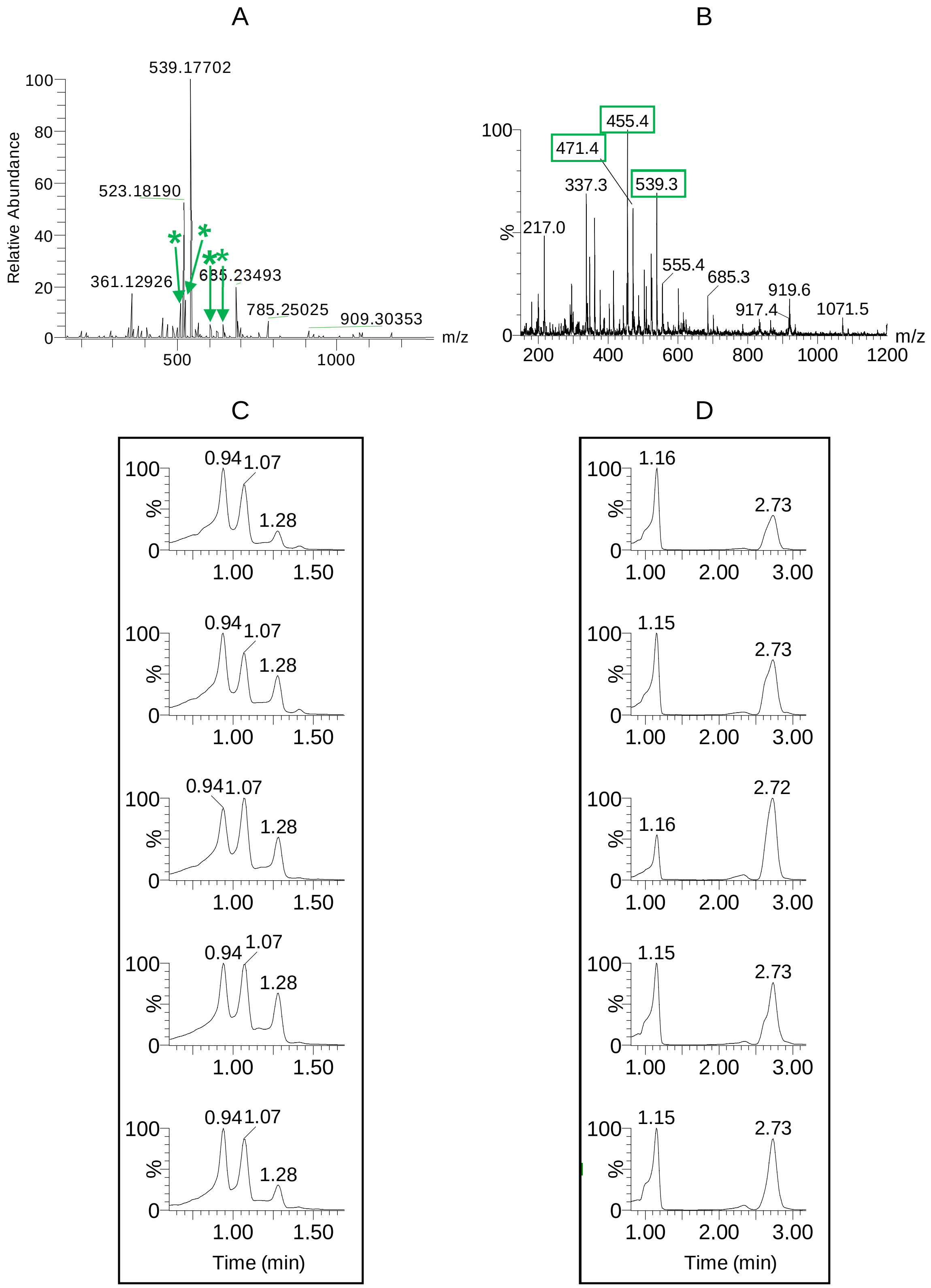

Figure S11. Full scan spectra of *Syringsa vulgaris* from (A) Orbitrap-MS and (B) QqQ-MS. Markers used in the fingerprinting method are highlighted with a green box or an asterisk. Fingerprints of five replicate *Syringsa vulgaris* leaf bud extracts with (C) method I and (D) method II.

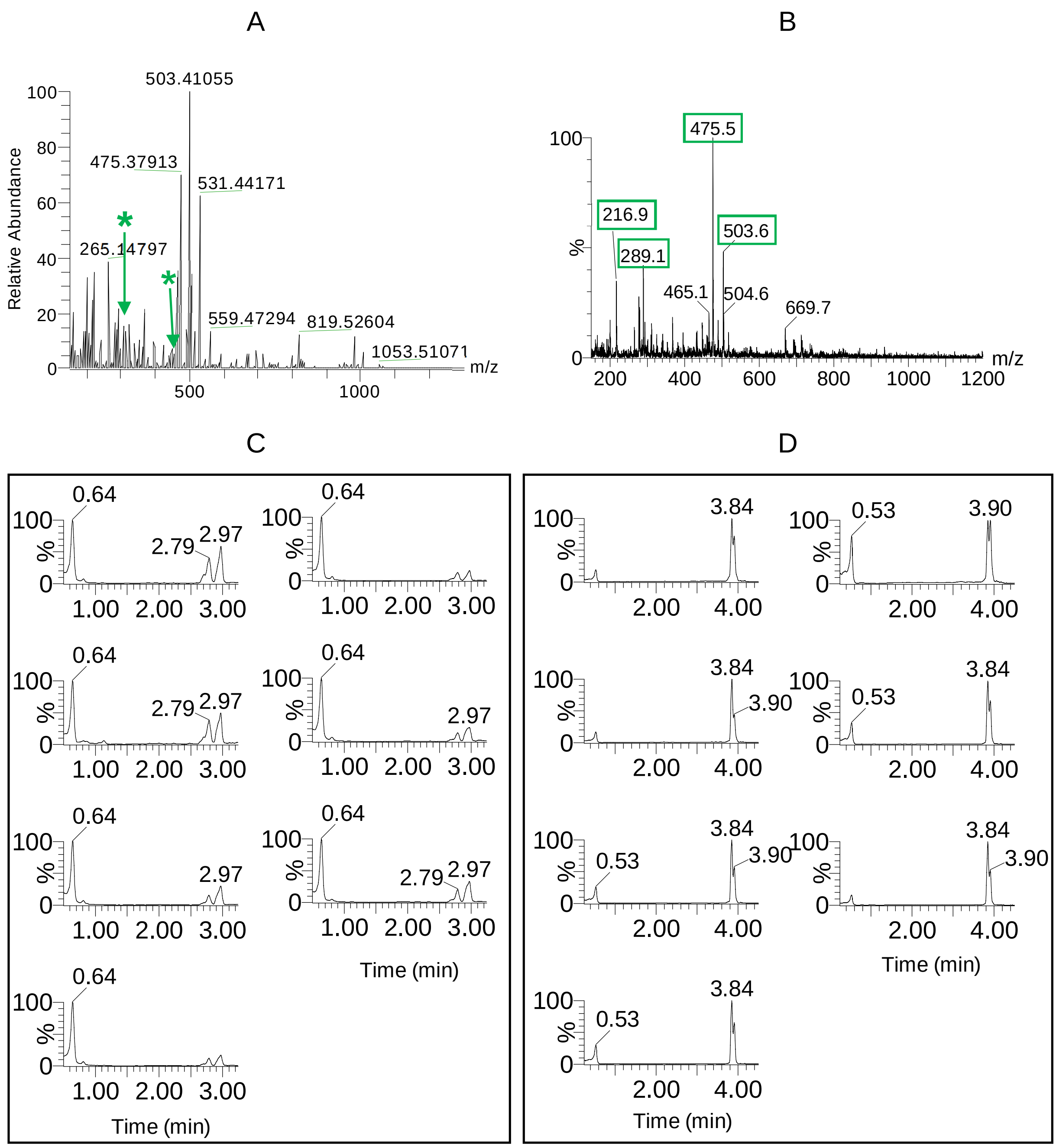

Figure S12. Full scan spectra of *Tilia cordata* from (A) Orbitrap-MS and (B) QqQ-MS. Markers used in the fingerprinting method are highlighted with a green box or an asterisk. Fingerprints of seven replicate *Tilia cordata* leaf bud extracts with (C) method I and (D) method II.

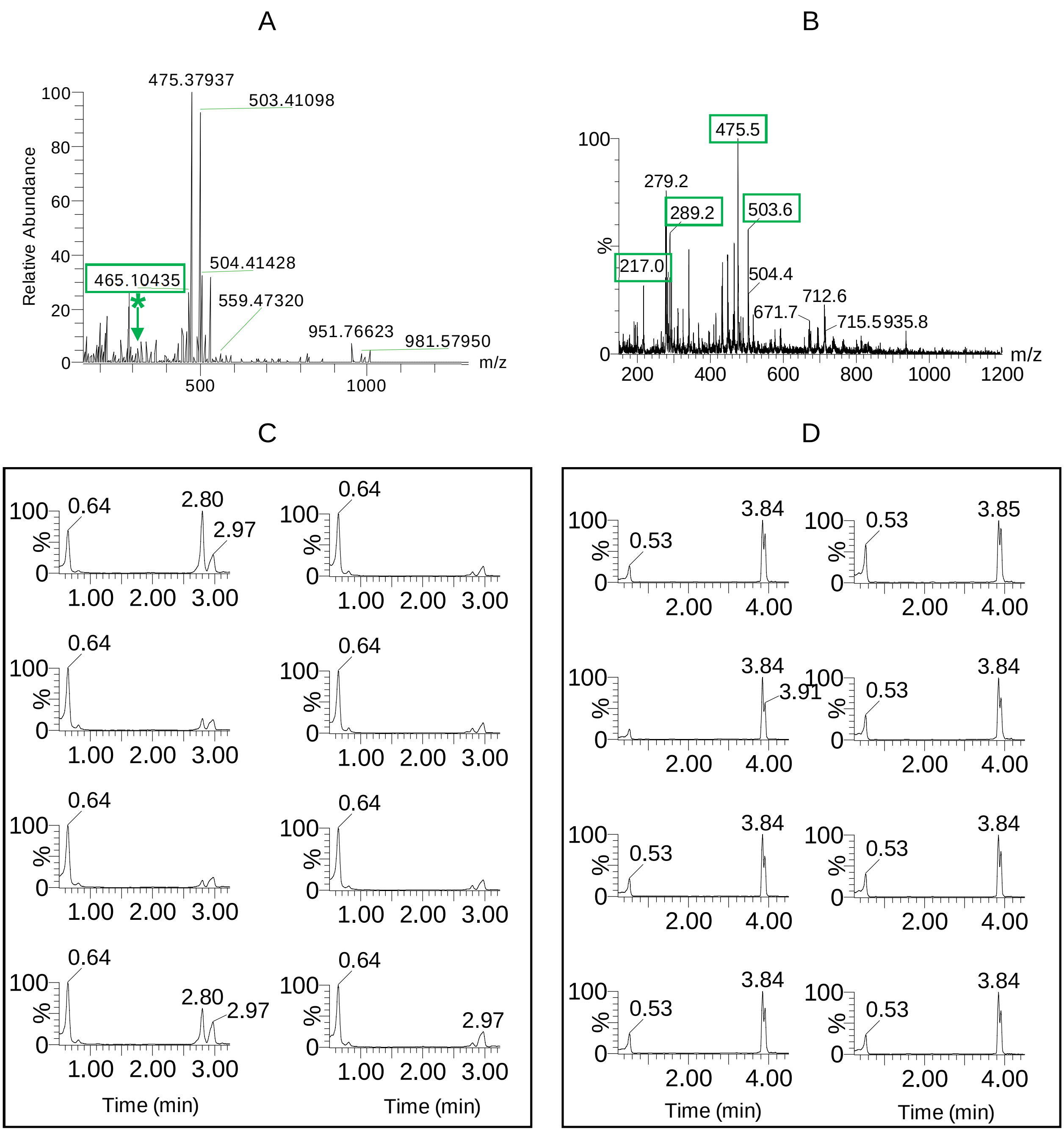

Figure S13. Full scan spectra of *Tilia* × *europaea* from (A) Orbitrap-MS and (B) QqQ-MS. Markers used in the fingerprinting method are highlighted with a green box or an asterisk. Fingerprints of eight replicate *Tilia* × *europaea* leaf bud extracts with (C) method I and (D) method II.

Section 2. Fingerprints of all studied species with each species-specific method I and II

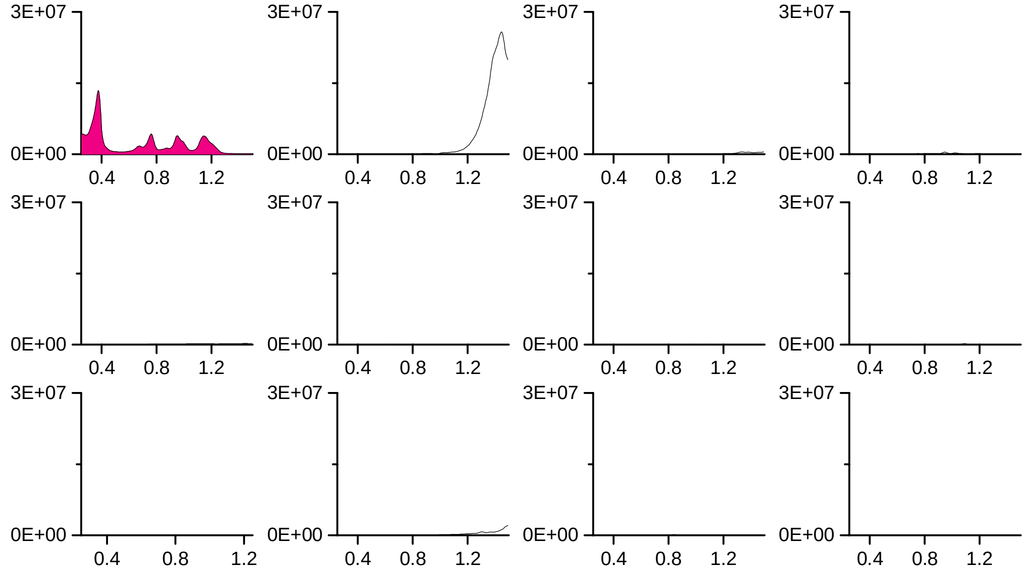

Figure S14. Fingerprints of all studied species with the species-specific method I for *Acer platanoides* (highlighted in color). The y-axes are scaled to the most intensive peak of all samples.

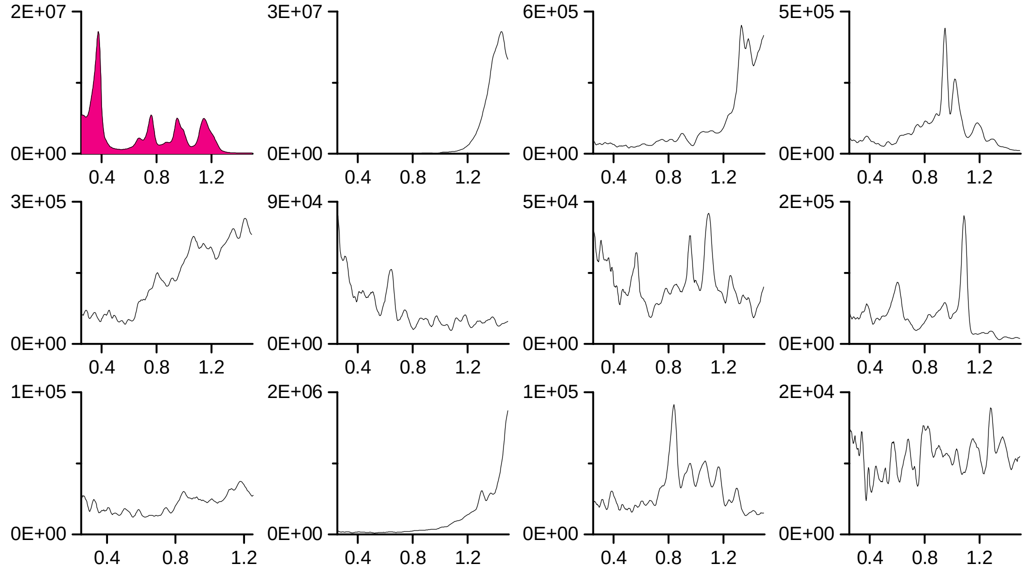

Figure S15. Fingerprints of all studied species with the species-specific method I for *Acer platanoides* (highlighted in color). The y-axes are scaled to the most intensive peak of each sample.

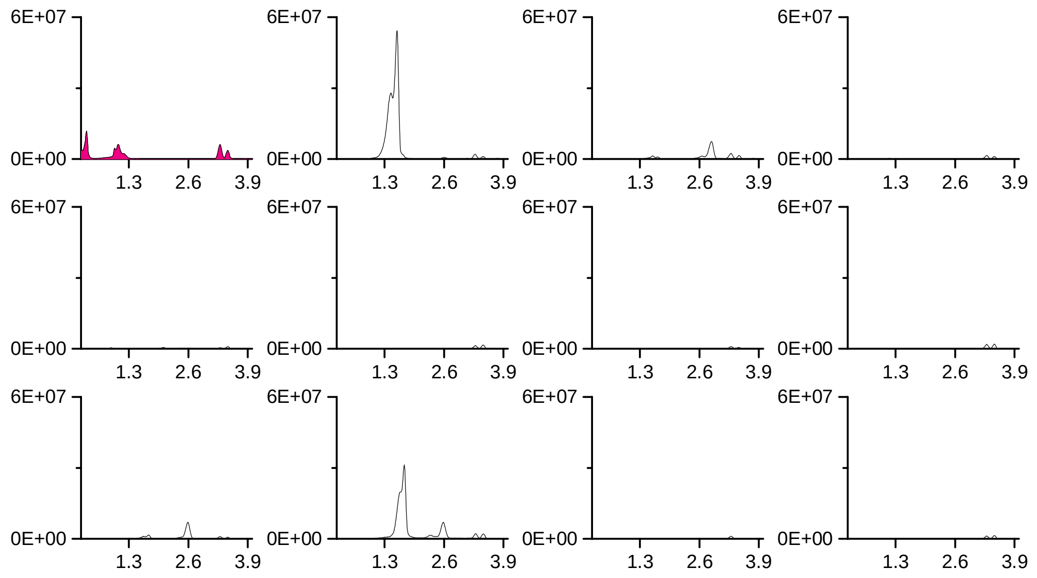

Figure S16. Fingerprints of all studied species with the species-specific method II for *Acer platanoides* (highlighted in color). The y-axes are scaled to the most intensive peak of all samples.

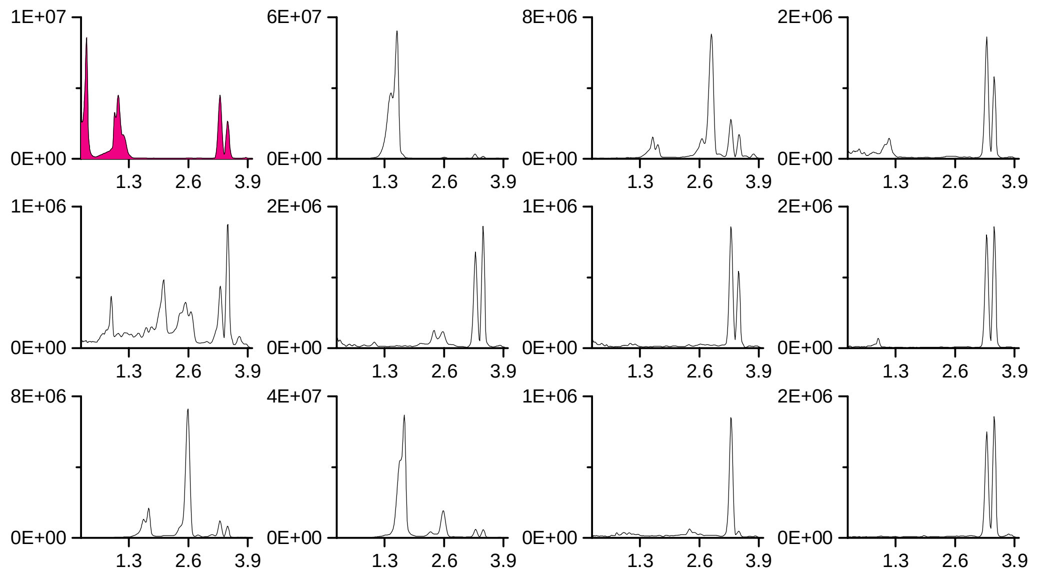

Figure S17. Fingerprints of all studied species with the species-specific method II for *Acer platanoides* (highlighted in color). The y-axes are scaled to the most intensive peak of each sample.

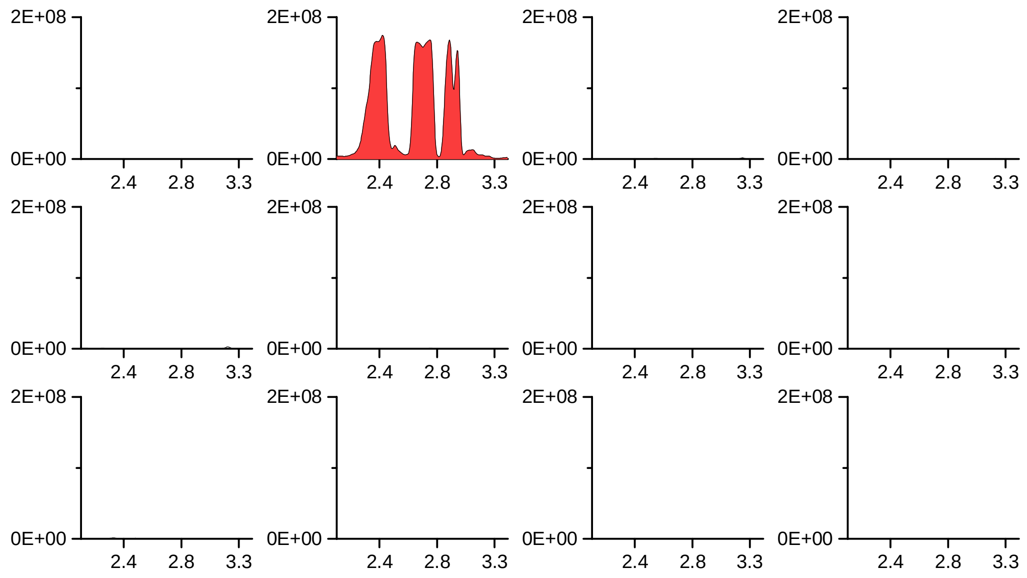

Figure S18. Fingerprints of all studied species with the species-specific method I for *Alnus glutinosa* (highlighted in color). The y-axes are scaled to the most intensive peak of all samples.

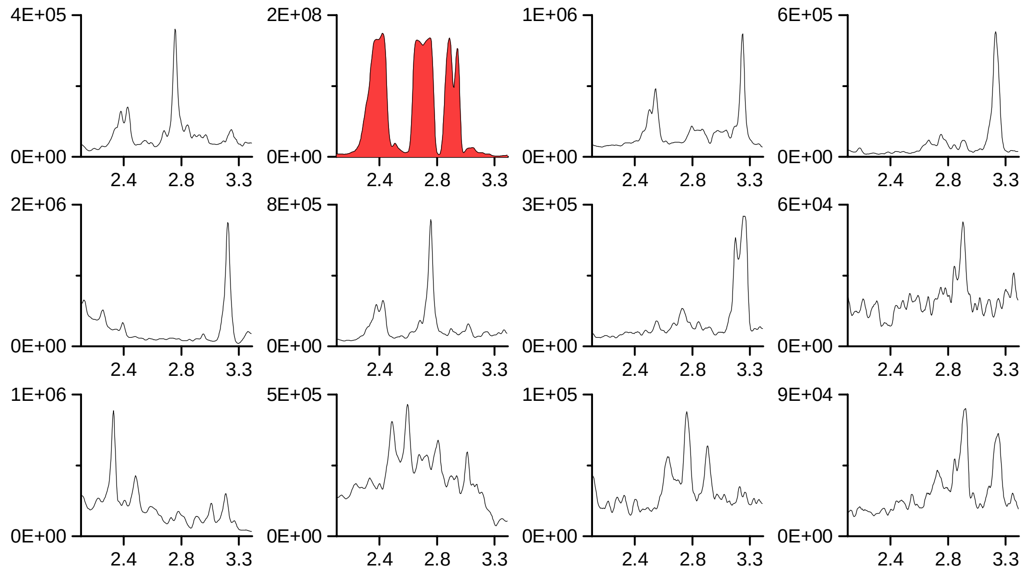

Figure S19. Fingerprints of all studied species with the species-specific method I for *Alnus glutinosa* (highlighted in color). The y-axes are scaled to the most intensive peak of each sample.

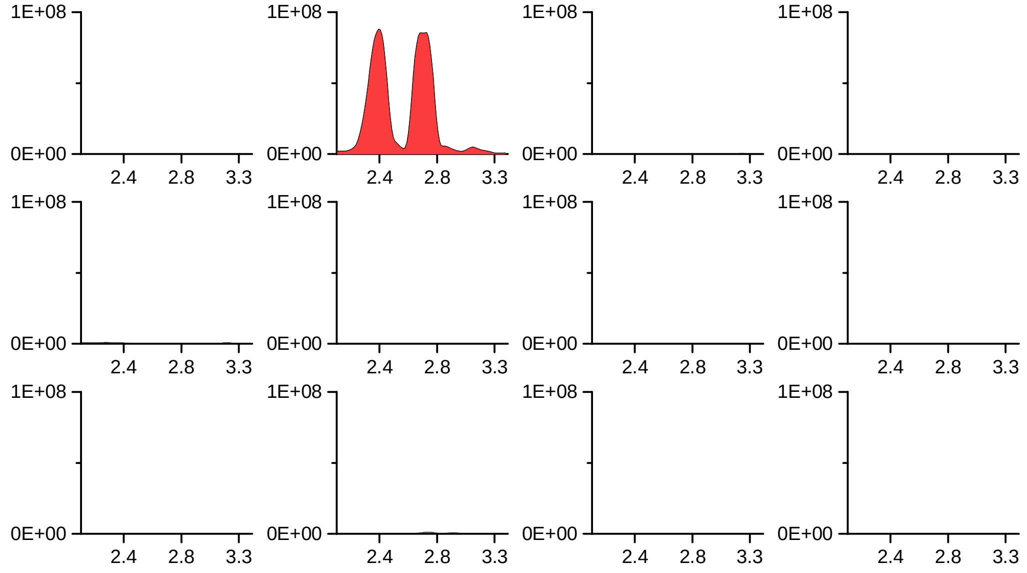

Figure S20. Fingerprints of all studied species with the species-specific method II for *Alnus glutinosa* (highlighted in color). The y-axes are scaled to the most intensive peak of all samples.

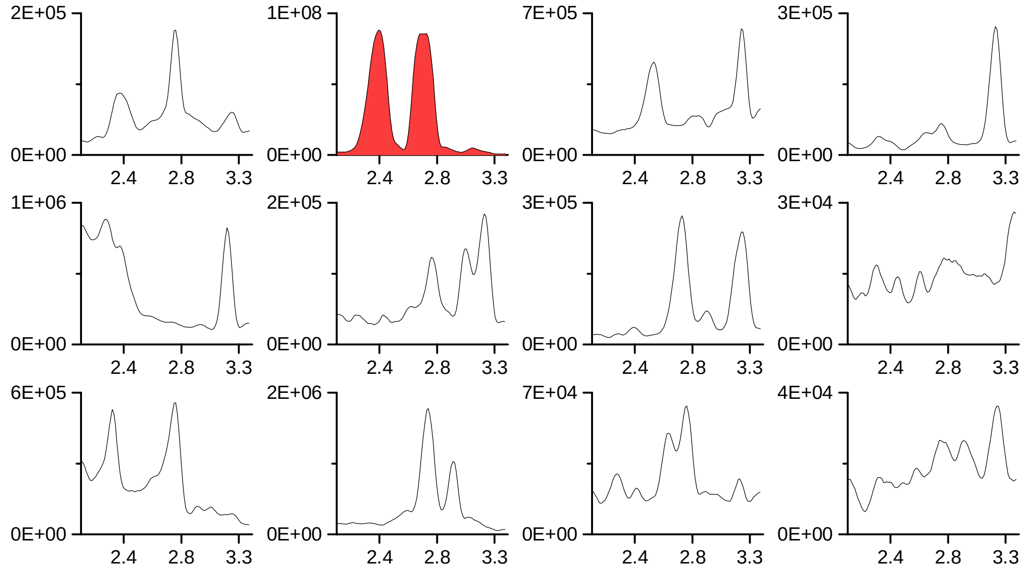

Figure S21. Fingerprints of all studied species with the species-specific method II for *Alnus glutinosa* (highlighted in color). The y-axes are scaled to the most intensive peak of each sample.

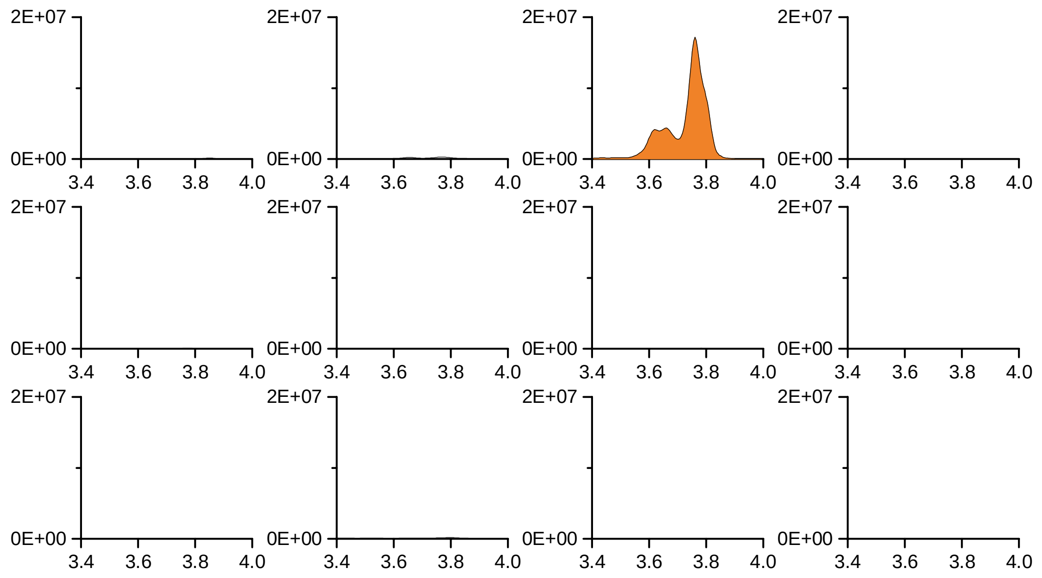

Figure S22. Fingerprints of all studied species with the species-specific method I for *Alnus incana* (highlighted in color). The y-axes are scaled to the most intensive peak of all samples.

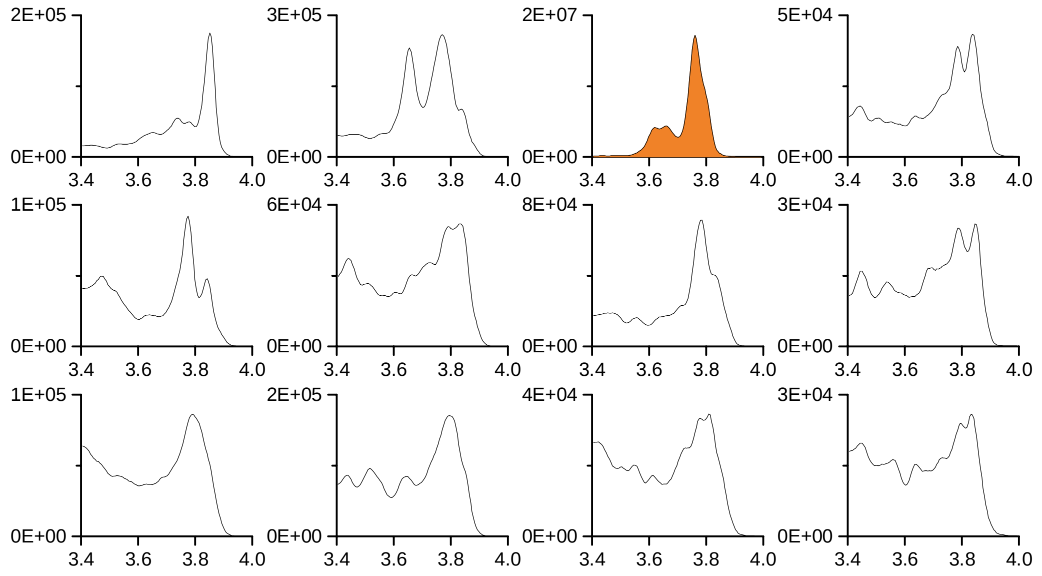

Figure S23. Fingerprints of all studied species with the species-specific method I for *Alnus incana* (highlighted in color). The y-axes are scaled to the most intensive peak of each sample.

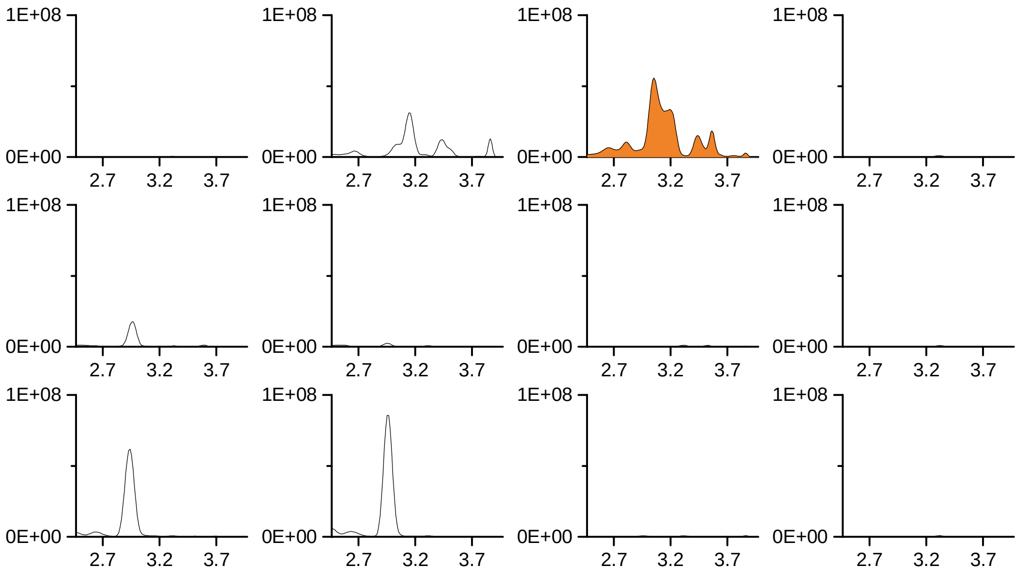

Figure S24. Fingerprints of all studied species with the species-specific method II for *Alnus incana* (highlighted in color). The y-axes are scaled to the most intensive peak of all samples.

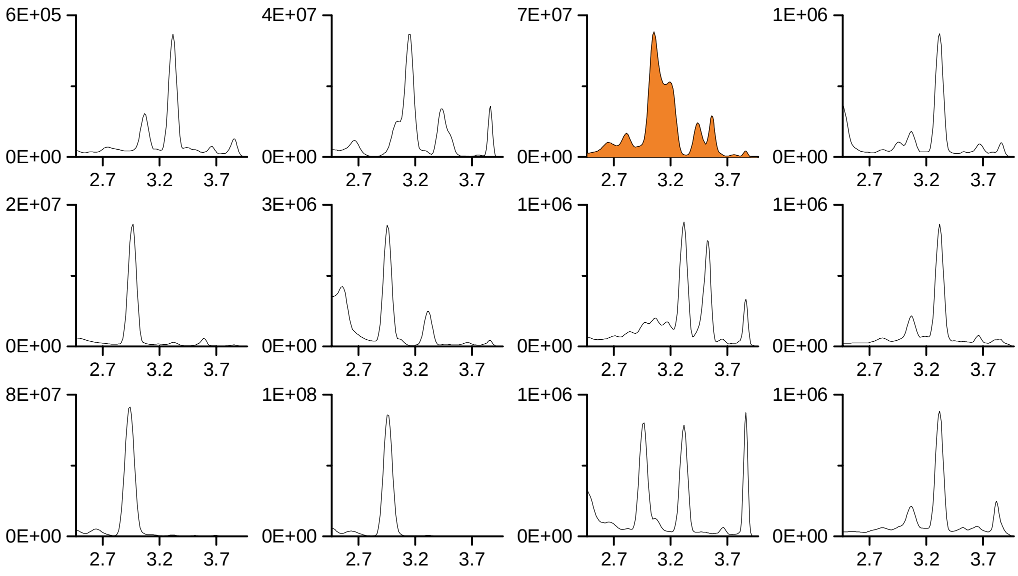

Figure S25. Fingerprints of all studied species with the species-specific method II for *Alnus incana* (highlighted in color). The y-axes are scaled to the most intensive peak of each sample.

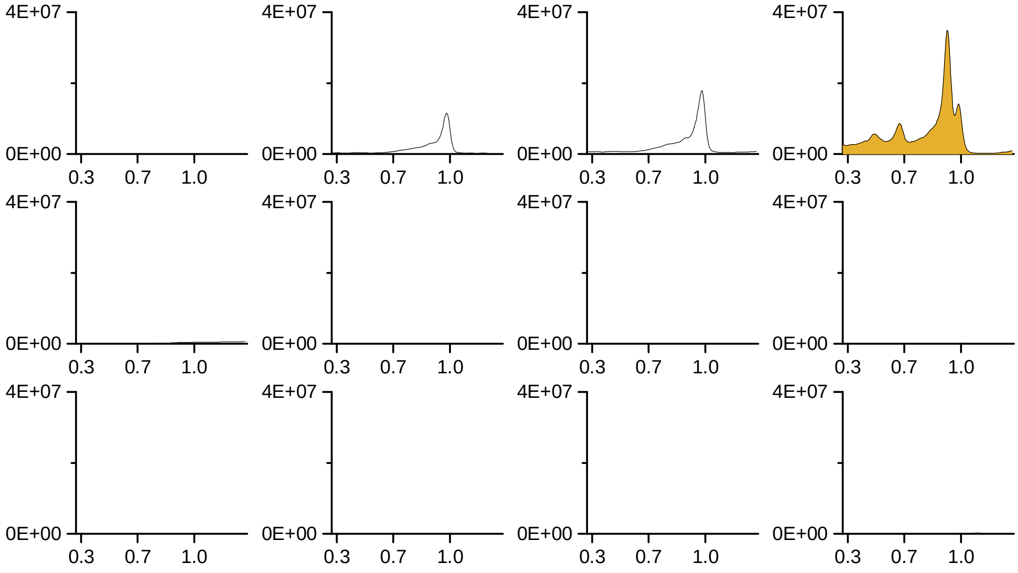

Figure S26. Fingerprints of all studied species with the species-specific method I for *Fraxinus excelsior* (highlighted in color). The y-axes are scaled to the most intensive peak of all samples.

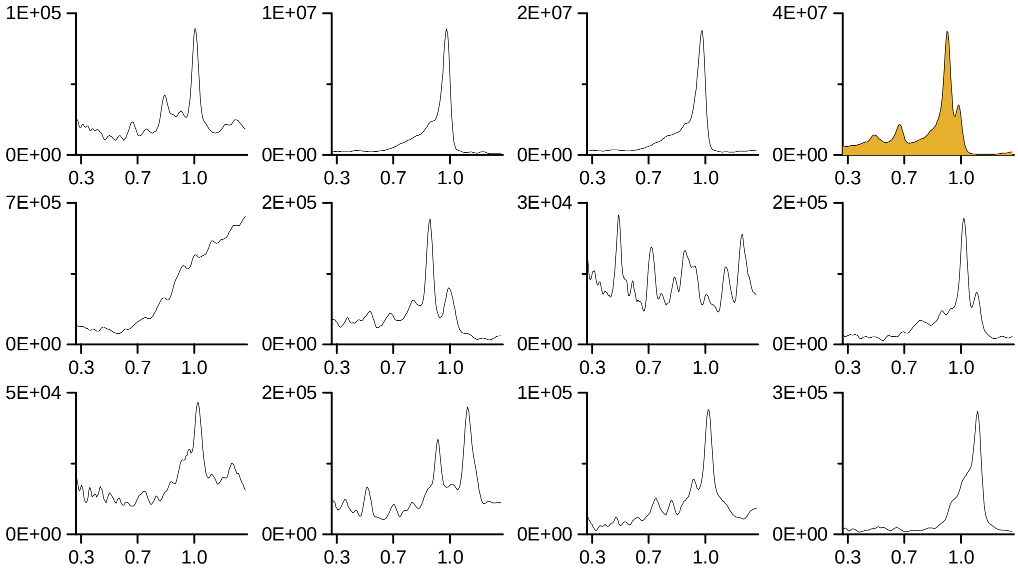

Figure S27. Fingerprints of all studied species with the species-specific method I for *Fraxinus excelsior* (highlighted in color). The y-axes are scaled to the most intensive peak of each sample.

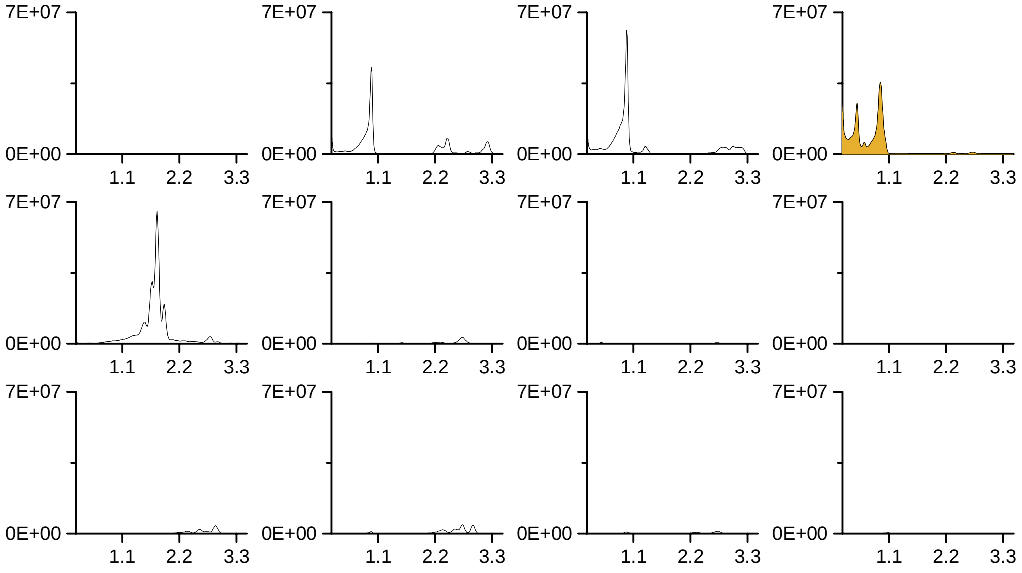

Figure S28. Fingerprints of all studied species with the species-specific method II for *Fraxinus excelsior* (highlighted in color). The y-axes are scaled to the most intensive peak of all samples.

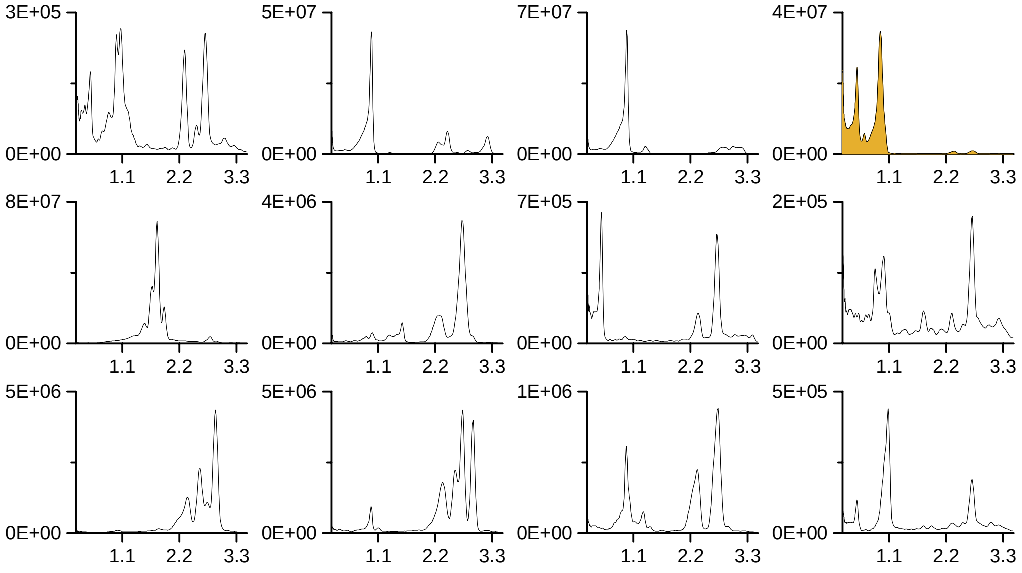

Figure S29. Fingerprints of all studied species with the species-specific method II for *Fraxinus excelsior* (highlighted in color). The y-axes are scaled to the most intensive peak of each sample.

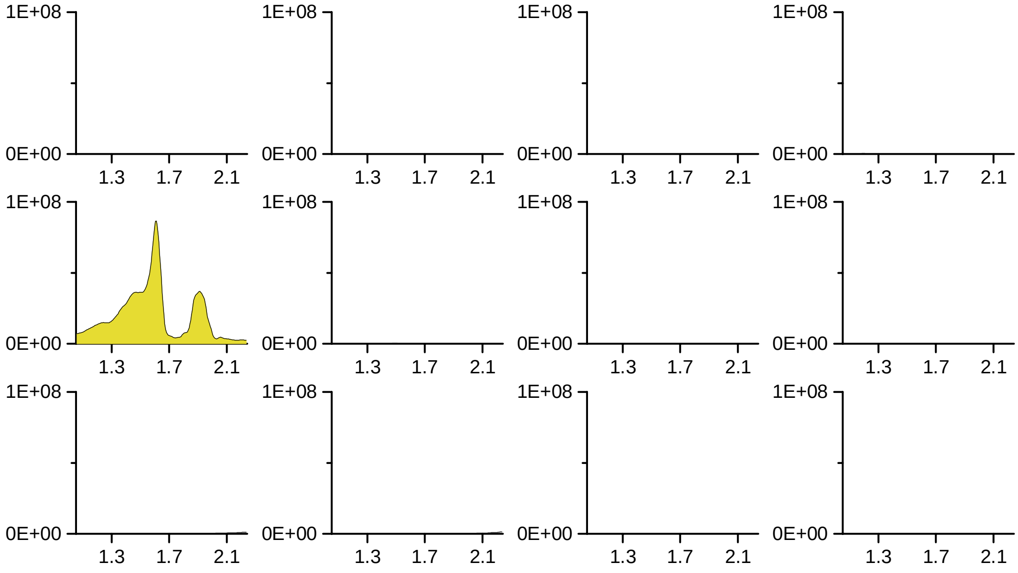

Figure S30. Fingerprints of all studied species with the species-specific method I for *Populus tremula* (highlighted in color). The y-axes are scaled to the most intensive peak of all samples.

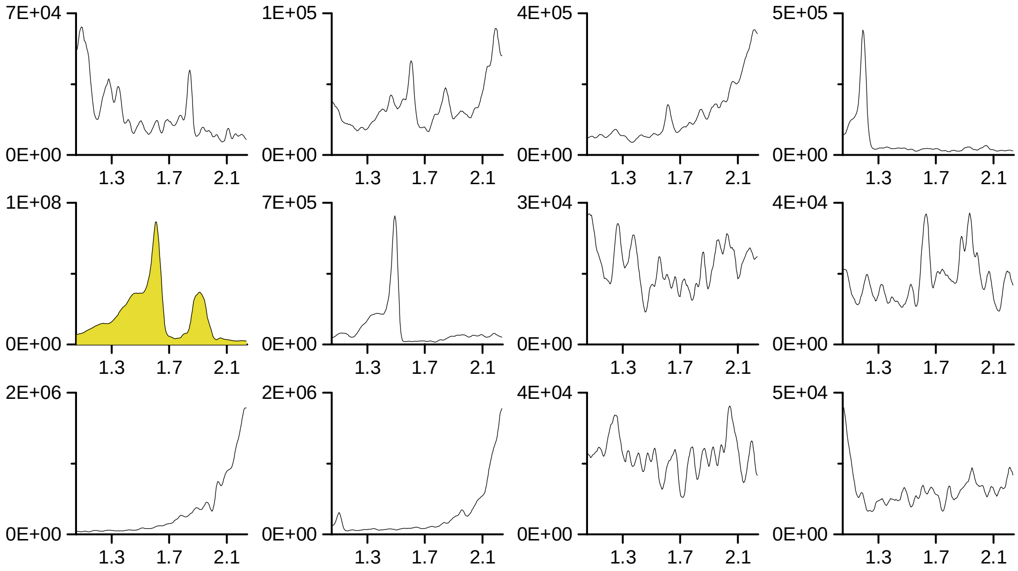

Figure S31. Fingerprints of all studied species with the species-specific method I for *Populus tremula* (highlighted in color). The y-axes are scaled to the most intensive peak of each sample.

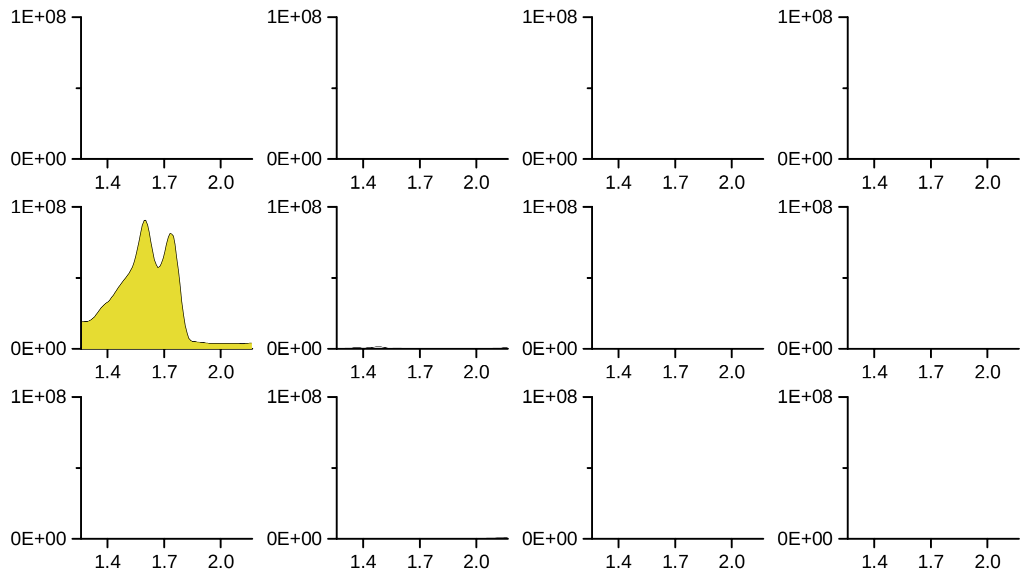

Figure S32. Fingerprints of all studied species with the species-specific method II for *Populus tremula* (highlighted in color). The y-axes are scaled to the most intensive peak of all samples.

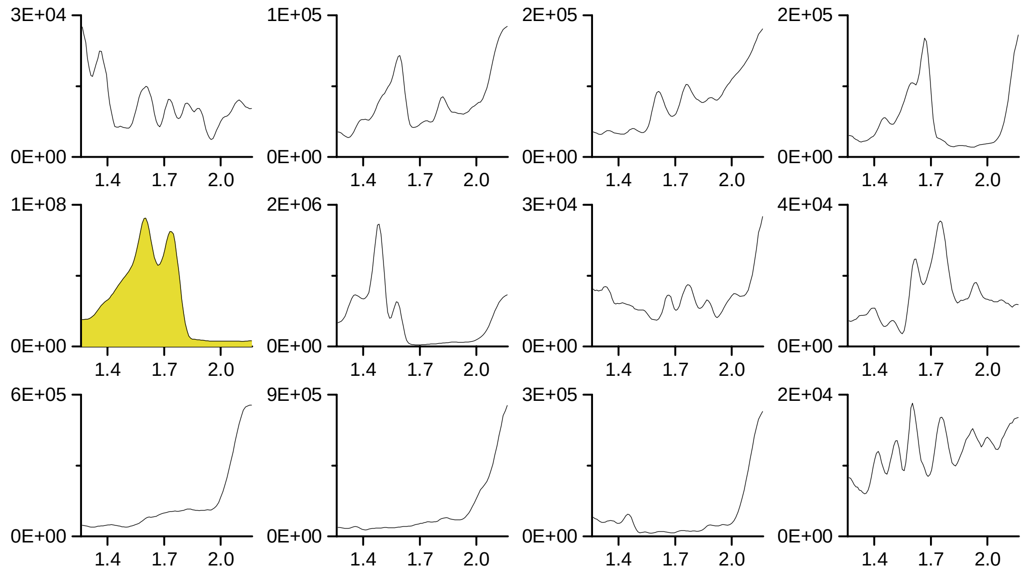

Figure S33. Fingerprints of all studied species with the species-specific method II for *Populus tremula* (highlighted in color). The y-axes are scaled to the most intensive peak of each sample.

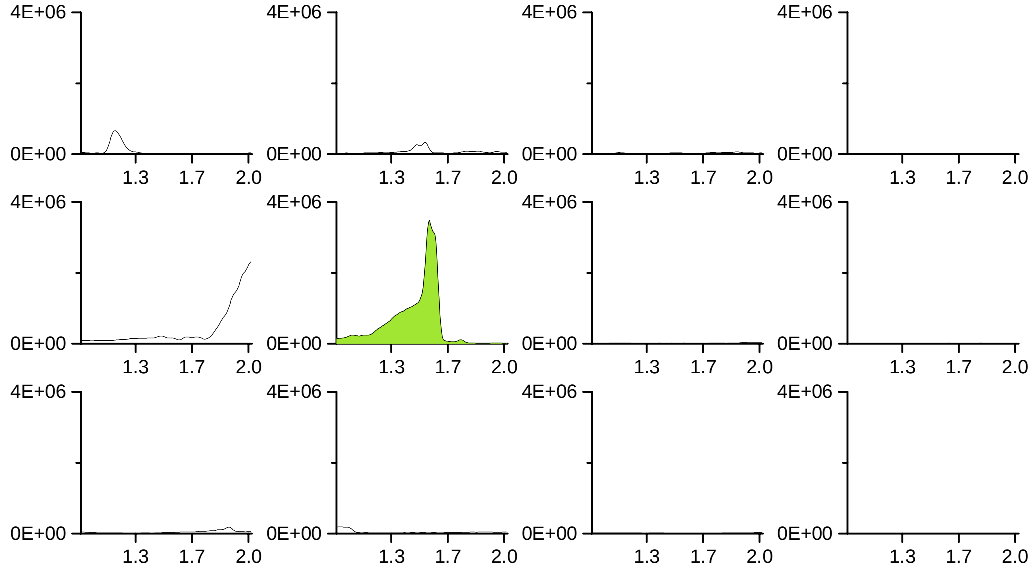

Figure S34. Fingerprints of all studied species with the species-specific method I for *Prunus padus* (highlighted in color). The y-axes are scaled to the most intensive peak of all samples.

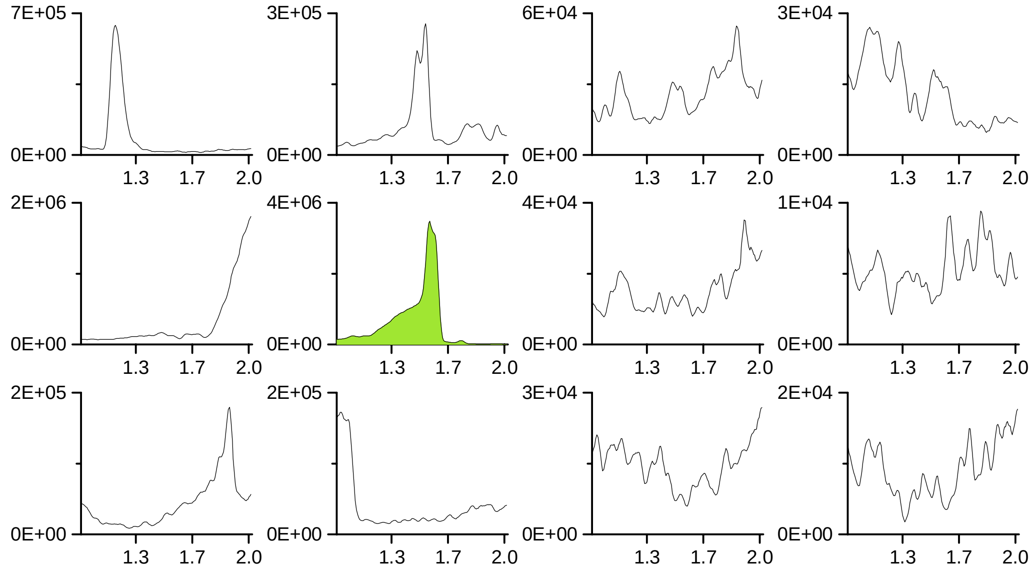

Figure S35. Fingerprints of all studied species with the species-specific method I for *Prunus padus* (highlighted in color). The y-axes are scaled to the most intensive peak of each sample.

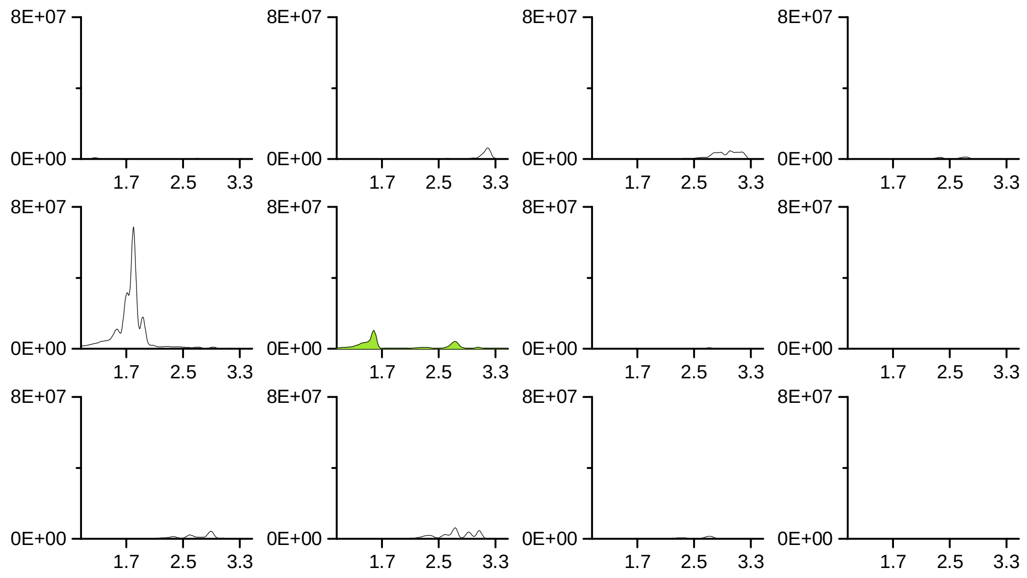

Figure S36. Fingerprints of all studied species with the species-specific method II for *Prunus padus* (highlighted in color). The y-axes are scaled to the most intensive peak of all samples.

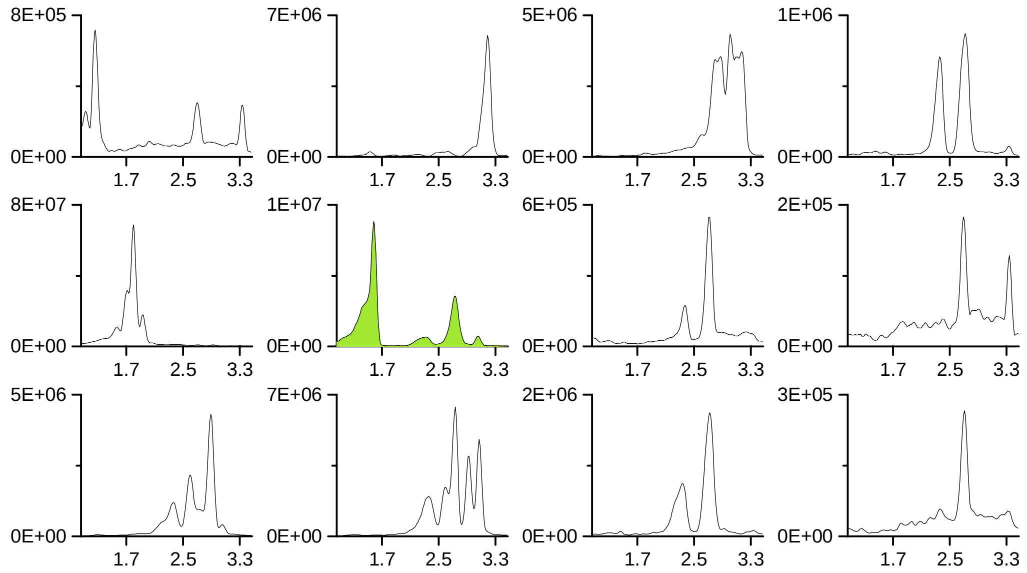

Figure S37. Fingerprints of all studied species with the species-specific method II for *Prunus padus* (highlighted in color). The y-axes are scaled to the most intensive peak of each sample.

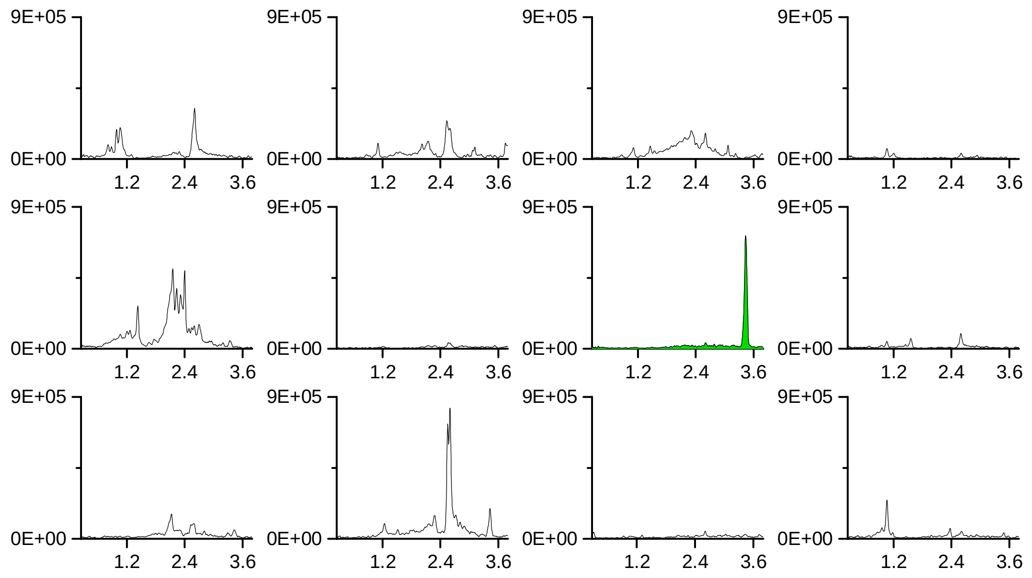

Figure S38. Fingerprints of all studied species with the species-specific method I for *Quercus robur* (highlighted in color). The y-axes are scaled to the most intensive peak of all samples.

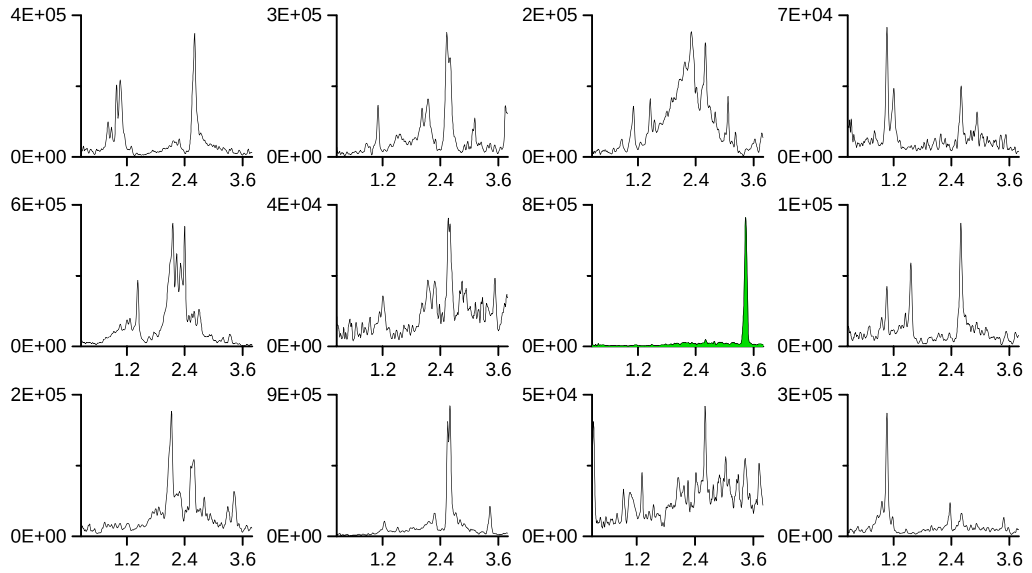

Figure S39. Fingerprints of all studied species with the species-specific method I for *Quercus robur* (highlighted in color). The y-axes are scaled to the most intensive peak of each sample.

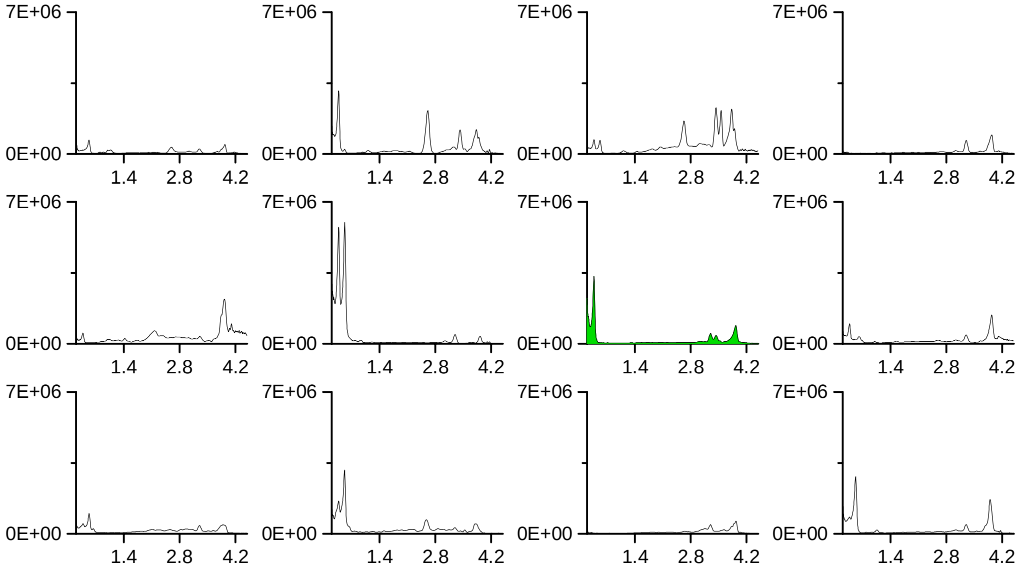

Figure S40. Fingerprints of all studied species with the species-specific method II for *Quercus robur* (highlighted in color). The y-axes are scaled to the most intensive peak of all samples.

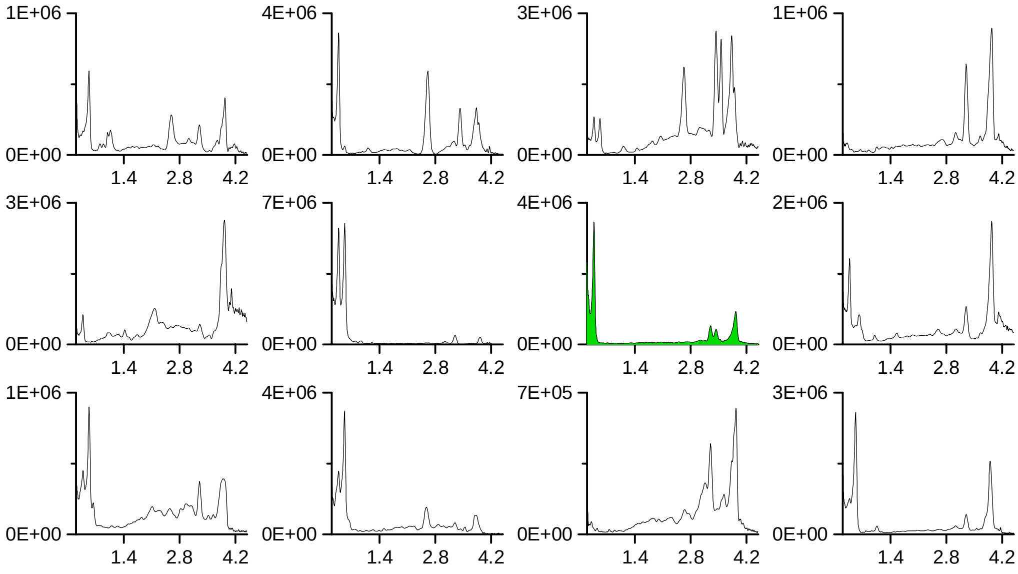

Figure S41. Fingerprints of all studied species with the species-specific method II for *Quercus robur* (highlighted in color). The y-axes are scaled to the most intensive peak of each sample.

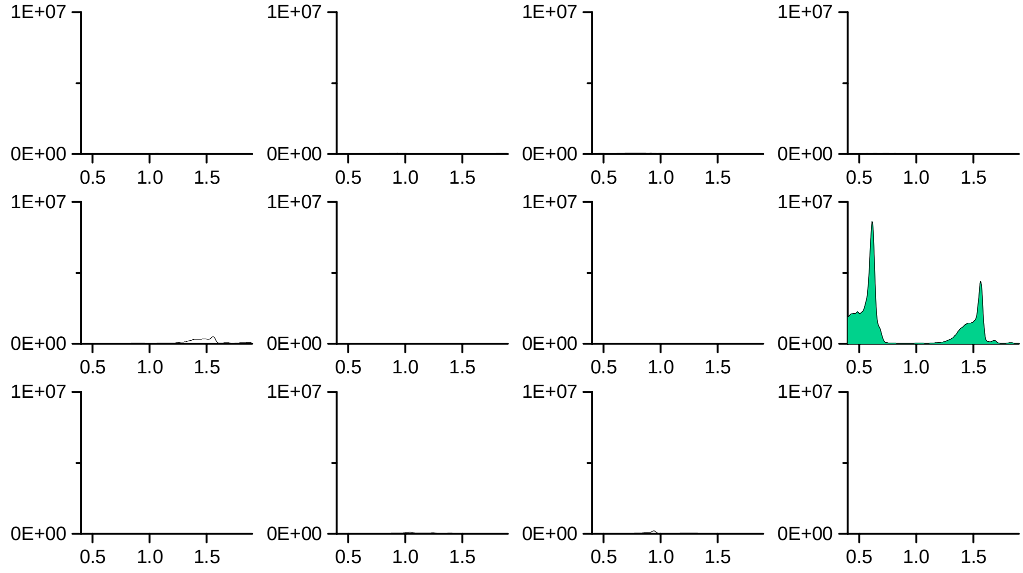

Figure S42. Fingerprints of all studied species with the species-specific method I for *Salix phylicifolia* (highlighted in color). The y-axes are scaled to the most intensive peak of all samples.

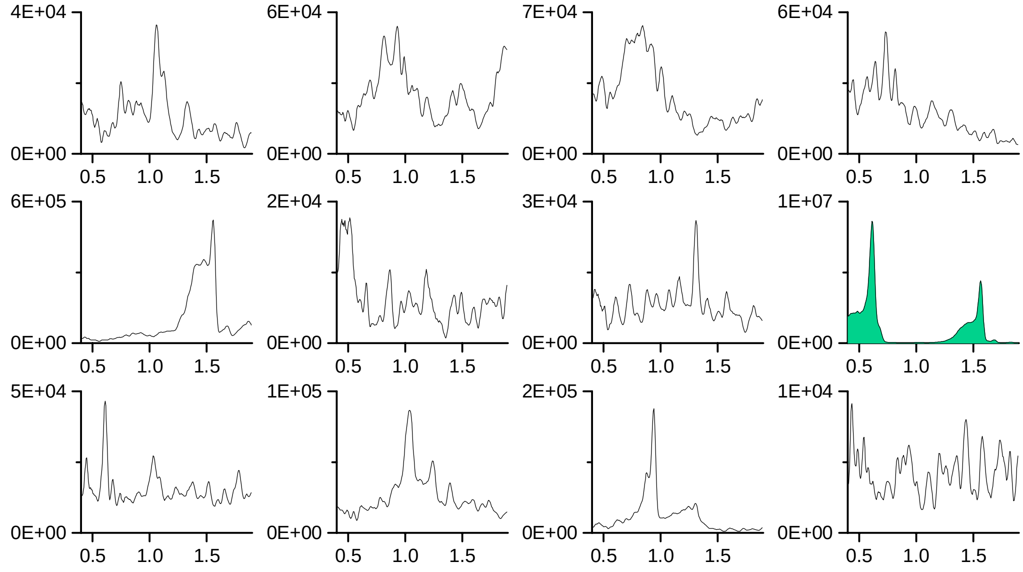

Figure S43. Fingerprints of all studied species with the species-specific method I for *Salix phylicifolia* (highlighted in color). The y-axes are scaled to the most intensive peak of each sample.

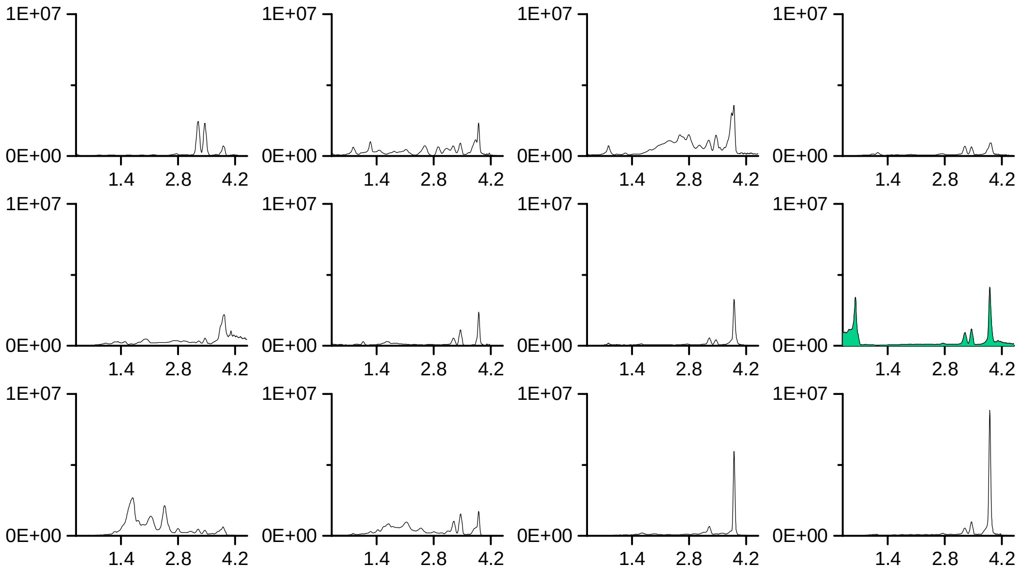

Figure S44. Fingerprints of all studied species with the species-specific method II for *Salix phylicifolia* (highlighted in color). The y-axes are scaled to the most intensive peak of all samples.

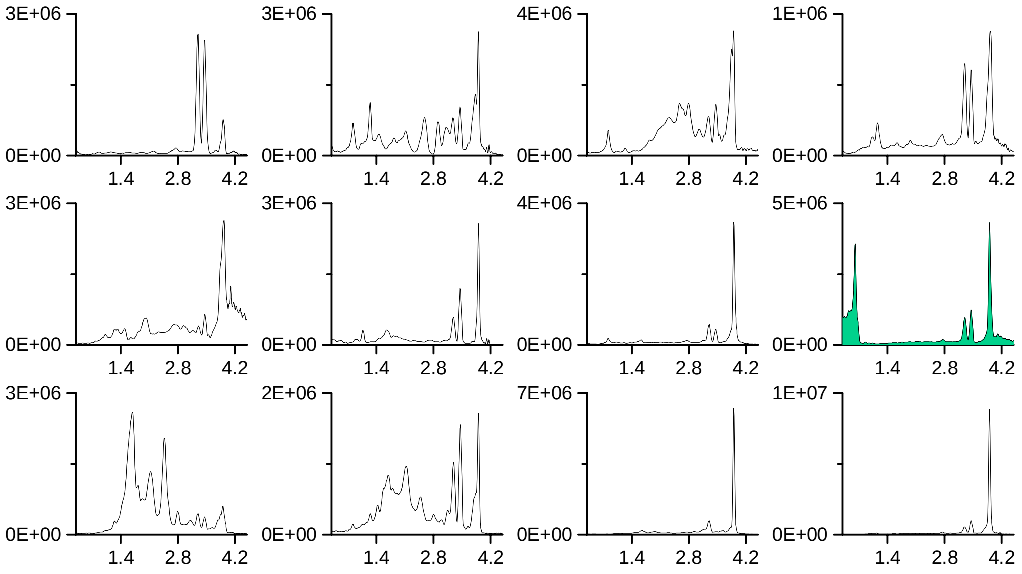

Figure S45. Fingerprints of all studied species with the species-specific method II for *Salix phylicifolia* (highlighted in color). The y-axes are scaled to the most intensive peak of each sample.

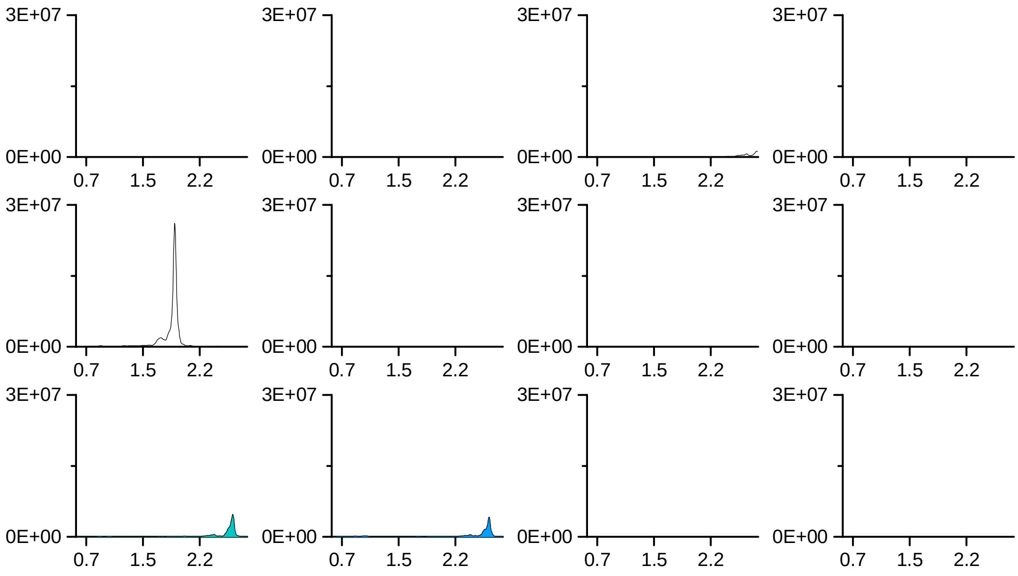

Figure S46. Fingerprints of all studied species with the method I specific to *Sorbus aucuparia* and *Sorbus hybrida* (highlighted in color). The y-axes are scaled to the most intensive peak of all samples.

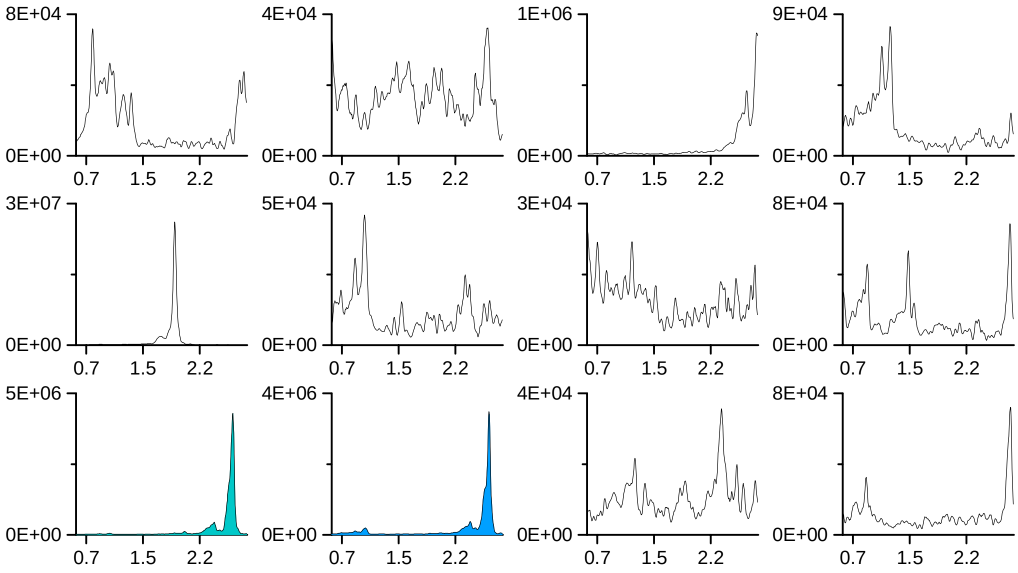

Figure S47. Fingerprints of all studied species with the method I specific to *Sorbus aucuparia* and *Sorbus hybrida* (highlighted in color). The y-axes are scaled to the most intensive peak of each sample.

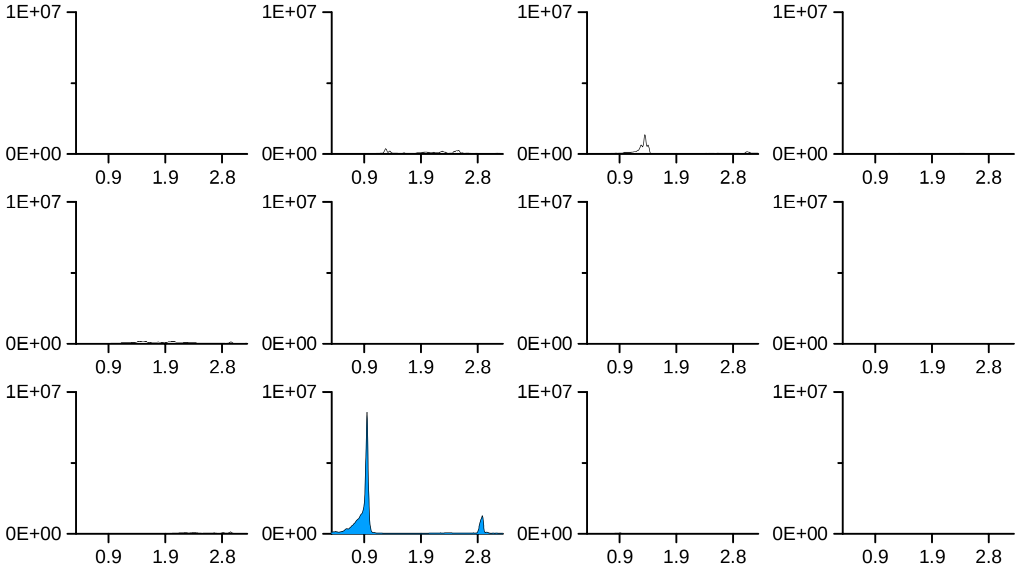

Figure S48. Fingerprints of all studied species with the species-specific method I for *Sorbus hybrida* (highlighted in color). The y-axes are scaled to the most intensive peak of all samples.

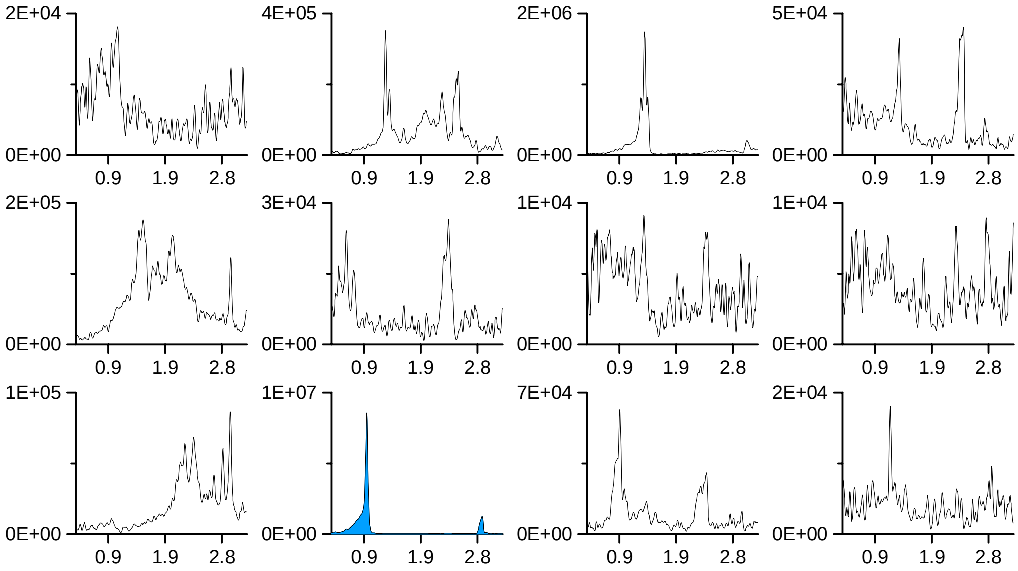

Figure S49. Fingerprints of all studied species with the species-specific method I for *Sorbus hybrida* (highlighted in color). The y-axes are scaled to the most intensive peak of each sample.

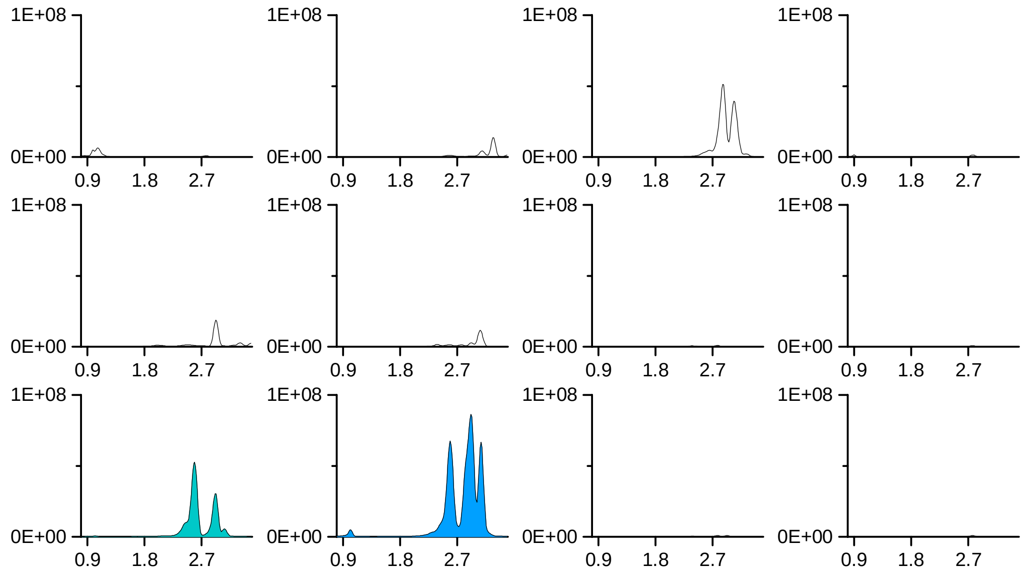

Figure S50. Fingerprints of all studied species with the method II specific to *Sorbus aucuparia* and *Sorbus hybrida* (highlighted in color). The y-axes are scaled to the most intensive peak of all samples.

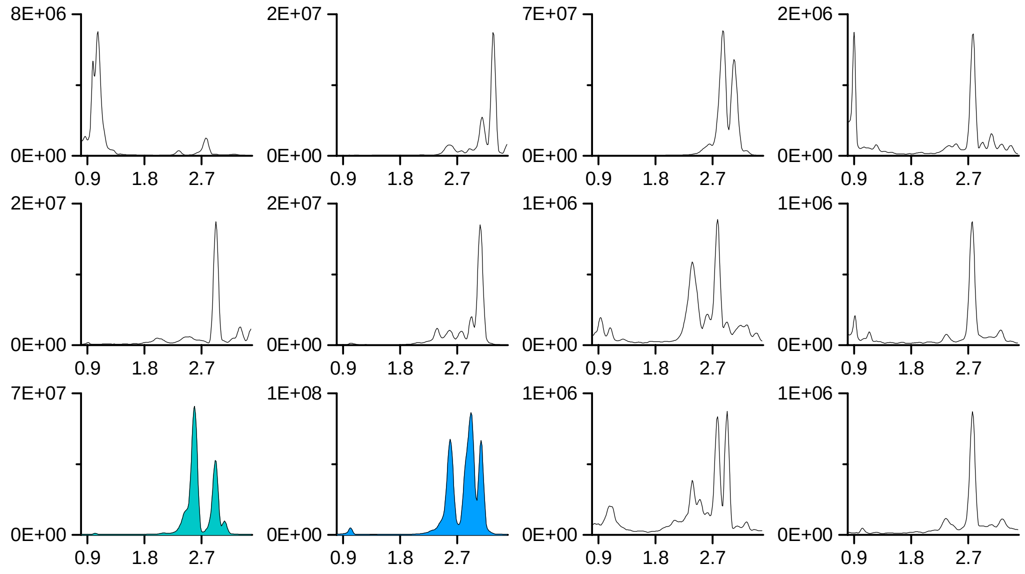

Figure S51. Fingerprints of all studied species with the method II specific to *Sorbus aucuparia* and *Sorbus hybrida* (highlighted in color). The y-axes are scaled to the most intensive peak of each sample.

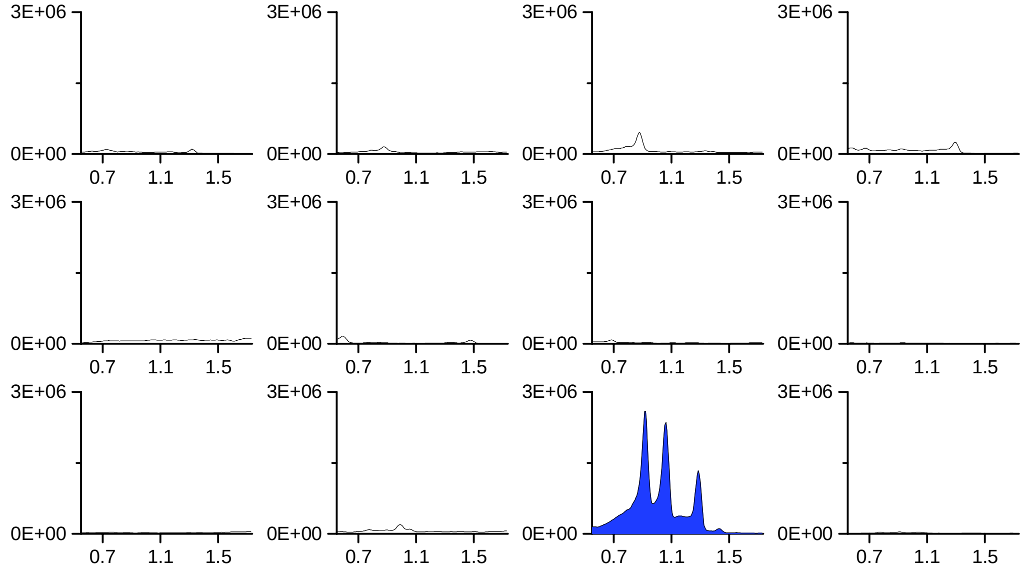

Figure S52. Fingerprints of all studied species with the species-specific method I for *Syringsa vulgaris* (highlighted in color). The y-axes are scaled to the most intensive peak of all samples.

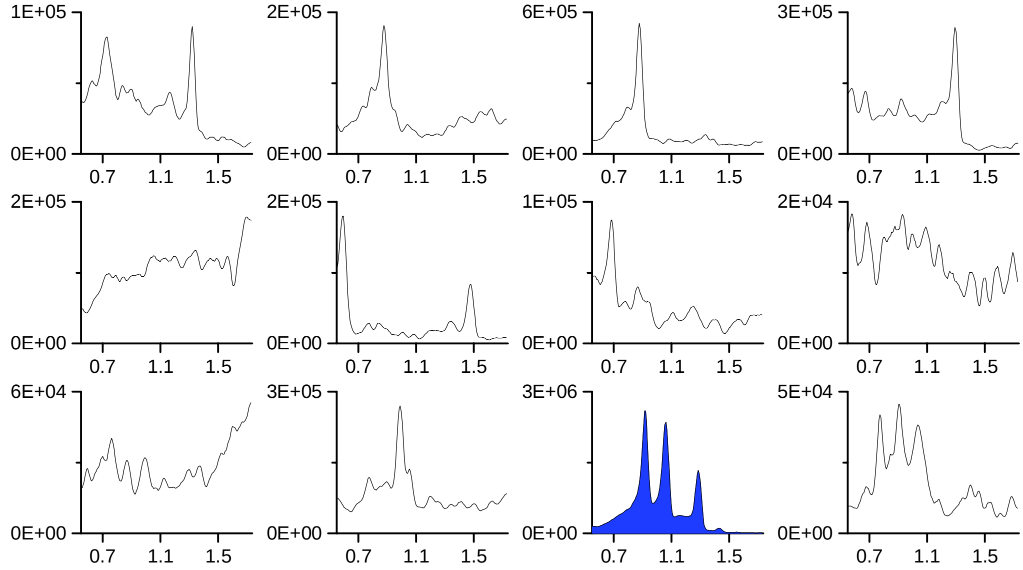

Figure S53. Fingerprints of all studied species with the species-specific method I for *Syringsa vulgaris* (highlighted in color). The y-axes are scaled to the most intensive peak of each sample.

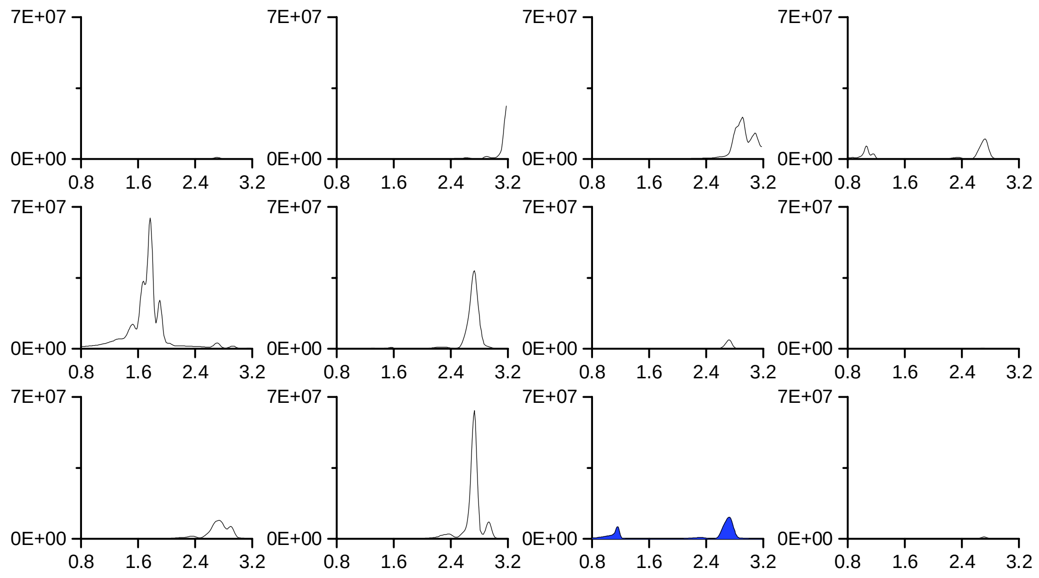

Figure S54. Fingerprints of all studied species with the species-specific method II for *Syringsa vulgaris* (highlighted in color). The y-axes are scaled to the most intensive peak of all samples.

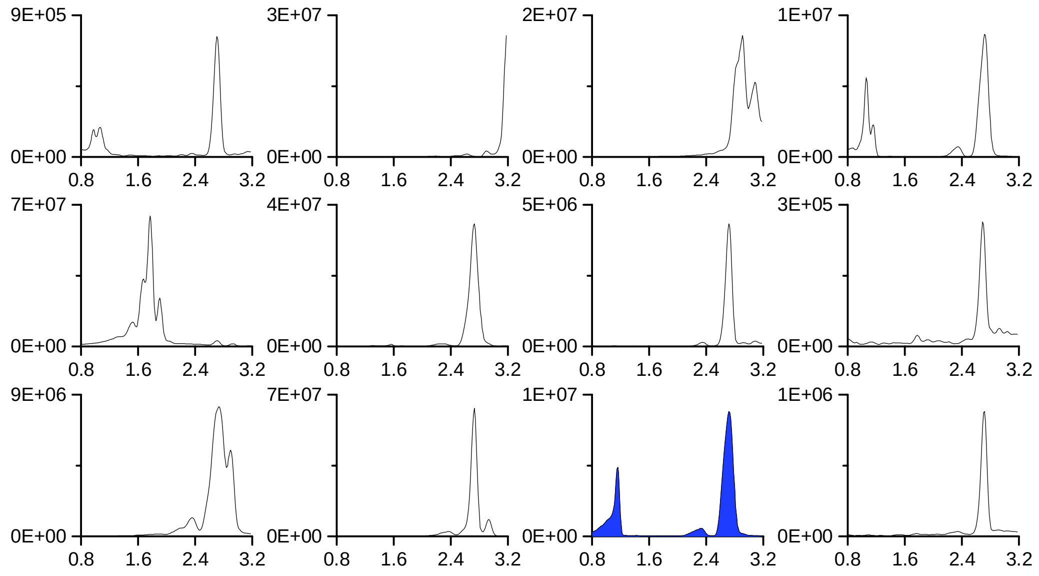

Figure S55. Fingerprints of all studied species with the species-specific method II for *Syringsa vulgaris* (highlighted in color). The y-axes are scaled to the most intensive peak of each sample.

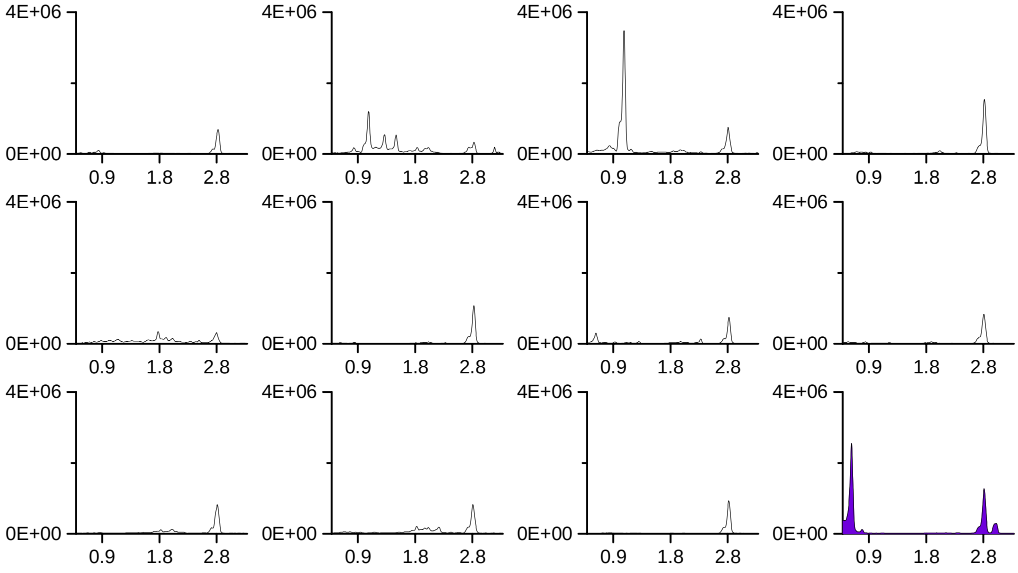

Figure S56. Fingerprints of all studied species with the method I specific to *Tilia cordata* and *Tilia* × *europaea* (highlighted in color). The fingerprints of both *Tilia* species were similar. The y-axes are scaled to the most intensive peak of all samples.

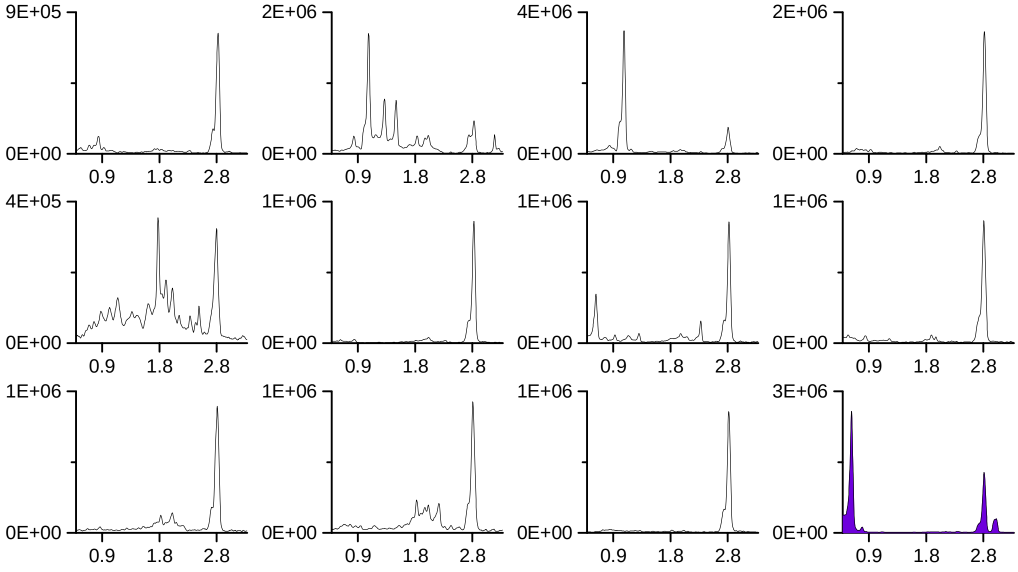

Figure S57. Fingerprints of all studied species with the method I specific to *Tilia cordata* and *Tilia* × *europaea* (highlighted in color). The fingerprints of both *Tilia* species were similar. The y-axes are scaled to the most intensive peak of each sample.

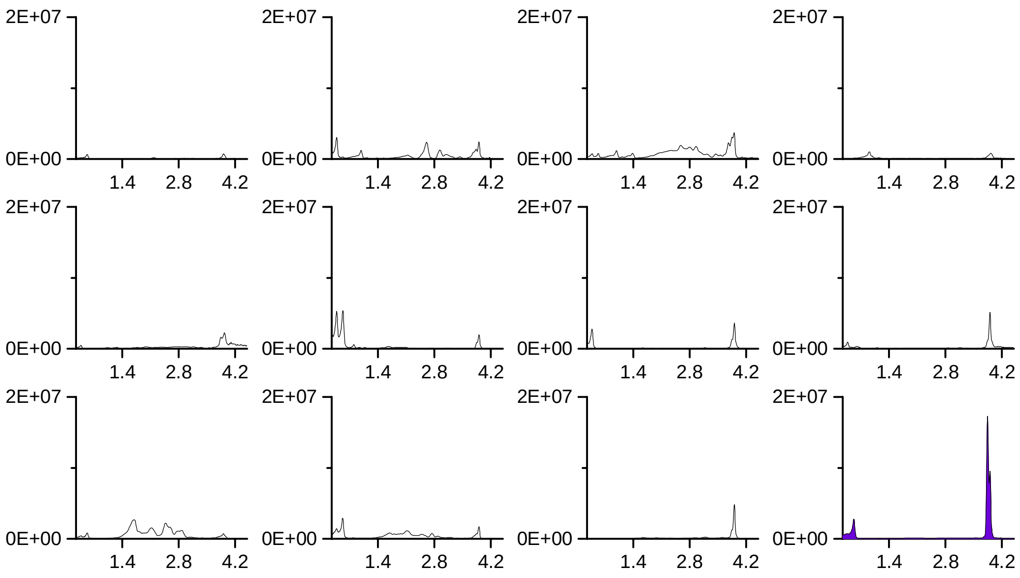

Figure S58. Fingerprints of all studied species with the method II specific to *Tilia cordata* and *Tilia × europaea* (highlighted in color). The fingerprints of both *Tilia* species were similar. The y-axes are scaled to the most intensive peak of all samples.

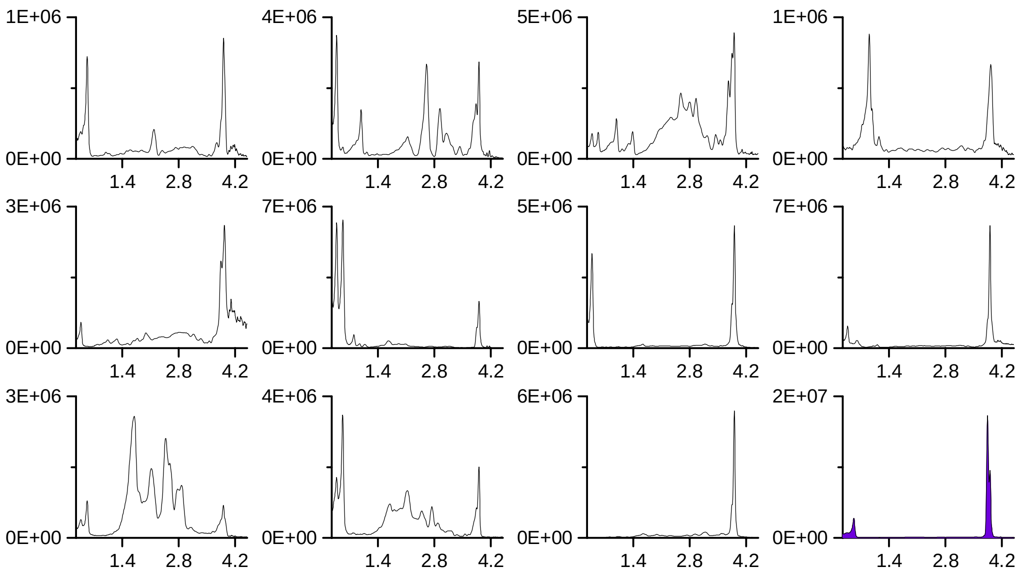

Figure S59. Fingerprints of all studied species with the method II specific to *Tilia cordata* and *Tilia × europaea* (highlighted in color). The fingerprints of both *Tilia* species were similar. The y-axes are scaled to the most intensive peak of each sample.

Section 3. MS/MS spectra, fragmentation patterns, *m/z* values and other mass spectrometric information of the fragments of the markers (**1-42**) used in the final fingerprinting methods

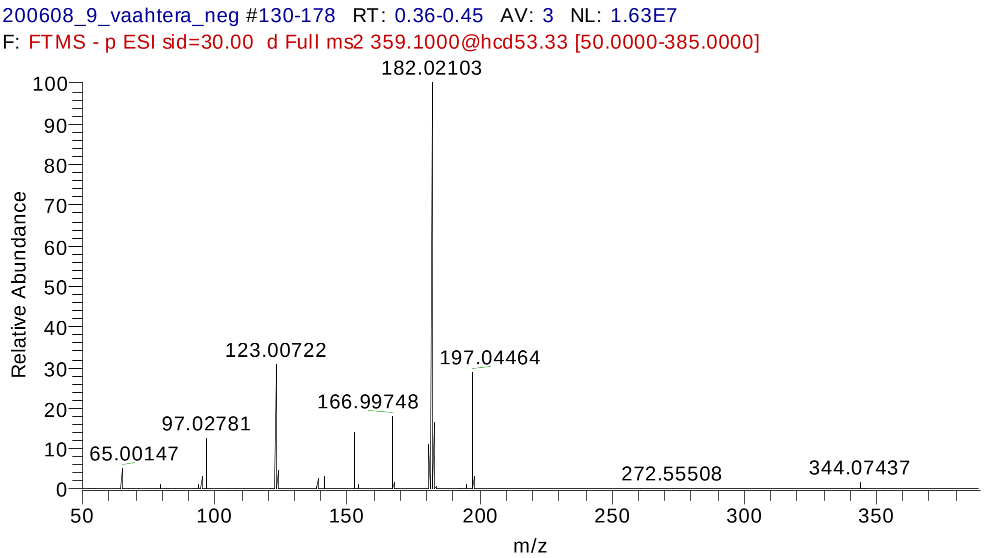

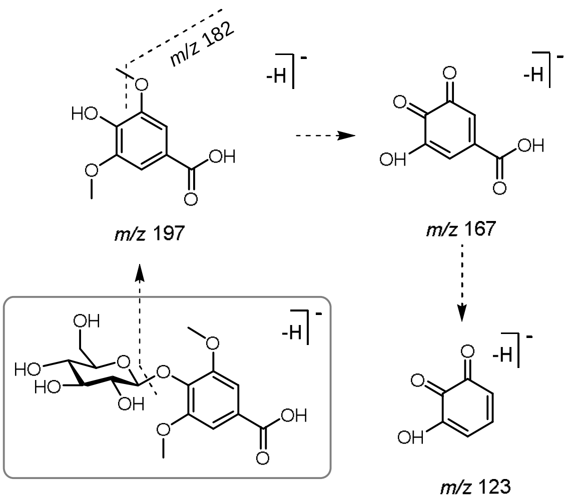

Figure S60. MS/MS spectrum and proposed fragmentation pattern of compound **1**.

Table S1. *m/z* values, molecular formulae, rings and double bond equivalents (RDB) and mass errors for the fragments of compound **1**.

m/z value	Molecular formula	RDB	Error (ppm)
123.00722	$C_6H_4O_3$	5	-12.579
166.99748	$C_7H_4O_5$	6	-6.686
182.02103	$C_8H_6O_5$	6	-5.722
197.04464	$C_9H_{10}O_5$	5	-4.601

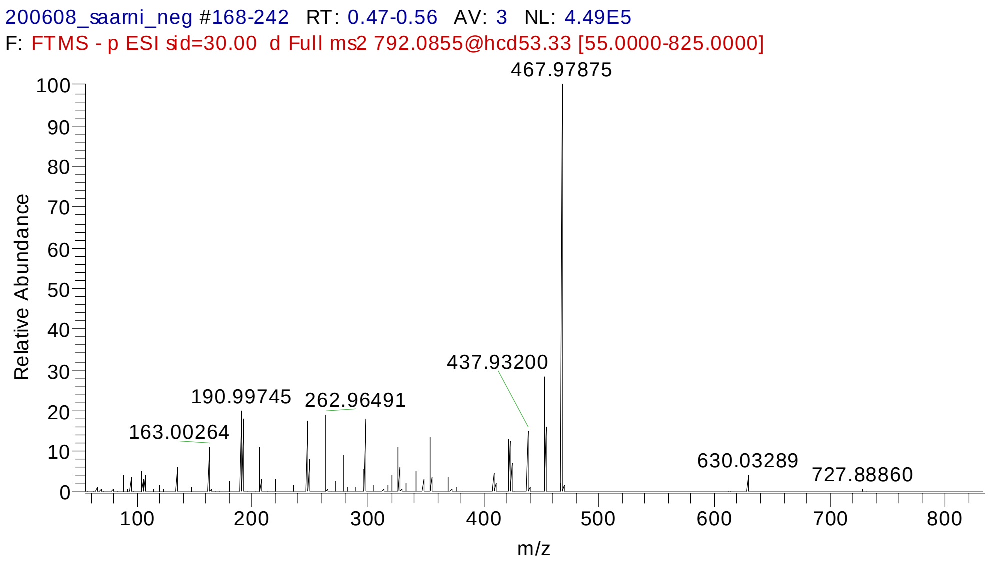

Figure S61. MS/MS spectrum of compound **2**.

Table S2. *m/z* values, molecular formulae, rings and double bond equivalents (RDB) and mass errors for the fragments of compound **2**. n/a = not available.

m/z value	Molecular formula	RDB	Error (ppm)
190.99745	n/a	n/a	n/a
247.94137	n/a	n/a	n/a
262.96491	n/a	n/a	n/a
297.95710	n/a	n/a	n/a
353.94649	n/a	n/a	n/a
421.99466	n/a	n/a	n/a
437.93200	n/a	n/a	n/a
452.95525	n/a	n/a	n/a
467.97875	n/a	n/a	n/a
630.03289	n/a	n/a	n/a

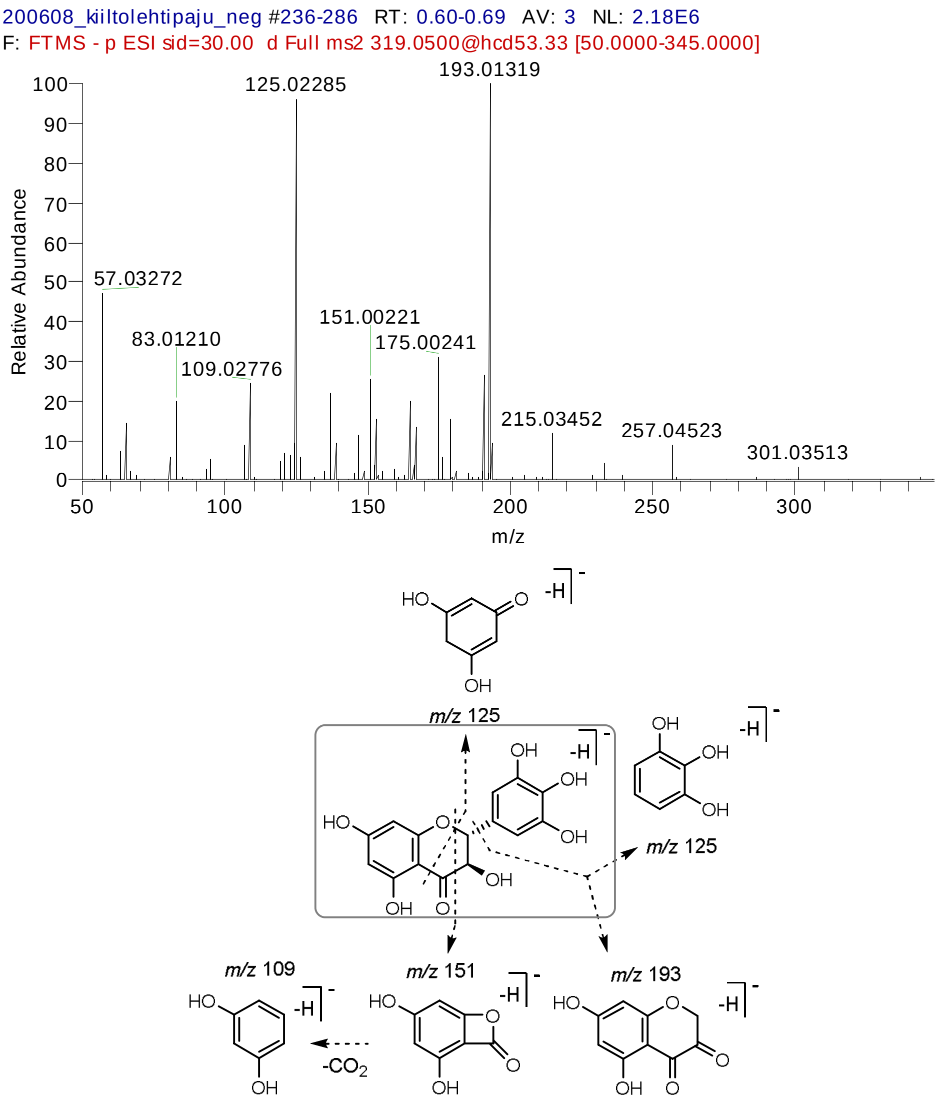

Figure S62. MS/MS spectrum and proposed fragmentation pattern of compound **3.**

Table S3. *m/z* values, molecular formulae, rings and double bond equivalents (RDB) and mass errors for the fragments of compound **3**.

m/z value	Molecular formula	RDB	Error (ppm)
109.02776	$C_6H_6O_2$	4	-15.984
125.02285	$C_6H_6O_3$	4	-12.537
151.00221	$C_7H_4O_4$	6	-9.747
193.01319	$C_9H_6O_5$	7	-5.474

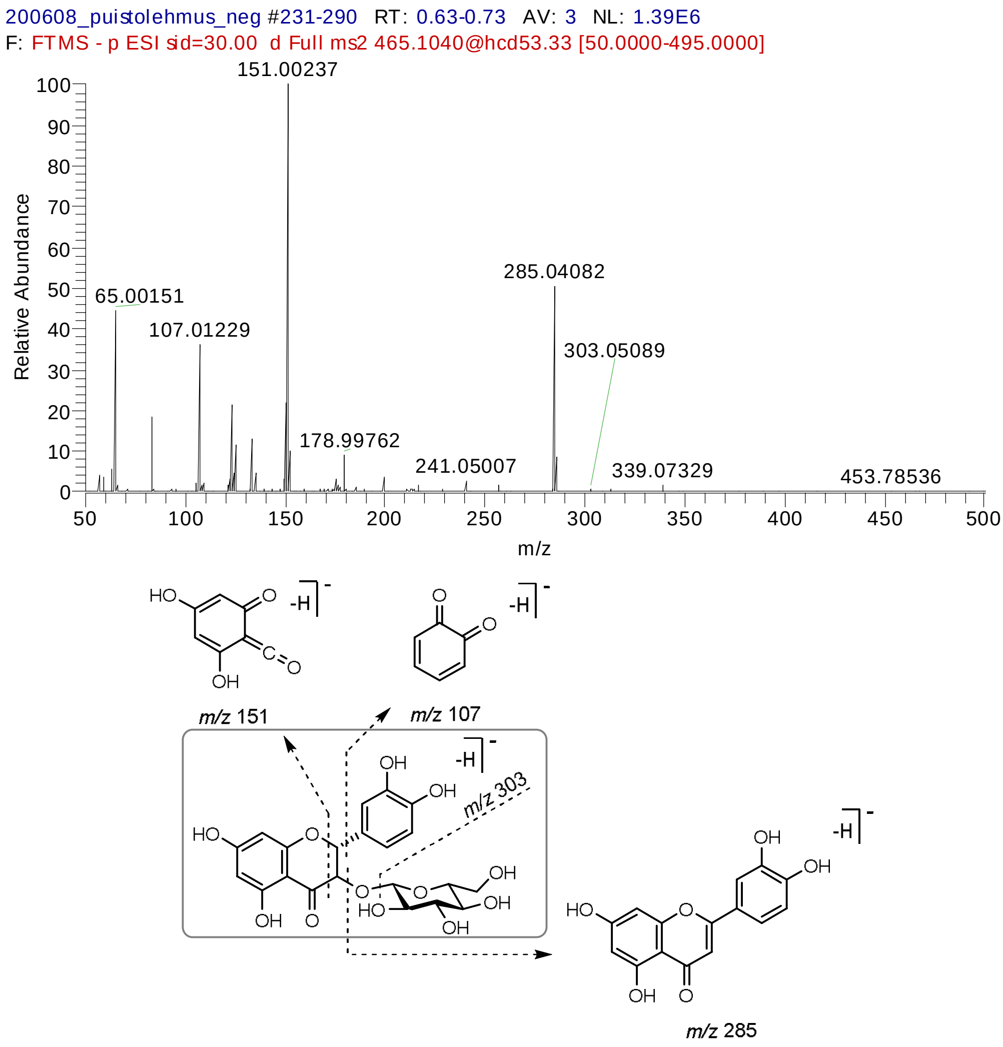

Figure S63. MS/MS spectrum and proposed fragmentation pattern of compound **4**.

Table S4. *m/z* values, molecular formulae, rings and double bond equivalents (RDB) and mass errors for the fragments of compound **4**.

m/z value	Molecular formula	RDB	Error (ppm)
107.01229	$C_6H_4O_2$	5	-14.603
151.00237	$C_7H_4O_4$	6	-8.688
285.04082	$C_{15}H_{10}O_6$	11	1.259
303.05089	$C_{15}H_{12}O_7$	10	-0.449

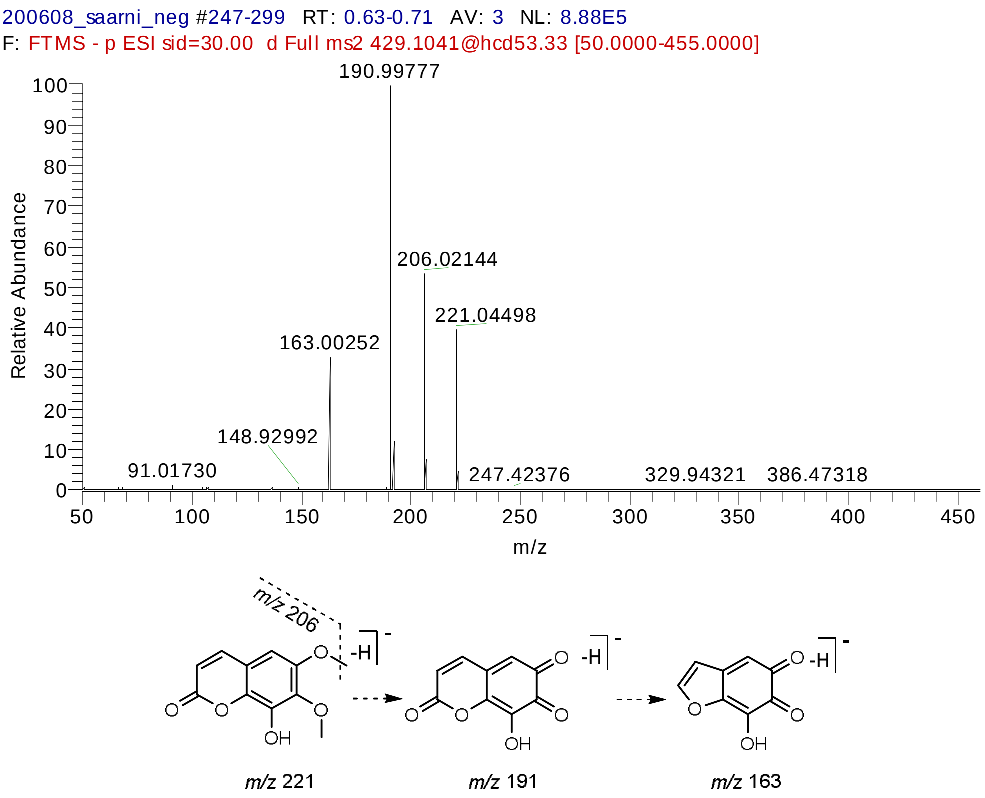

Figure S64. MS/MS spectrum and proposed fragmentation pattern of compound **5**.

Table S5. *m/z* values, molecular formulae, rings and double bond equivalents (RDB) and mass errors for the fragments of compound **5**.

m/z value	Molecular formula	RDB	Error (ppm)
163.00252	$C_8H_4O_4$	7	-7.128
190.99777	$C_9H_4O_5$	8	-4.327
206.02144	$C_{10}H_6O_5$	8	-3.066
221.04498	$C_{11}H_{10}O_5$	7	-2.564

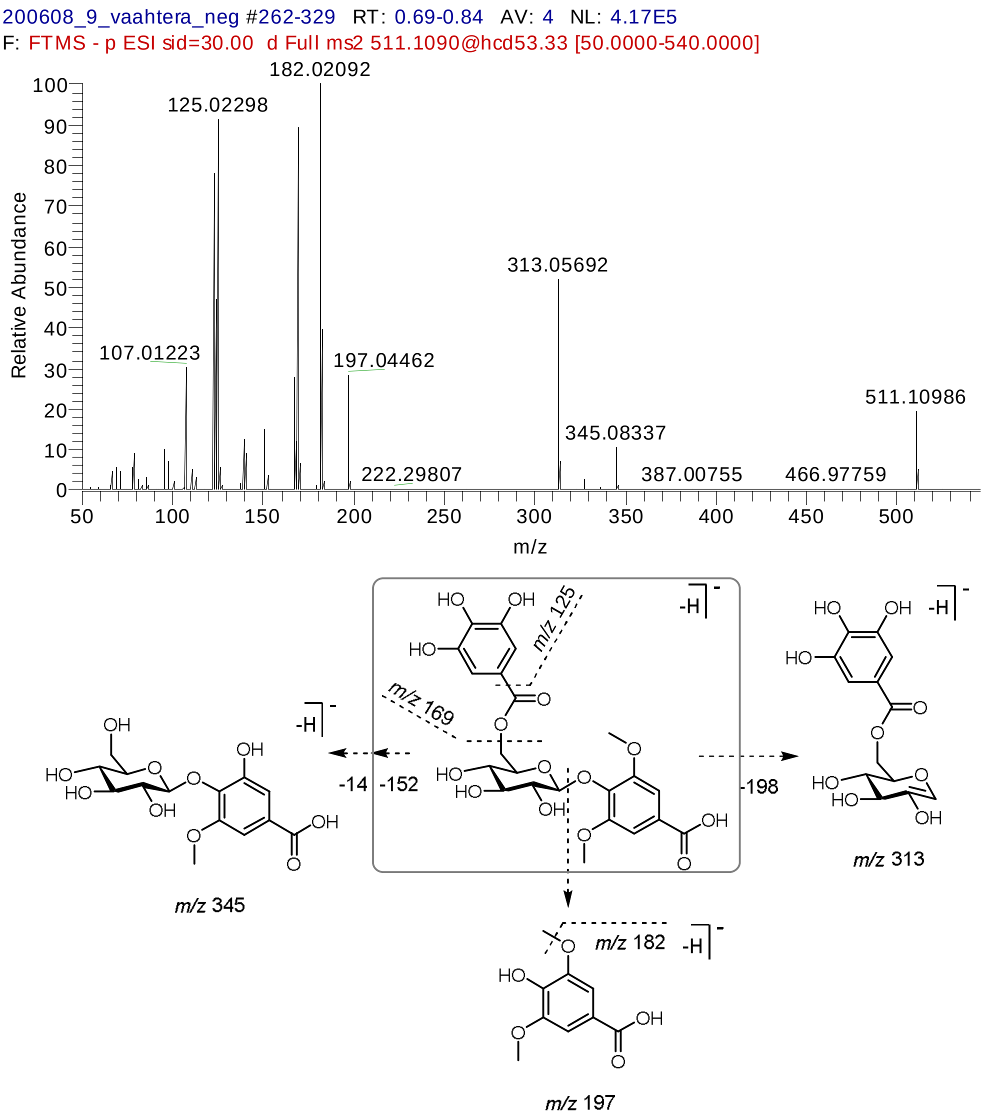

Figure S65. MS/MS spectrum of isomeric compounds **6**, **7** and **8** and the proposed fragmentation pattern of one of the isomers.

Table S6. *m/z* values, molecular formulae, rings and double bond equivalents (RDB) and mass errors for the fragments of compounds **6**, **7** and **8**.

m/z value	Molecular formula	RDB	Error (ppm)
125.02298	$C_6H_6O_3$	4	-11.497
169.01308	$C_7H_6O_5$	5	-6.902
182.02092	$C_8H_6O_5$	6	-6.327
197.04462	$C_9H_{10}O_5$	5	-4.703
313.05692	$C_{13}H_{14}O_9$	7	1.325
345.08337	$C_{14}H_{18}O_{10}$	6	1.884

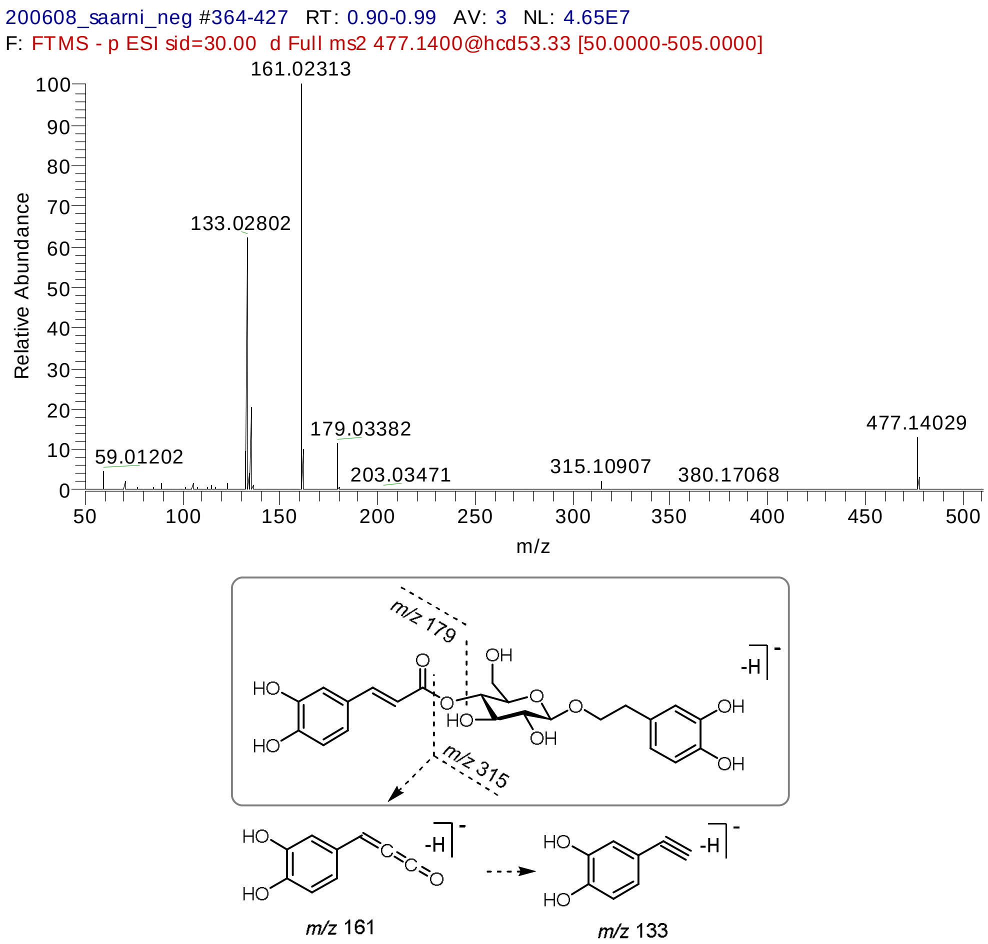

Figure S66. MS/MS spectrum of isomeric compounds **9** and **12** and the proposed fragmentation pattern of one of the isomers.

Table S7. *m/z* values, molecular formulae, rings and double bond equivalents (RDB) and mass errors for the fragments of compounds **9** and **12**.

m/z value	Molecular formula	RDB	Error (ppm)
133.02802	$C_8H_6O_2$	6	-11.146
161.02313	$C_9H_6O_3$	7	-7.995
179.03382	$C_9H_8O_4$	6	-6.490
315.10907	$C_{14}H_{20}O_8$	5	1.680

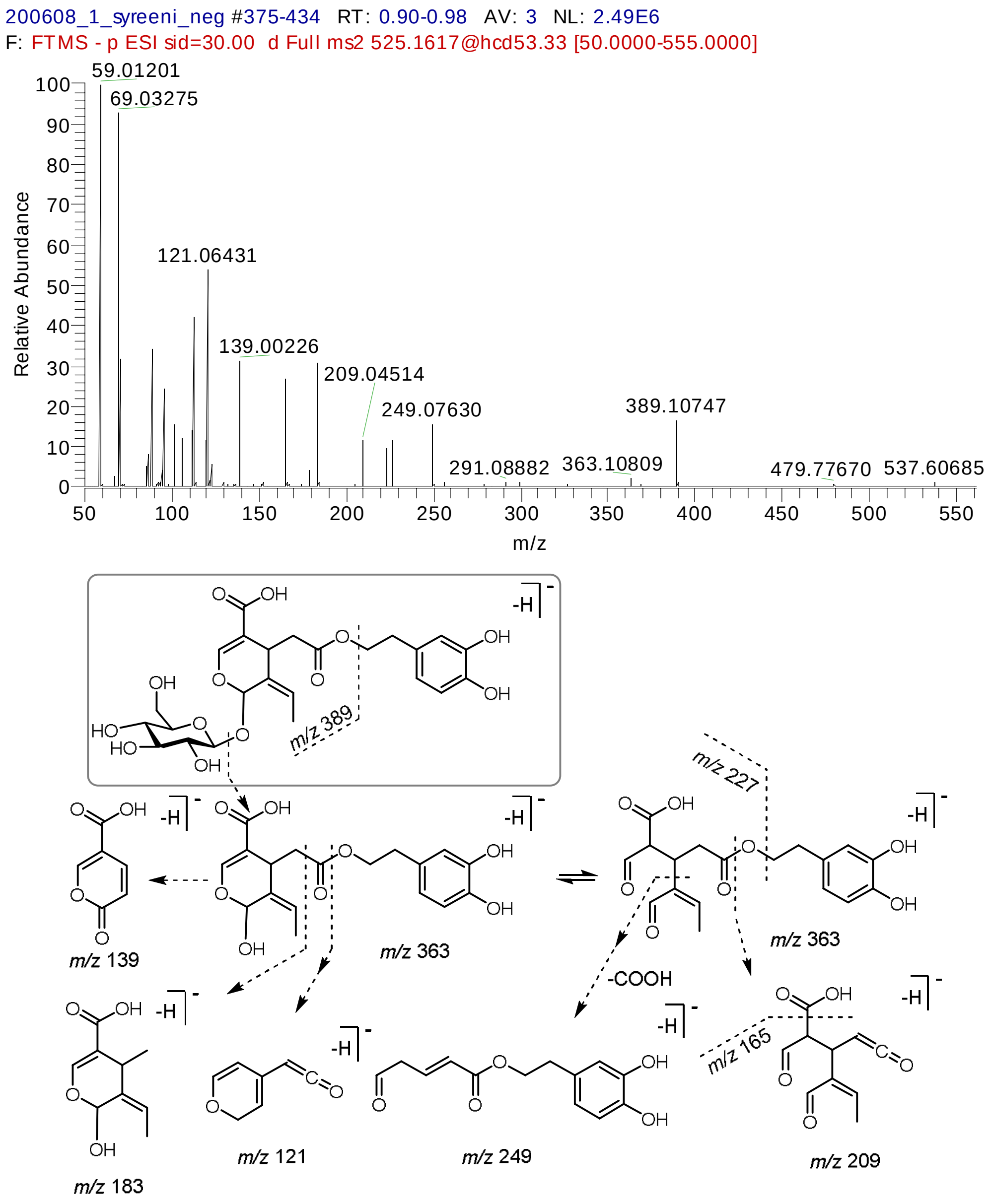

Figure S67. MS/MS spectrum and proposed fragmentation pattern of compound **10**.

Table S8. *m/z* values, molecular formulae, rings and double bond equivalents (RDB) and mass errors for the fragments of compound **10**.

m/z value	Molecular formula	RDB	Error (ppm)
121.02783	$C_7H_6O_2$	5	-13.821
139.00226	$C_6H_4O_4$	5	-10.229
165.05446	$C_9H_9O_3$	5	-7.619
183.06536	$C_9H_{12}O_4$	4	-5.037
209.04514	$C_{10}H_{10}O_5$	6	-1.945
227.05561	$C_{10}H_{12}O_6$	5	-2.208
249.07630	$C_{13}H_{14}O_5$	7	-2.195
363.10809	$C_{18}H_{20}O_8$	9	-1.241
389.10747	$C_{16}H_{22}O_{11}$	6	-3.764

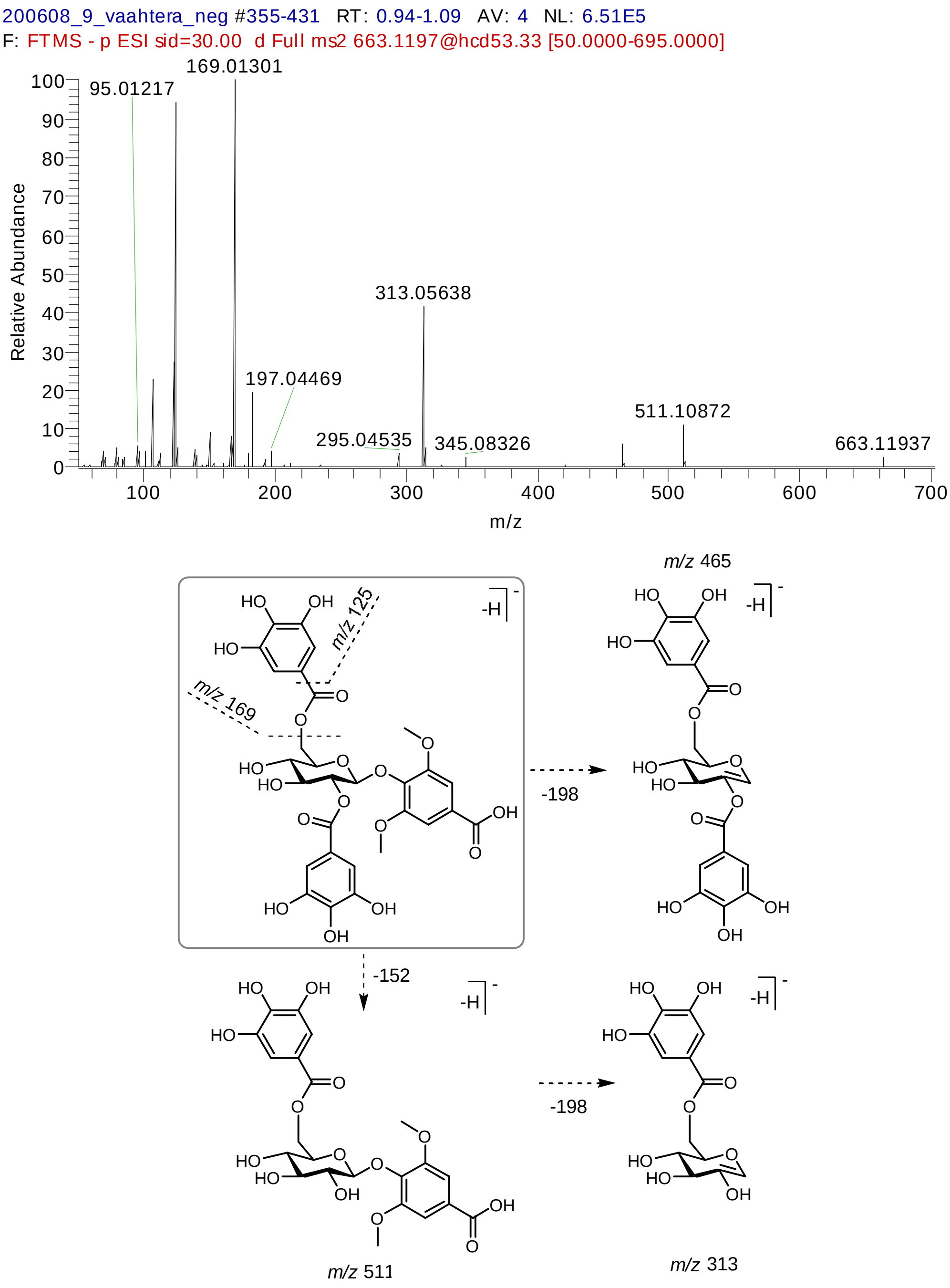

Figure S68. MS/MS spectrum and proposed fragmentation pattern of compound **11**.

Table S9. *m/z* values, molecular formulae, rings and double bond equivalents (RDB) and mass errors for the fragments of compound **11**.

m/z value	Molecular formula	RDB	Error (ppm)
125.02290	$C_6H_6O_3$	4	-12.137
169.01310	$C_7H_6O_5$	5	-6.784
313.05693	$C_{13}H_{14}O_9$	7	1.357
465.06689	$C_{20}H_{18}O_{13}$	12	-1.234
511.10934	$C_{22}H_{24}O_{14}$	11	0.023

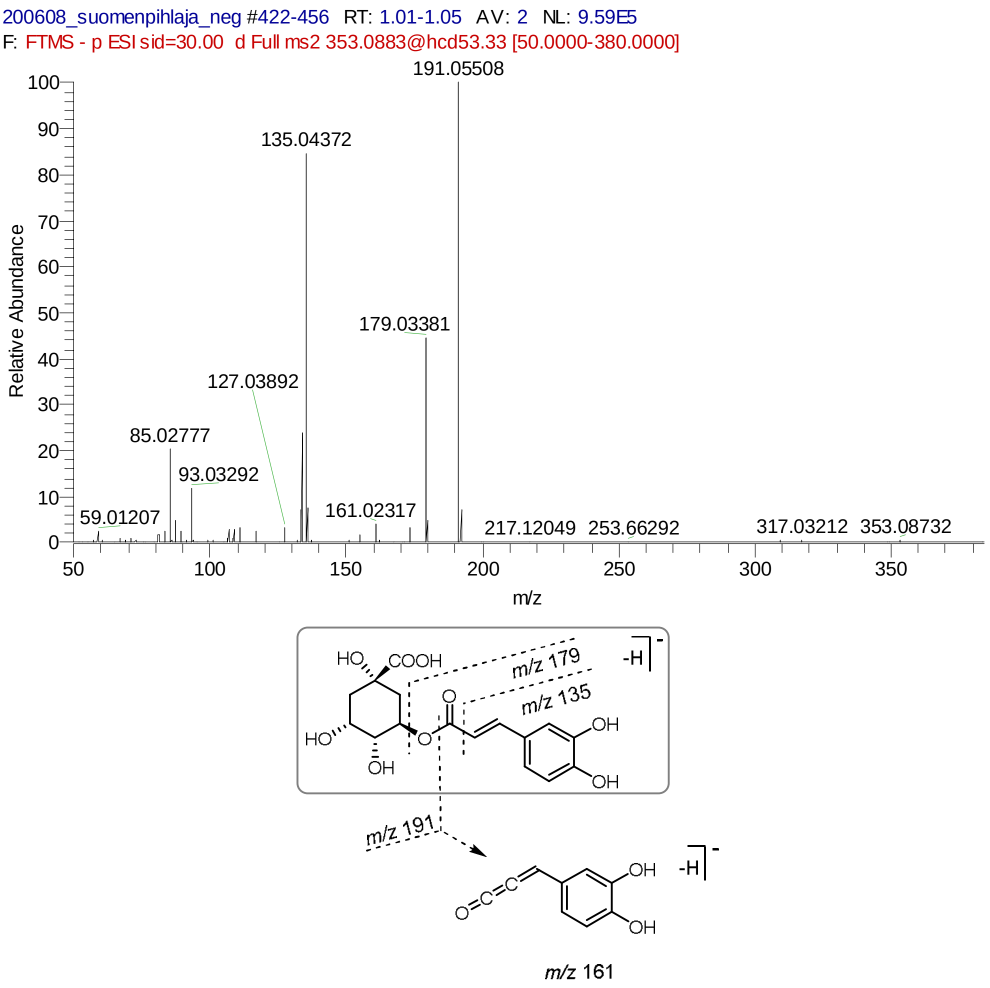

Figure S69. MS/MS spectrum and proposed fragmentation pattern of compound **13**.

Table S10. *m/z* values, molecular formulae, rings and double bond equivalents (RDB) and mass errors for the fragments of compound **13**.

m/z value	Molecular formula	RDB	Error (ppm)
135.04372	$C_8H_8O_2$	5	-10.610
161.02317	$C_9H_6O_3$	7	-7.746
179.03381	$C_9H_8O_4$	6	-6.546
191.05508	$C_7H_{12}O_6$	2	-5.398

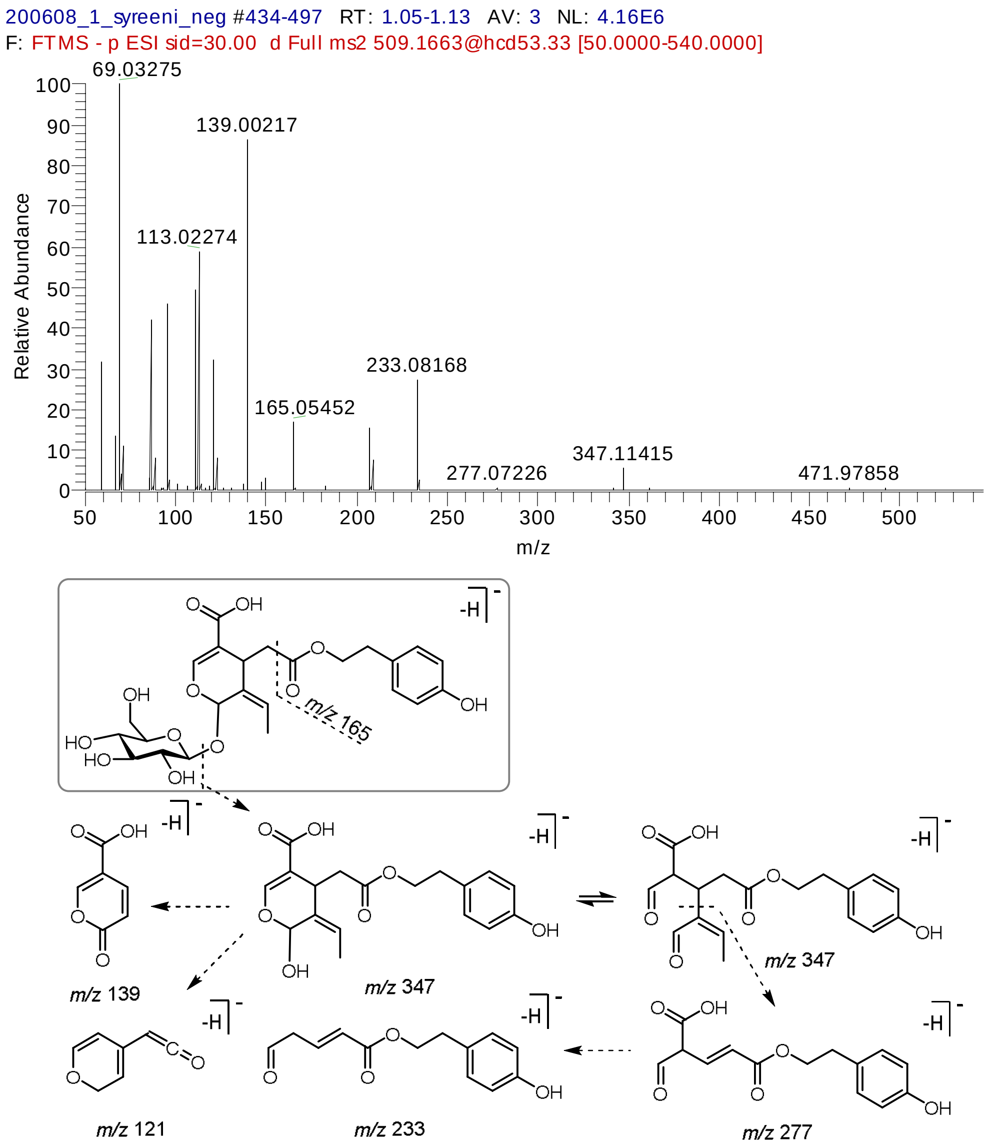

Figure S70. MS/MS spectrum and proposed fragmentation pattern of compound **14**.

Table S11. *m/z* values, molecular formulae, rings and double bond equivalents (RDB) and mass errors for the fragments of compound **14**.

m/z value	Molecular formula	RDB	Error (ppm)
121.02780	$C_7H_6O_2$	5	-14.069
139.00217	$C_6H_4O_4$	5	-10.877
165.05452	$C_9H_{10}O_3$	5	-7.255
233.08168	$C_{13}H_{14}O_4$	7	-1.082
277.07226	$C_{14}H_{14}O_6$	8	1.800
347.11415	$C_{18}H_{20}O_7$	9	1.509

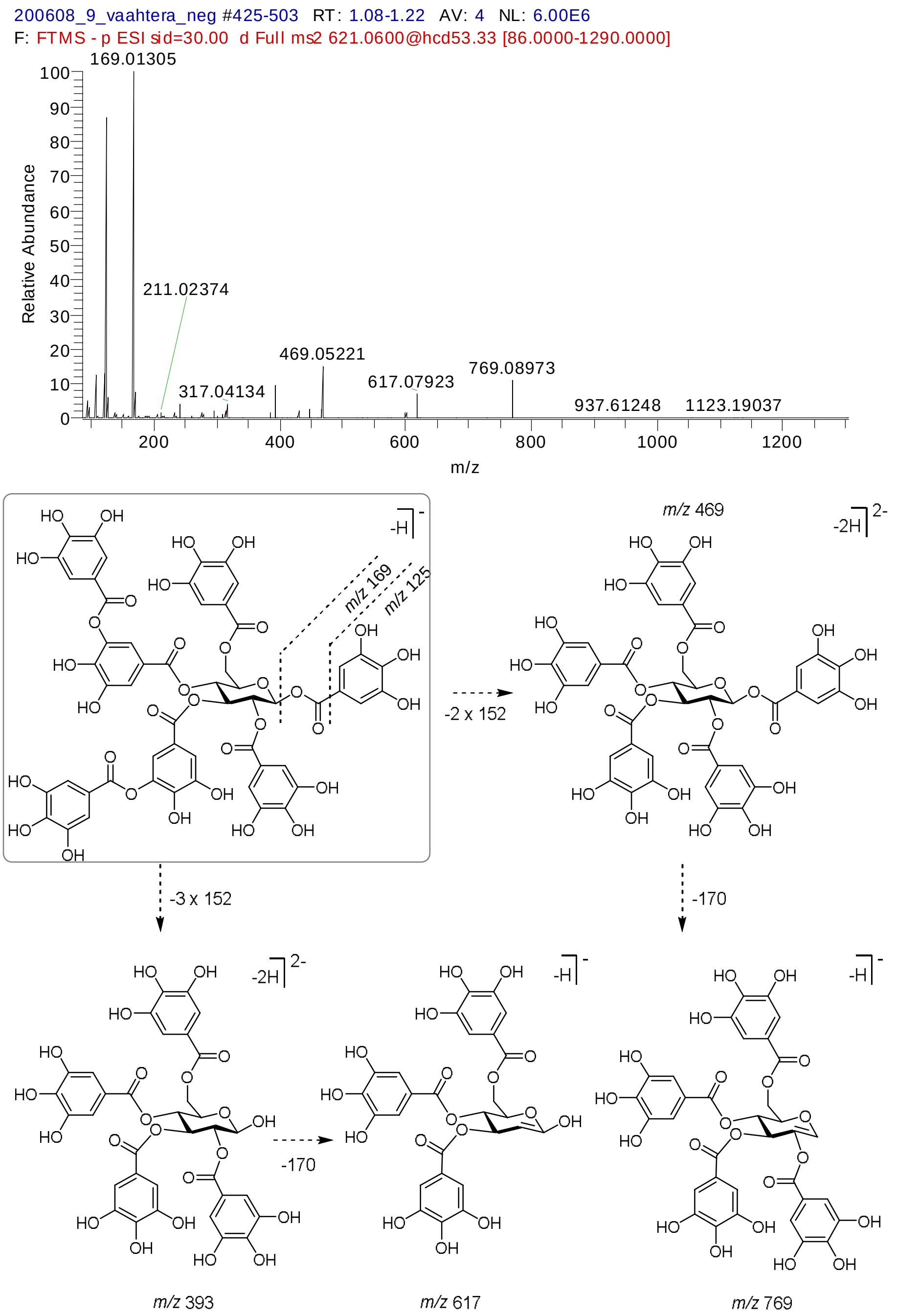

Figure S71. MS/MS spectrum and proposed fragmentation pattern of compound **15**.

Table S12. *m/z* values, molecular formulae, rings and double bond equivalents (RDB) and mass errors for the fragments of compound **15**.

m/z value	Molecular formula	RDB	Error (ppm)
125.02294	$C_6H_6O_3$	4	-11.817
169.01305	$C_7H_6O_5$	5	-7.080
393.54843	$C_{34}H_{28}O_{22}$	21	-4.617
469.55395	$C_{41}H_{32}O_{26}$	26	-3.783
617.07923	$C_{27}H_{22}O_{17}$	17	1.309
769.08973	$C_{34}H_{26}O_{21}$	22	0.454

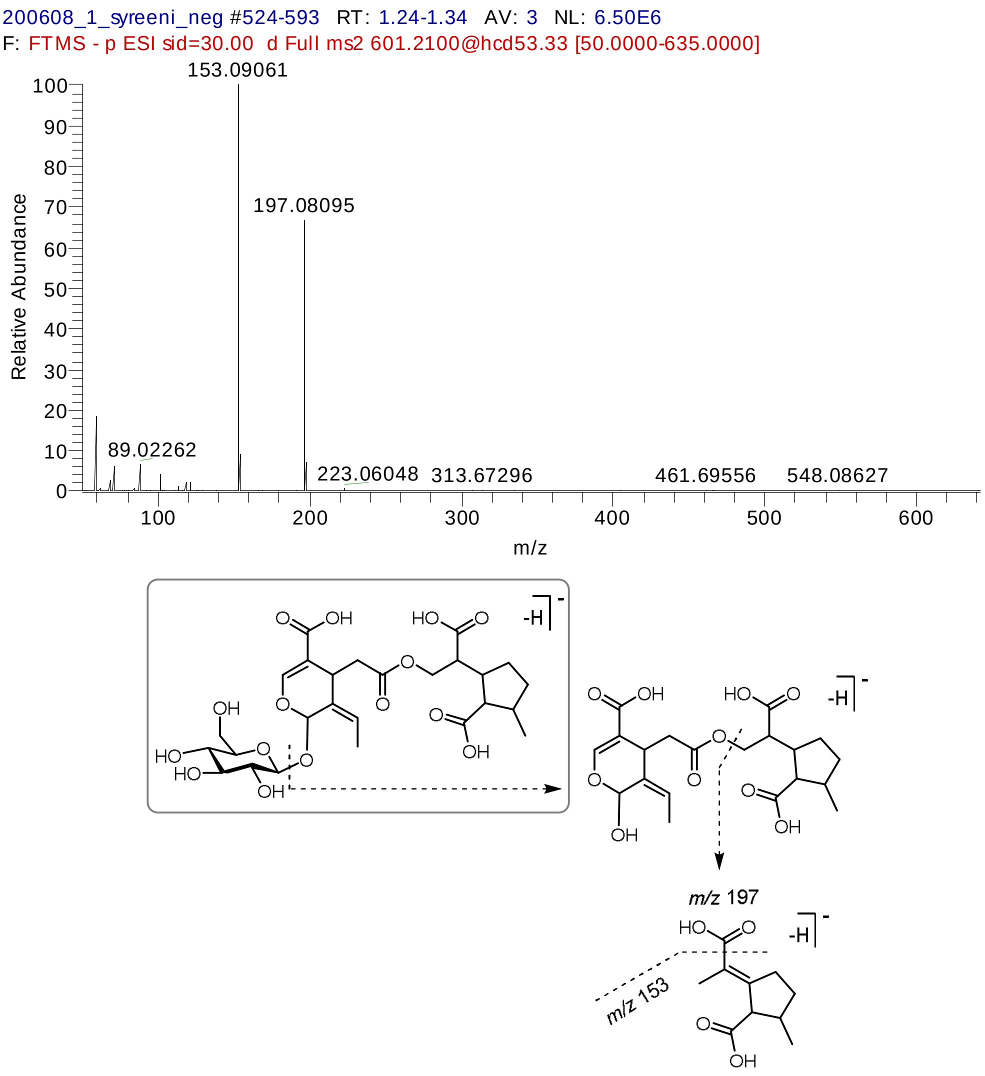

Figure S72. MS/MS spectrum and proposed fragmentation pattern of compound **16**.

Table S13. *m/z* values, molecular formulae, rings and double bond equivalents (RDB) and mass errors for the fragments of compound **16**.

m/z value	Molecular formula	RDB	Error (ppm)
153.09061	$C_9H_{14}O_2$	3	-9.752
197.08095	$C_{10}H_{14}O_4$	4	-4.984

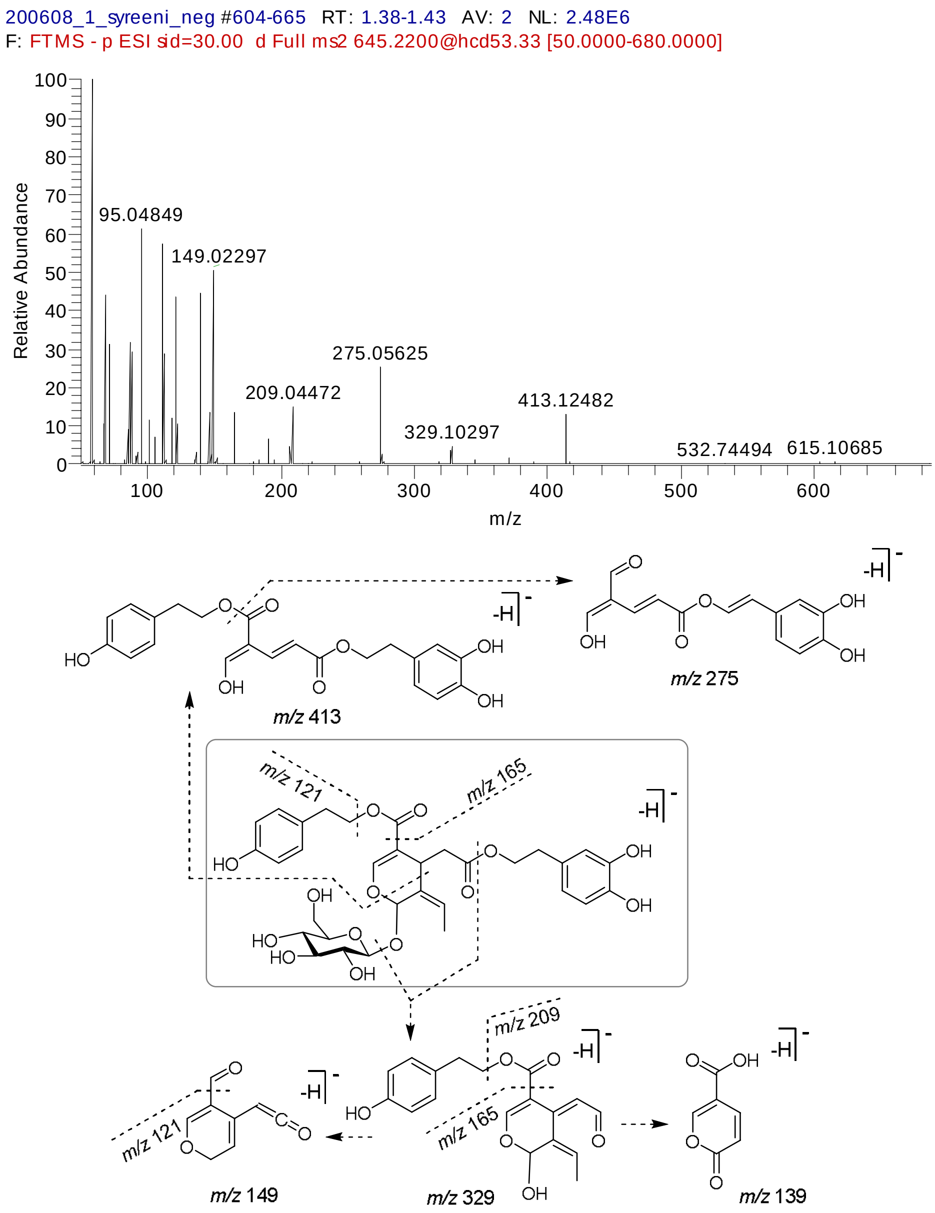

Figure S73. MS/MS spectrum and proposed fragmentation pattern of compound **17**.

Table S14. *m/z* values, molecular formulae, rings and double bond equivalents (RDB) and mass errors for the fragments of compound **17**.

m/z value	Molecular formula	RDB	Error (ppm)
121.02791	$C_7H_6O_2$	5	-13.160
121.06430	$C_8H_{10}O$	4	-13.119
139.00218	$C_6H_4O_4$	5	-10.805
149.02297	$C_8H_6O_3$	6	-9.712
165.05441	$C_9H_{10}O_3$	5	-7.921
209.04472	$C_{10}H_{10}O_5$	6	-3.955
275.05625	$C_{14}H_{12}O_6$	9	0.504
329.10297	$C_{18}H_{18}O_6$	10	-0.278
413.12482	$C_{22}H_{22}O_8$	12	1.523

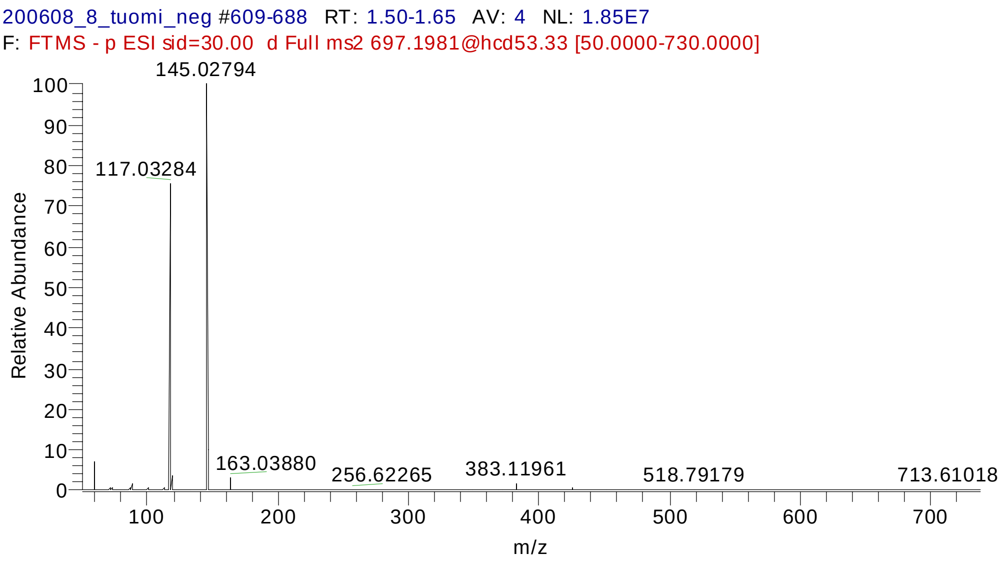

Figure S74. MS/MS spectrum of compound **18**.

Table S15. *m/z* values, molecular formulae, rings and double bond equivalents (RDB) and mass errors for the fragments of compound **18**.

m/z value	Molecular formula	RDB	Error (ppm)
117.03284	C_8H_6O	6	-14.937
145.02794	$C_9H_6O_2$	7	-10.776
163.03880	$C_9H_8O_3$	6	-7.774

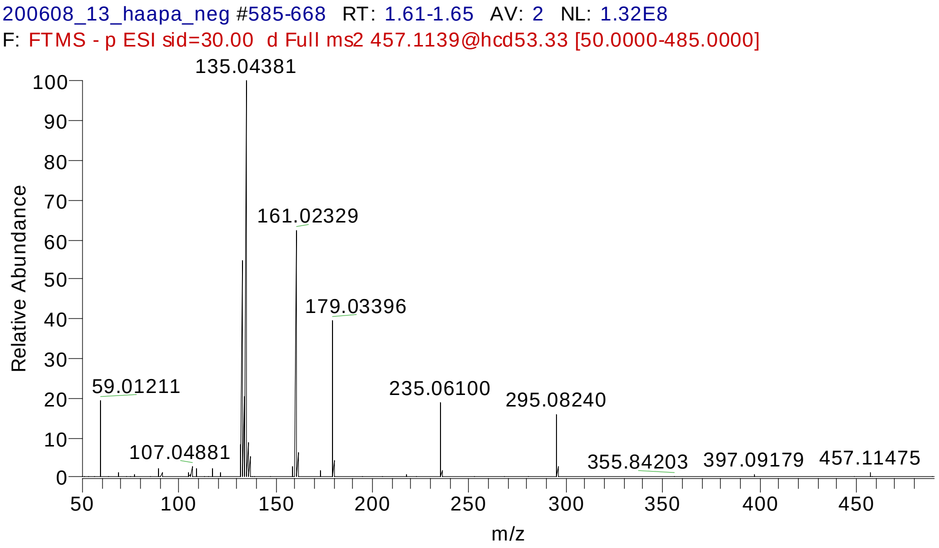

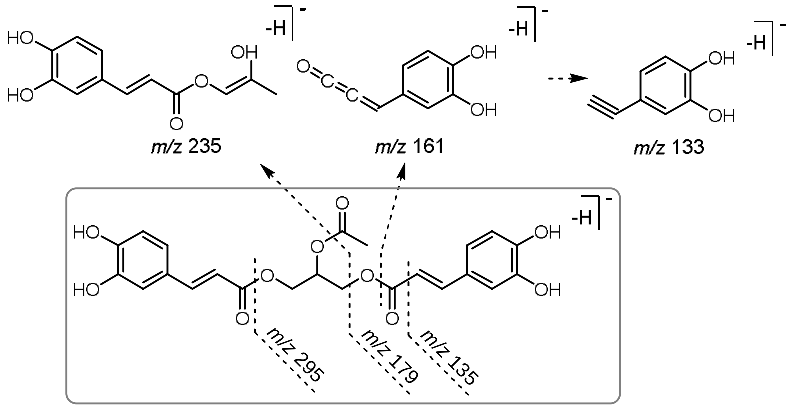

Figure S75. MS/MS spectrum and proposed fragmentation pattern of compound **19.**

Table S16. *m/z* values, molecular formulae, rings and double bond equivalents (RDB) and mass errors for the fragments of compound **19**.

m/z value	Molecular formula	RDB	Error (ppm)
133.02817	$C_8H_6O_2$	6	-10.019
135.04381	$C_8H_8O_2$	5	-9.944
161.02329	$C_9H_6O_3$	7	-7.001
179.03396	$C_9H_8O_4$	6	-5.708
235.06100	$C_{12}H_{12}O_5$	7	-0.837
295.08240	$C_{14}H_{16}O_7$	7	0.251
397.09179	$C_{21}H_{18}O_8$	13	-2.772

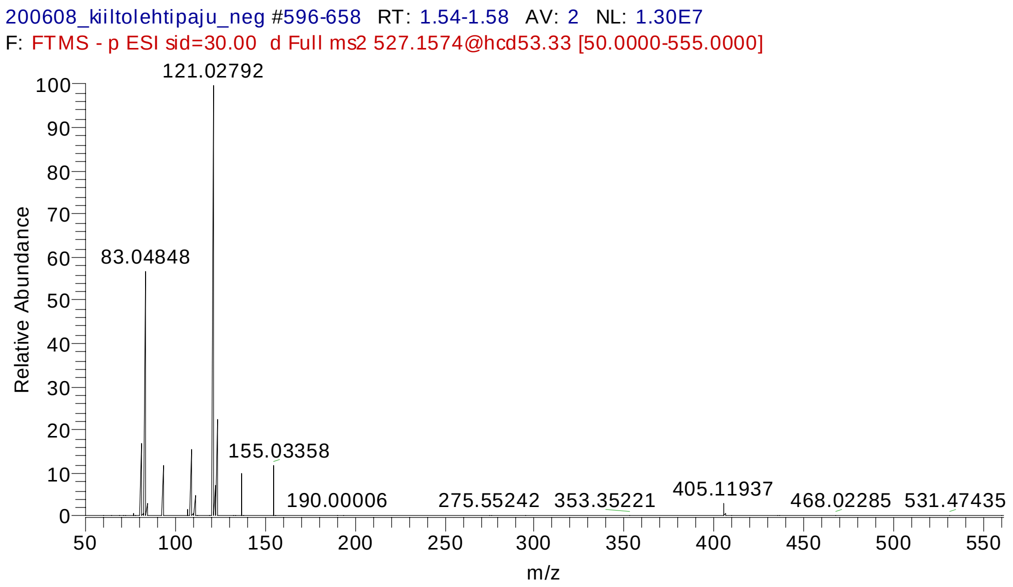

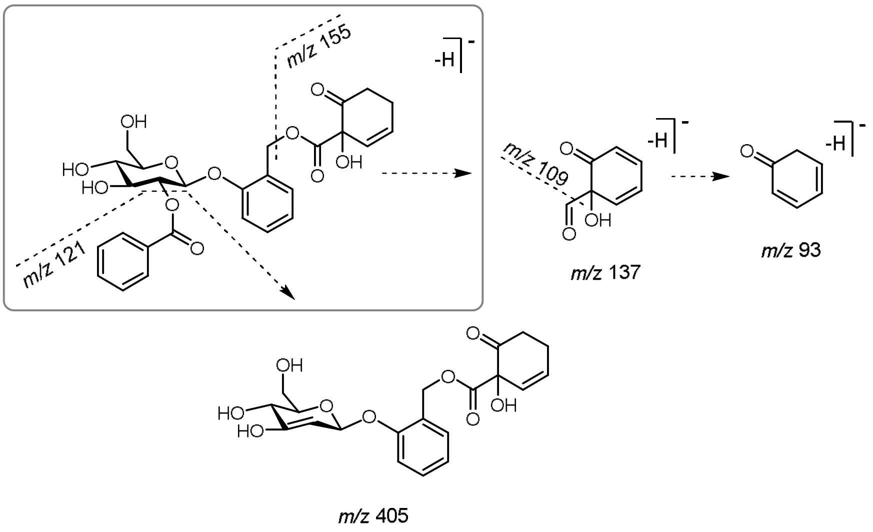

Figure S76. MS/MS spectrum and proposed fragmentation pattern of compound **20**.

Table S17. *m/z* values, molecular formulae, rings and double bond equivalents (RDB) and mass errors for the fragments of compound **20**.

m/z value	Molecular formula	RDB	Error (ppm)
93.03293	C_6H_6O	4	-17.823
109.02791	$C_6H_6O_2$	4	-14.609
121.02792	$C_7H_6O_2$	5	-13.078
137.02284	$C_7H_6O_3$	5	-11.512
155.03358	$C_7H_8O_4$	4	-0.305
405.11937	$C_{20}H_{22}O_9$	10	0.653

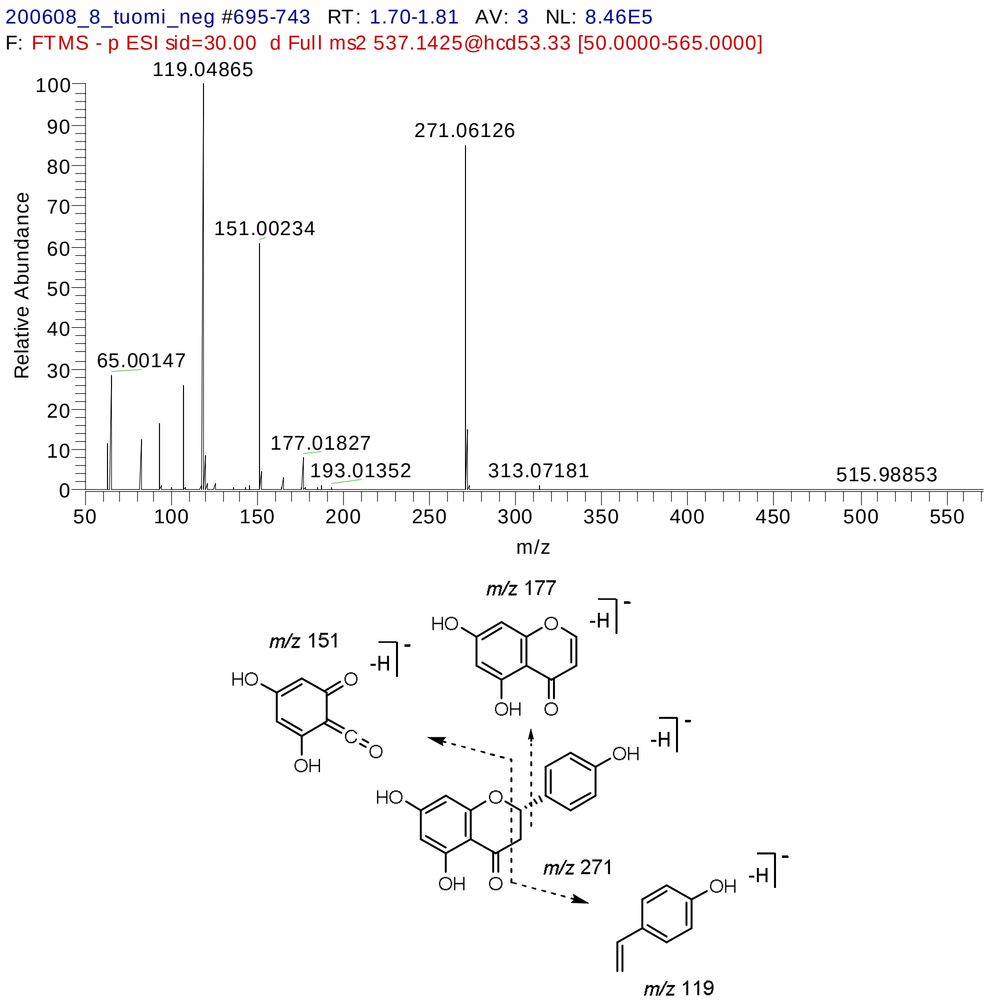

Figure S77. MS/MS spectrum and proposed fragmentation pattern of compound **21**.

Table S18. *m/z* values, molecular formulae, rings and double bond equivalents (RDB) and mass errors for the fragments of compound **21**.

m/z value	Molecular formula	RDB	Error (ppm)
119.04865	C_8H_8O	5	-13.341
151.00234	$C_7H_4O_4$	6	-8.887
177.01827	$C_9H_6O_4$	7	-5.999
271.06126	$C_{15}H_{12}O_5$	10	0.233

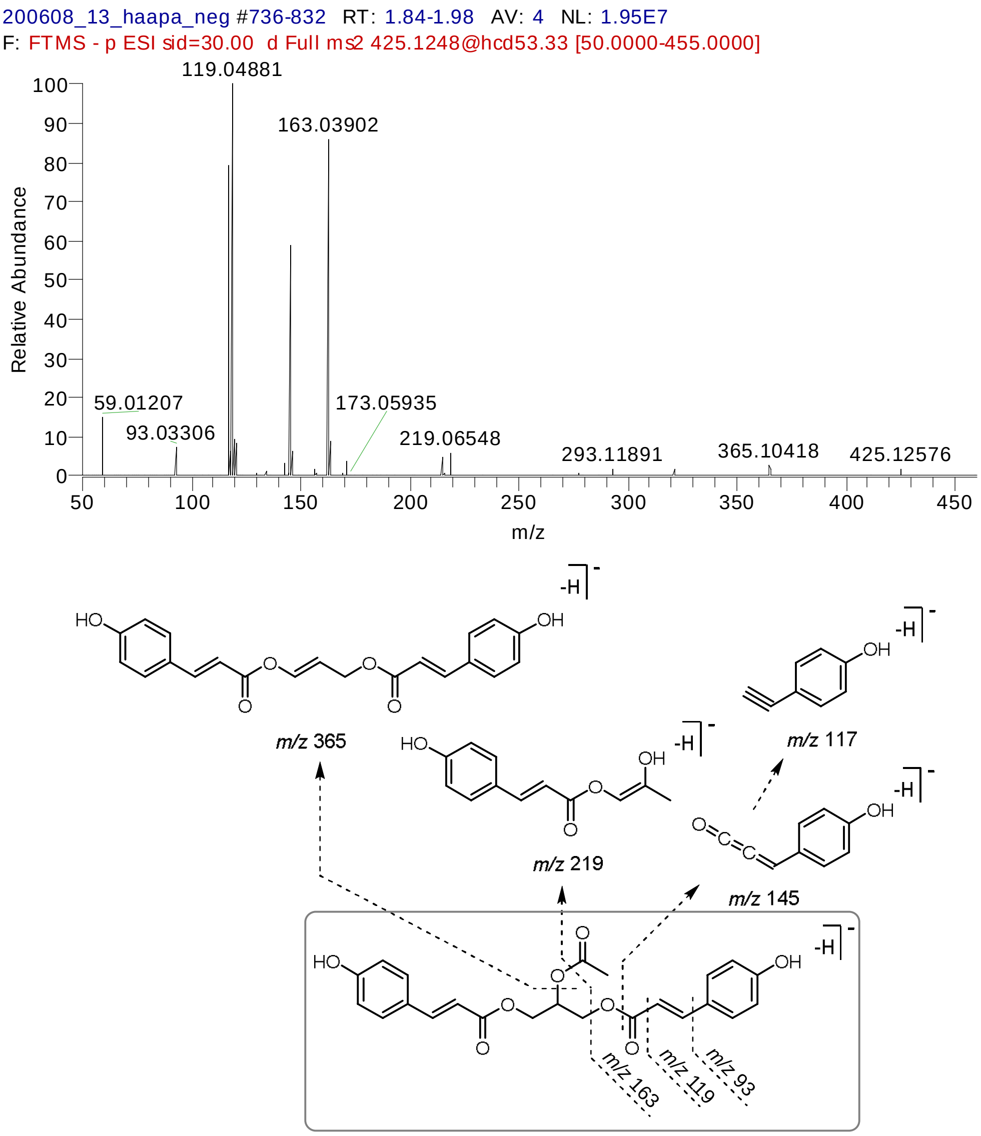

Figure S78. MS/MS spectrum and proposed fragmentation pattern of compound **22**.

Table S19. *m/z* values, molecular formulae, rings and double bond equivalents (RDB) and mass errors for the fragments of compound **22**.

m/z value	Molecular formula	RDB	Error (ppm)
93.03306	C_6H_6O	4	-16.426
117.03312	C_8H_6O	6	-12.545
119.04881	C_8H_8O	5	-11.997
145.02819	$C_9H_6O_2$	7	-9.052
163.03902	$C_9H_8O_3$	6	-6.424
219.06548	$C_{12}H_{12}O_4$	7	-3.662
365.10418	$C_{21}H_{18}O_6$	13	3.063

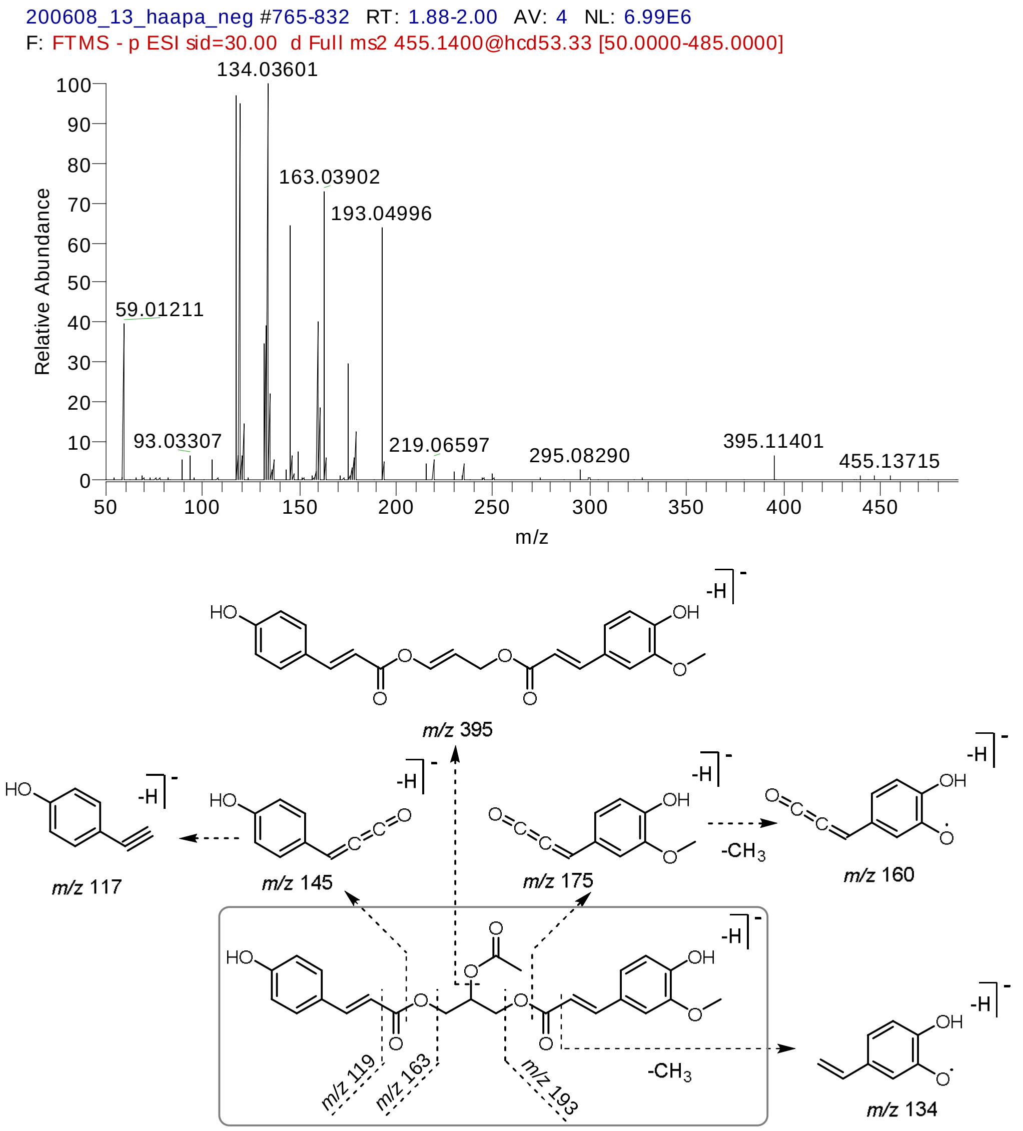

Figure S79. MS/MS spectrum and proposed fragmentation pattern of compound **23**.

Table S20. *m/z* values, molecular formulae, rings and double bond equivalents (RDB) and mass errors for the fragments of compound **23**.

m/z value	Molecular formula	RDB	Error (ppm)
117.03310	C_8H_6O	6	-12.716
119.04878	C_8H_8O	5	-12.249
134.03601	$C_8H_6O_2$	6	-9.832
145.02821	$C_9H_6O_2$	7	-8.914
160.01554	$C_9H_4O_3$	8	-6.576
163.03902	$C_9H_8O_3$	6	-6.424
175.03918	$C_{10}H_8O_3$	7	-5.070
193.04996	$C_{10}H_{10}O_4$	6	-3.481
395.11401	$C_{22}H_{20}O_7$	13	0.971

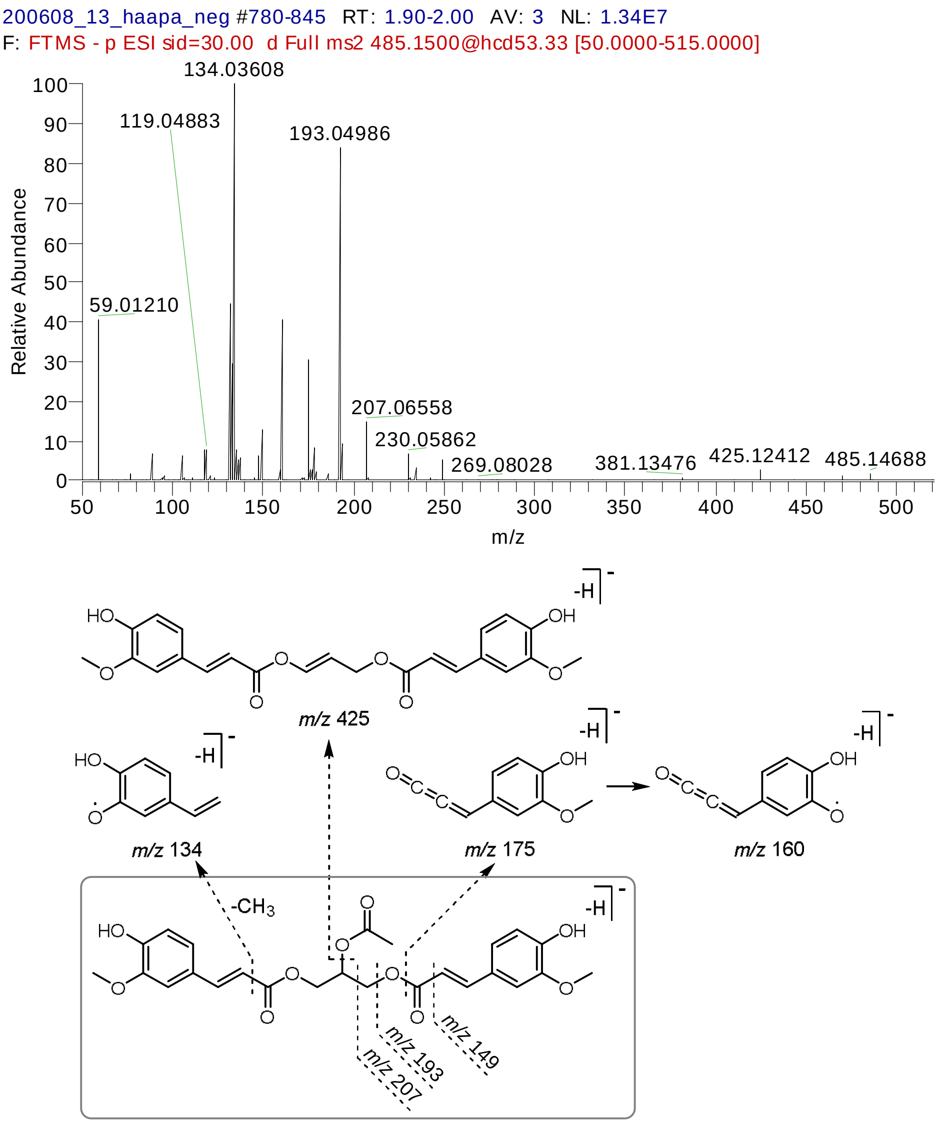

Figure S80. MS/MS spectrum and proposed fragmentation pattern of compound **24**.

Table S21. *m/z* values, molecular formulae, rings and double bond equivalents (RDB) and mass errors for the fragments of compound **24**.

m/z value	Molecular formula	RDB	Error (ppm)
134.03608	$C_8H_6O_2$	6	-9.309
149.05951	$C_9H_{10}O_2$	5	-8.674
160.01544	$C_9H_4O_3$	8	-7.201
175.03939	$C_{10}H_8O_3$	7	-3.870
193.04986	$C_{10}H_{10}O_4$	6	-3.999
207.06558	$C_{11}H_{12}O_4$	6	-3.391
425.12412	$C_{23}H_{22}O_8$	13	-0.167

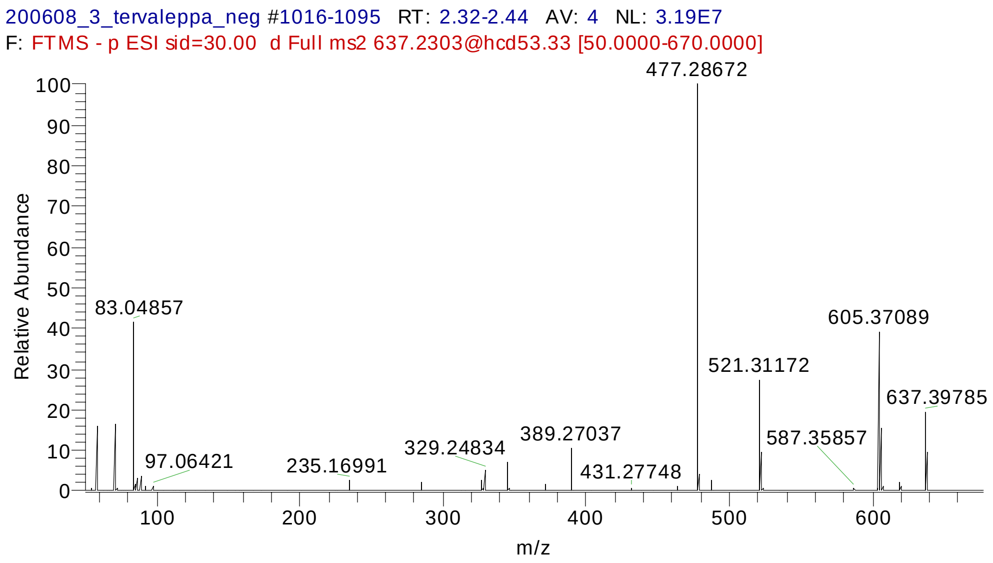

Figure S81. MS/MS spectrum of compounds **25** and **26**.

Table S22. *m/z* values, molecular formulae, rings and double bond equivalents (RDB) and mass errors for the fragments of compounds **25** and **26**.

m/z value	Molecular formula	RDB	Error (ppm)
389.27037	$C_{24}H_{38}O_4$	6	1.637
477.28672	$C_{27}H_{42}O_7$	7	1.976
521.31172	$C_{29}H_{46}O_8$	7	-0.521
605.37089	$C_{34}H_{54}O_9$	8	2.286

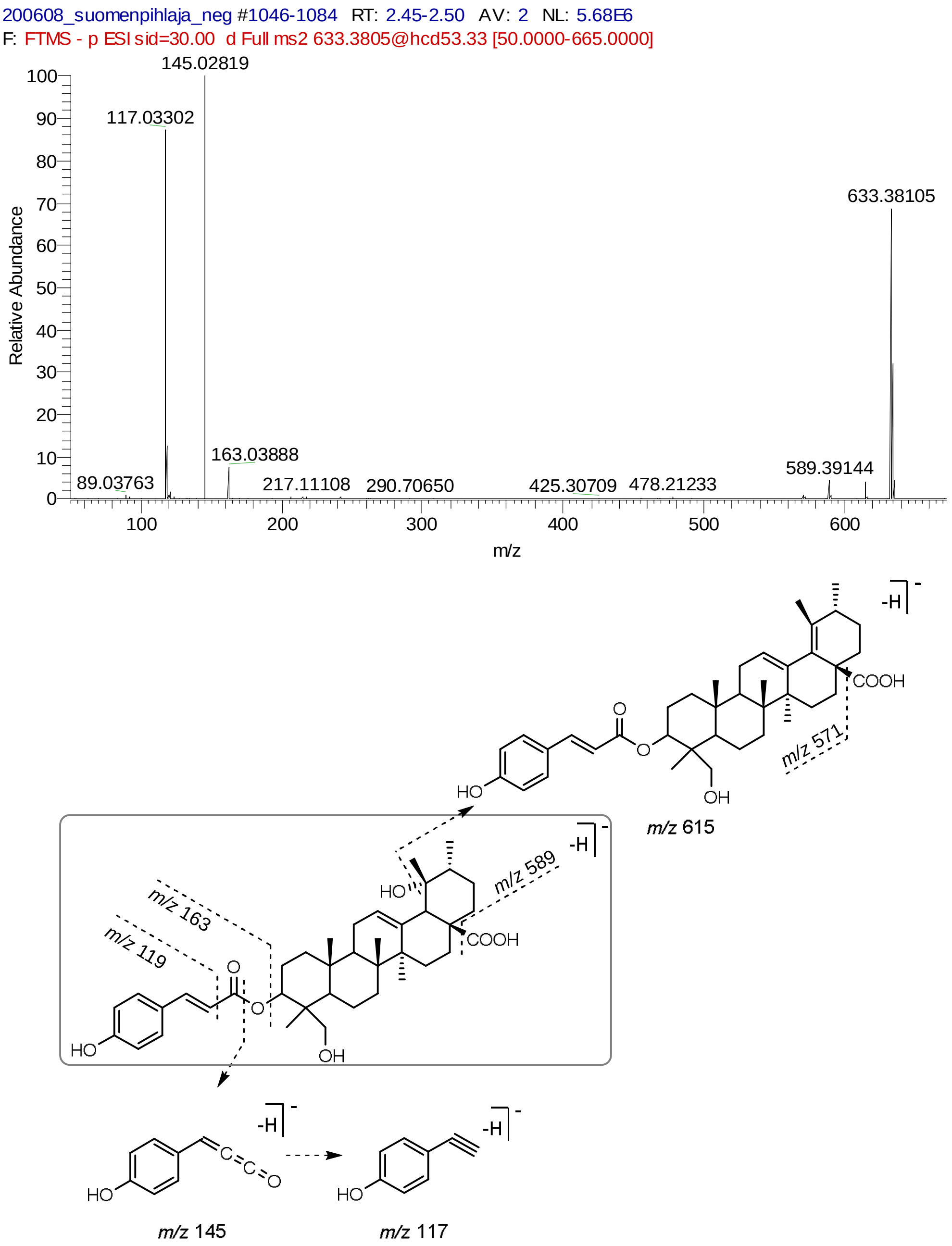

Figure S82. MS/MS spectrum and proposed fragmentation pattern of compound **27**.

Table S23. *m/z* values, molecular formulae, rings and double bond equivalents (RDB) and mass errors for the fragments of compound **27**.

m/z value	Molecular formula	RDB	Error (ppm)
117.03302	C_8H_6O	6	-13.399
119.04860	C_8H_8O	5	-13.761
145.02819	$C_9H_6O_2$	7	-9.052
163.03888	$C_9H_8O_3$	6	-7.283
571.37692	$C_{38}H_{52}O_4$	13	-4.136
589.39144	$C_{38}H_{54}O_5$	12	2.701
615.36883	$C_{39}H_{52}O_6$	14	-0.459

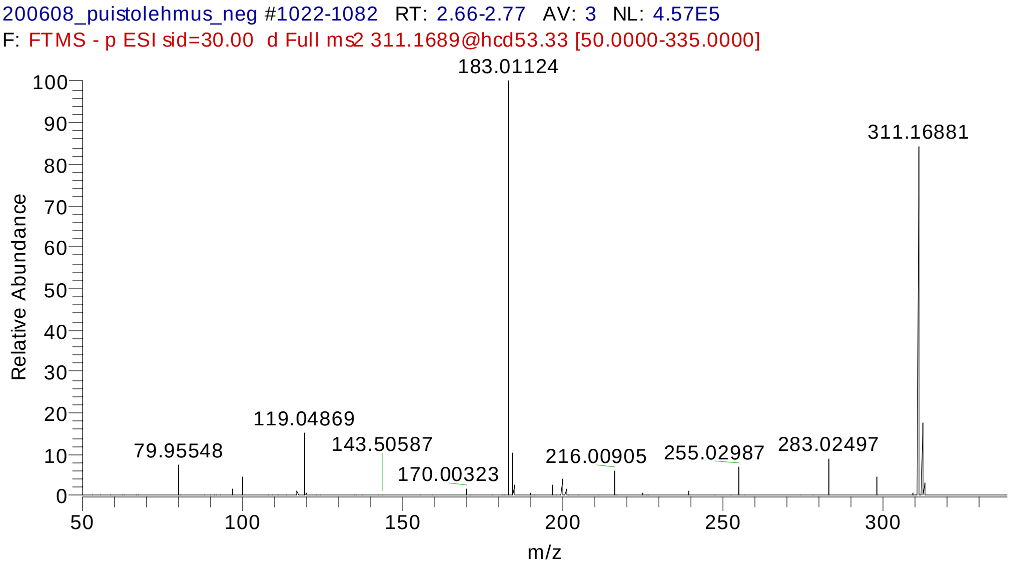

Figure S83. MS/MS spectrum of compound **28**.

Table S24. *m/z* values, molecular formulae, rings and double bond equivalents (RDB) and mass errors for the fragments of compound **28**.

m/z value	Molecular formula	RDB	Error (ppm)
119.04869	C_8H_8O	5	-13.005
183.01124	$C_{11}H_4O_3$	10	13.511

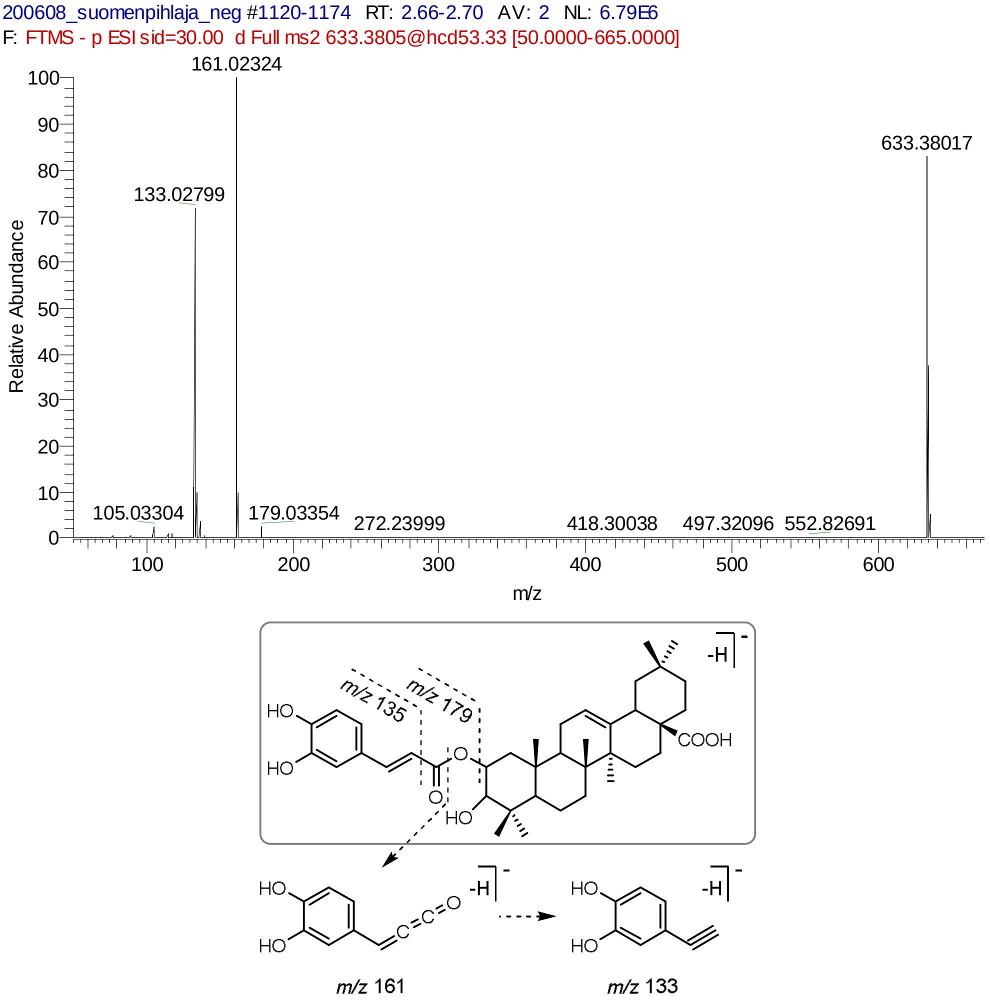

Figure S84. MS/MS spectrum and proposed fragmentation pattern of compound **29**.

Table S25. *m/z* values, molecular formulae, rings and double bond equivalents (RDB) and mass errors for the fragments of compound **29**.

m/z value	Molecular formula	RDB	Error (ppm)
133.02799	$C_8H_6O_2$	6	-11.372
135.04377	$C_8H_8O_2$	5	-10.240
161.02324	$C_9H_6O_3$	7	-7.312
179.03354	$C_9H_8O_4$	6	-8.054

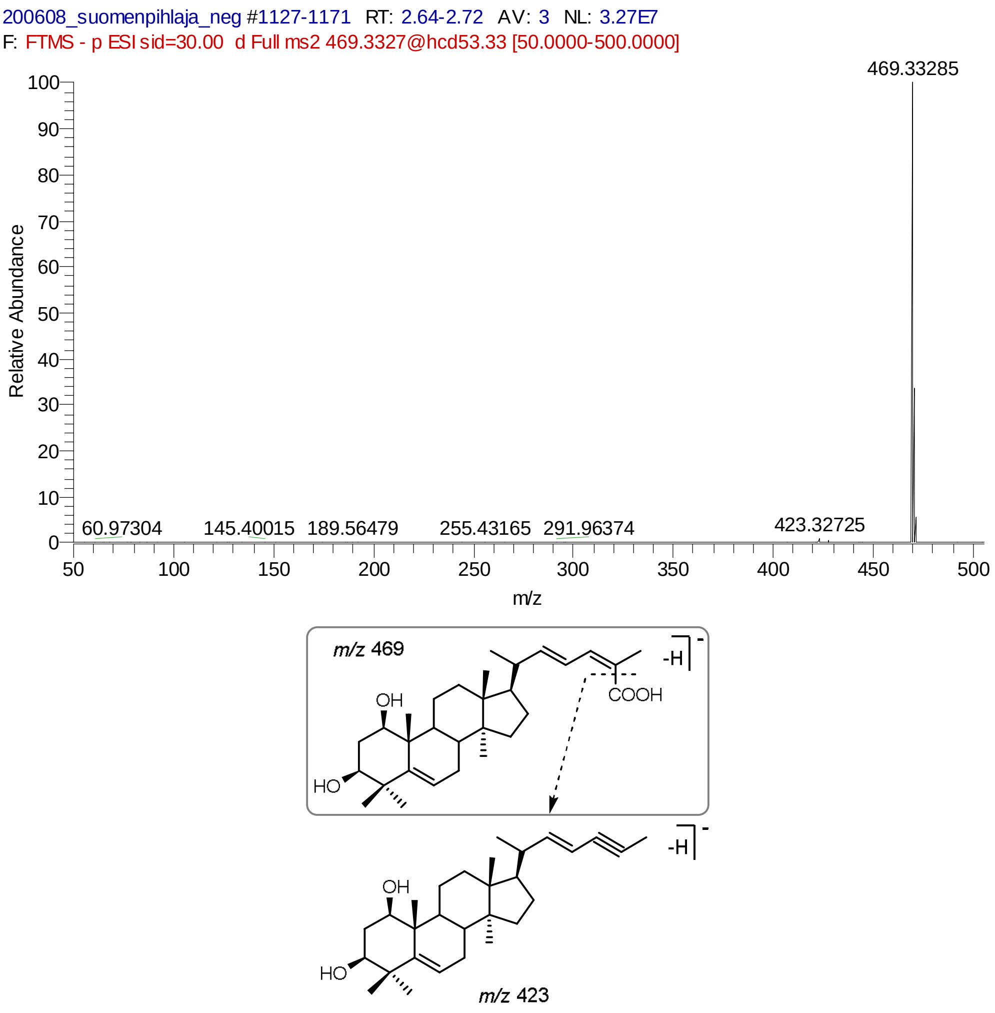

Figure S85. MS/MS spectrum and proposed fragmentation pattern of compound **30**.

Table S26. *m/z* values, molecular formulae, rings and double bond equivalents (RDB) and mass errors for the fragment of compound **30**.

m/z value	Molecular formula	RDB	Error (ppm)
423.32725	$C_{29}H_{44}O_2$	8	0.936

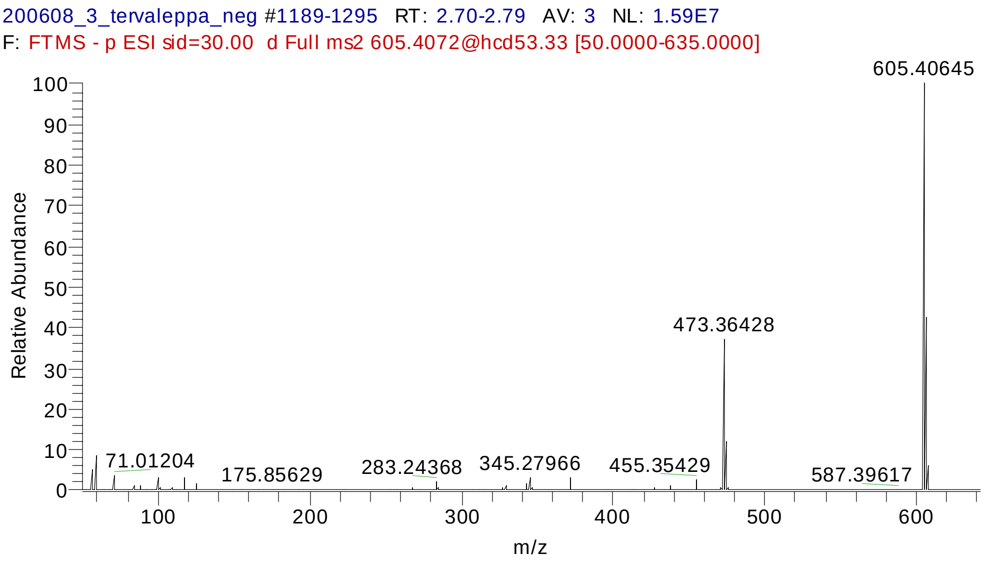

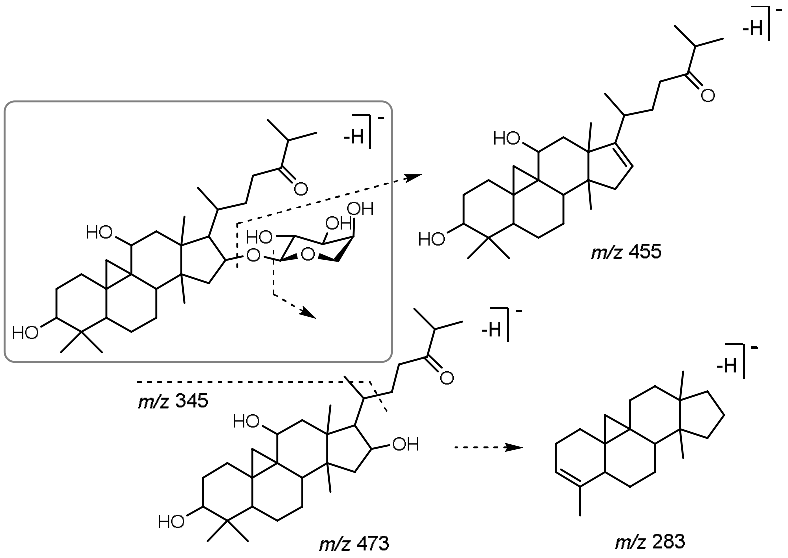

Figure S86. MS/MS spectrum of isomeric compounds **31** and **33** and the proposed fragmentation pattern of one of the isomers.

Table S27. *m/z* values, molecular formulae, rings and double bond equivalents (RDB) and mass errors for the fragments of compounds **31** and **33**.

m/z value	Molecular formula	RDB	Error (ppm)
473.36428	$C_{30}H_{50}O_4$	6	1.366
455.35429	$C_{30}H_{48}O_3$	7	2.682
345.27966	$C_{23}H_{38}O_2$	5	-0.706
283.24368	$C_{21}H_{32}$	6	1.962

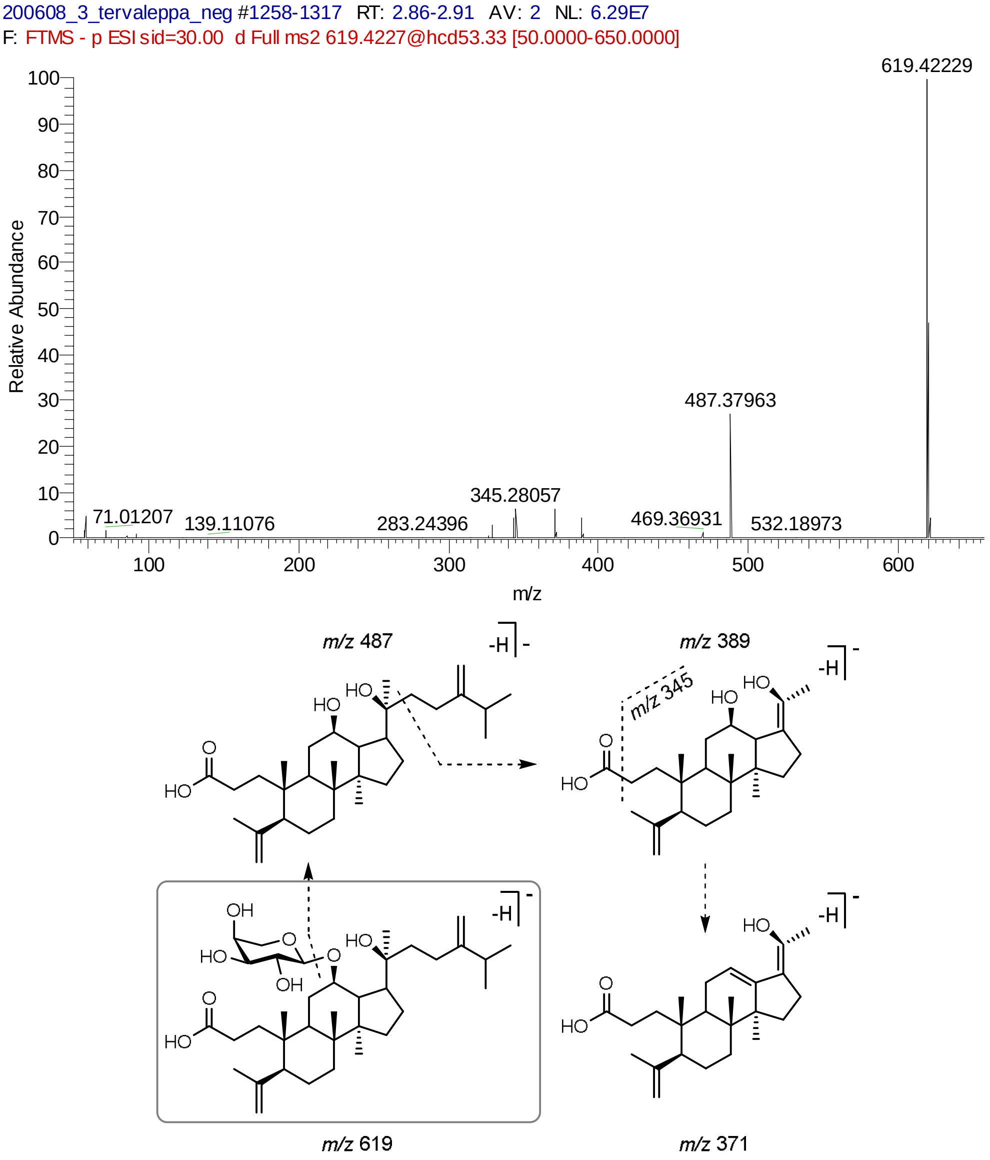

Figure S87. MS/MS spectrum of isomeric compounds **32** and **34** and the proposed fragmentation pattern of one of the isomers.

Table S28. *m/z* values, molecular formulae, rings and double bond equivalents (RDB) and mass errors for the fragments of compounds **32** and **34**.

m/z value	Molecular formula	RDB	Error (ppm)
343.26598	$C_{23}H_{36}O_2$	6	5.029
345.28057	$C_{23}H_{38}O_2$	5	1.930
371.25960	$C_{24}H_{36}O_3$	7	1.163
389.27126	$C_{24}H_{38}O_4$	6	3.923
487.37963	$C_{31}H_{52}O_4$	6	0.711

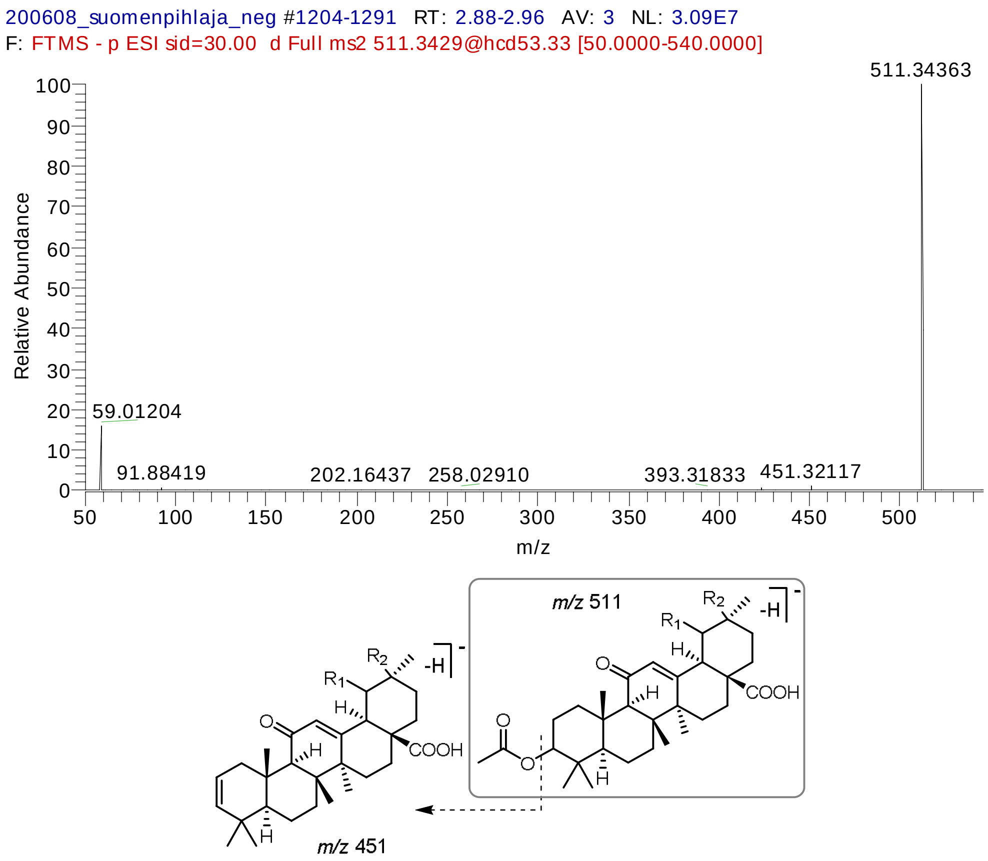

Figure S88. MS/MS spectrum and proposed fragmentation pattern of compound **35**. R_1 = H and R_2 = CH_3 or vice versa.

Table S29. *m/z* values, molecular formulae, rings and double bond equivalents (RDB) and mass errors for the fragment of compound **35**.

m/z value	Molecular formula	RDB	Error (ppm)
451.32117	$C_{30}H_{44}O_3$	9	-1.326

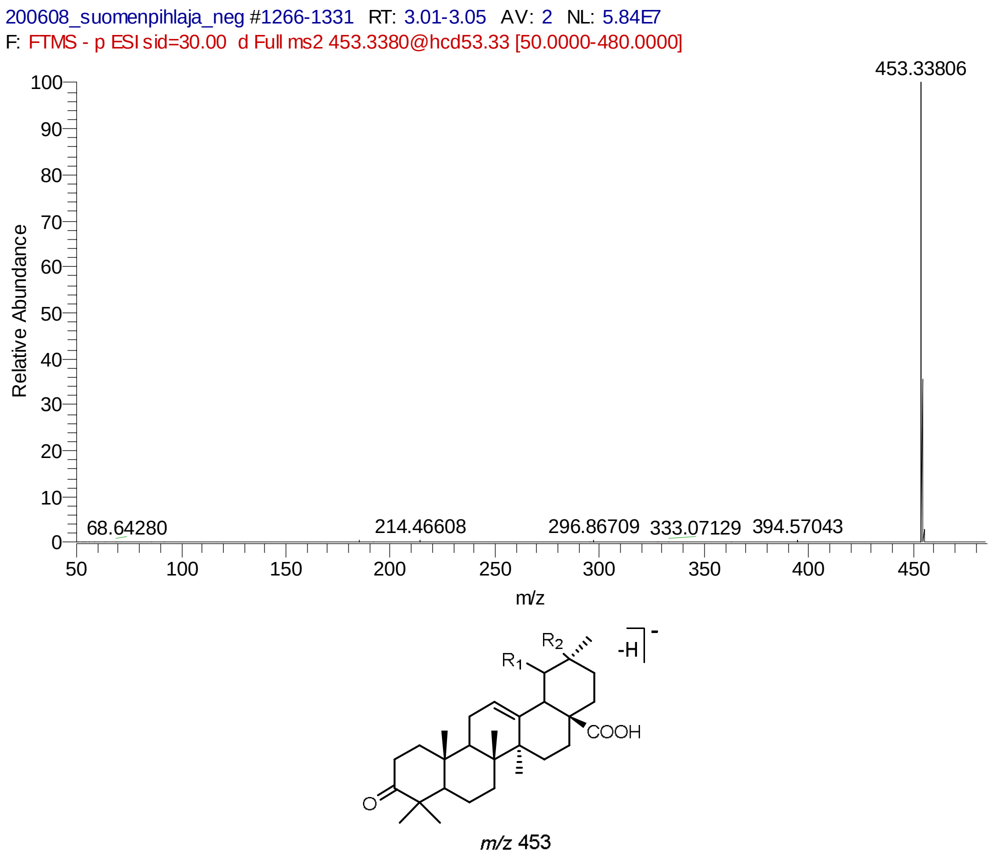

Figure S89. MS/MS spectrum and proposed structure of compound **36**. R_1 = H and R_2 = CH_3 or vice versa.

Table S30. *m/z* values, molecular formulae, rings and double bond equivalents (RDB) and mass errors for the fragments of compound **36**. n/a = not available.

m/z value	Molecular formula	RDB	Error (ppm)
n/a	n/a	n/a	n/a

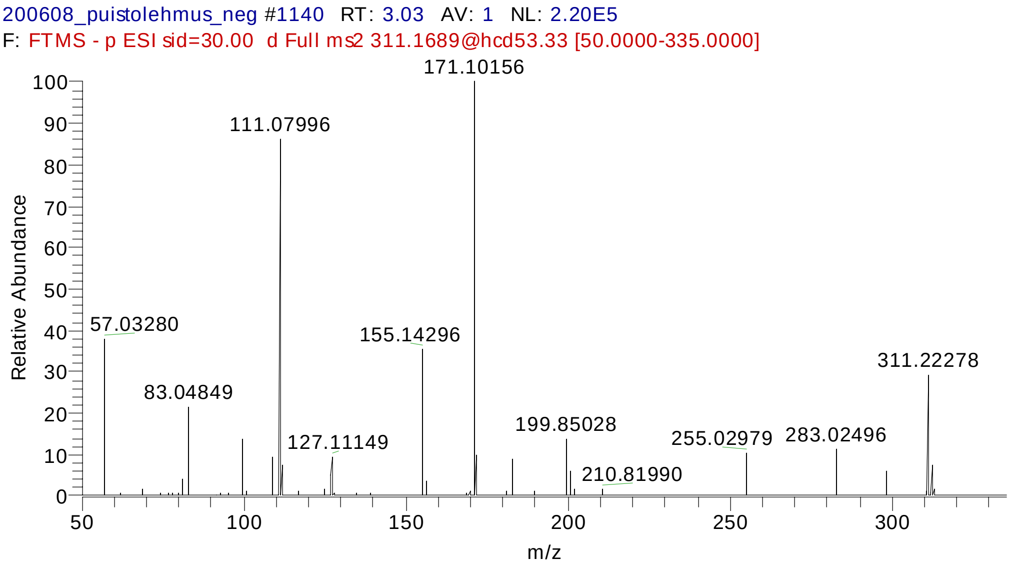

Figure S90. MS/MS spectrum of compound **37**.

Table S31. *m/z* values, molecular formulae, rings and double bond equivalents (RDB) and mass errors for the fragments of compound **37**.

m/z value	Molecular formula	RDB	Error (ppm)
111.07996	$C_7H_{12}O$	2	-14.209
155.14296	$C_{10}H_{20}O$	1	-7.597
171.10156	$C_9H_{16}O_3$	2	-6.474

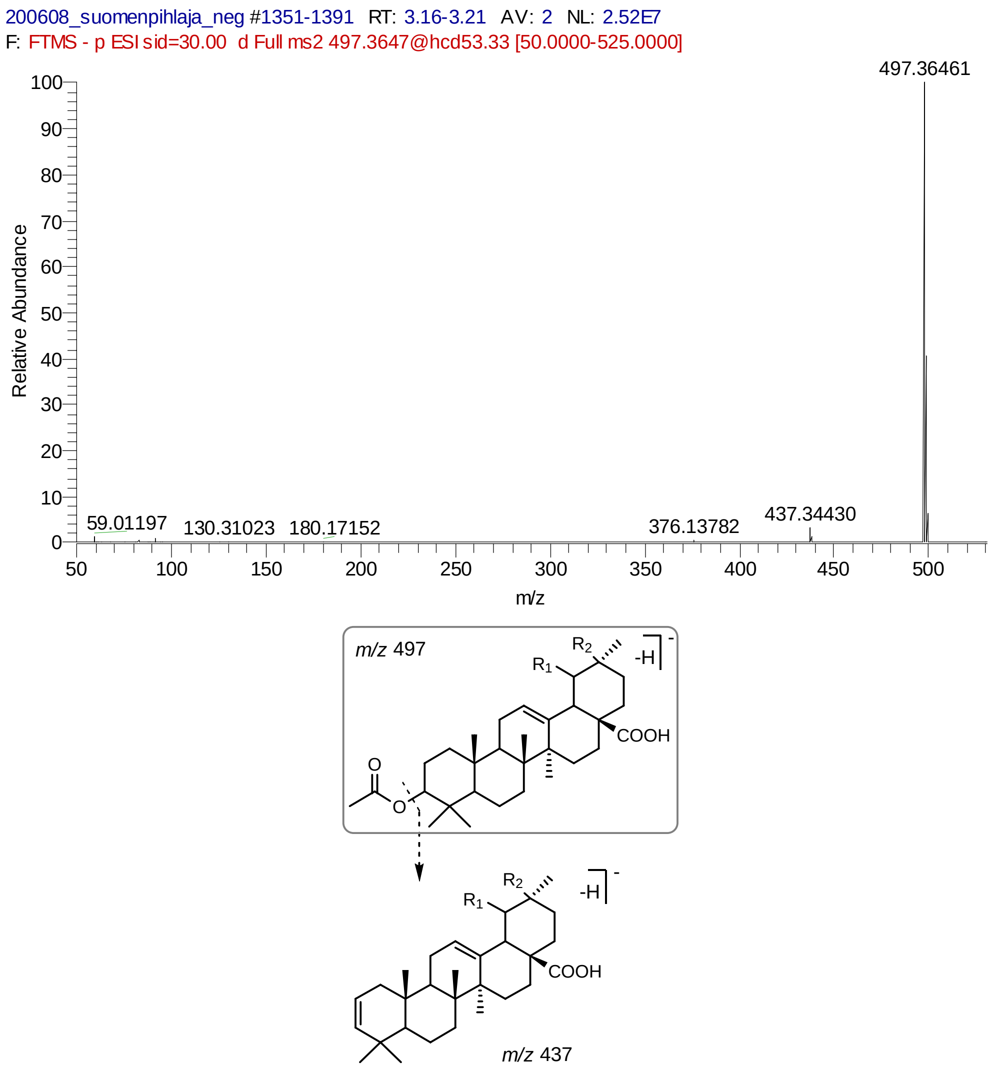

Figure S91. MS/MS spectrum and proposed fragmentation pattern of compound **38**. R_1 = H and R_2 = CH_3 or vice versa.

Table S32. *m/z* values, molecular formulae, rings and double bond equivalents (RDB) and mass errors for the fragment of compound **38**.

m/z value	Molecular formula	RDB	Error (ppm)
437.34430	$C_{30}H_{46}O_2$	8	4.107

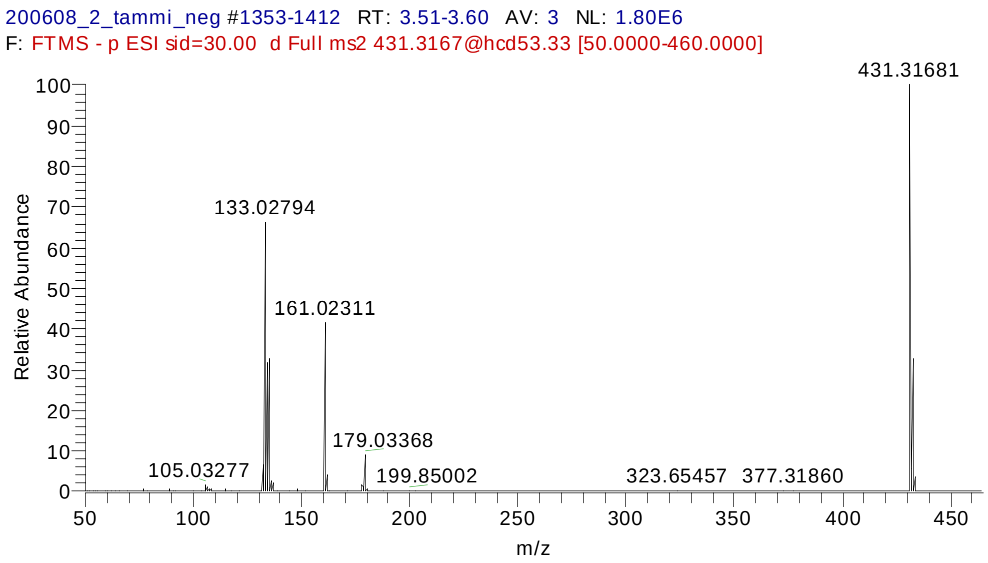

Figure S92. MS/MS spectrum of compound **39**.

Table S33. *m/z* values, molecular formulae, rings and double bond equivalents (RDB) and mass errors for the fragments of compound **39**.

m/z value	Molecular formula	RDB	Error (ppm)
133.02794	$C_8H_6O_2$	6	-11.748
161.02311	$C_9H_6O_3$	7	-8.119
179.03368	$C_9H_8O_4$	6	-7.272

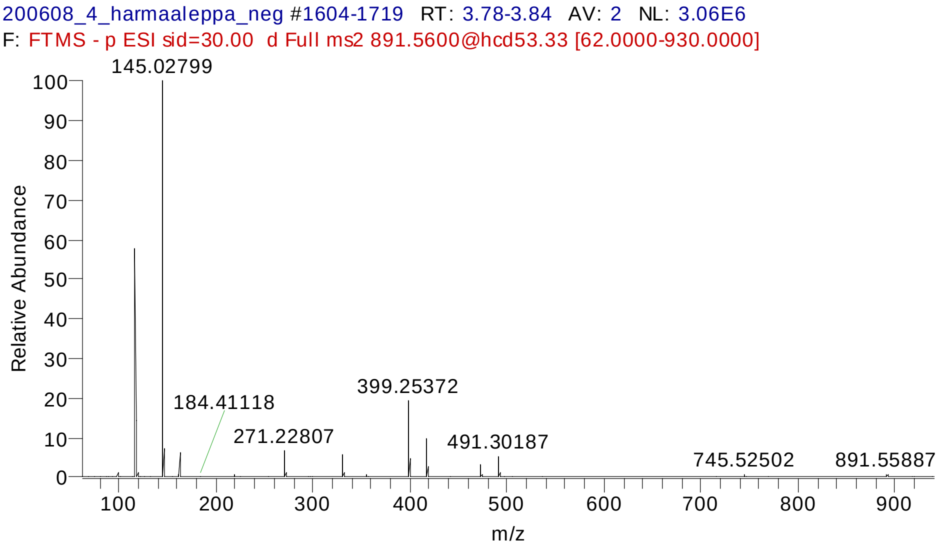

Figure S93. MS/MS spectrum of compound **40**.

Table S34. *m/z* values, molecular formulae, rings and double bond equivalents (RDB) and mass errors for the fragments of compound **40**.

m/z value	Molecular formula	RDB	Error (ppm)
117.03298	C_8H_6O	6	-13.741
145.02799	$C_9H_6O_2$	7	-10.431
163.03883	$C_9H_8O_3$	6	-7.590
271.22807	$C_{16}H_{32}O_3$	1	0.744
331.26423	$C_{22}H_{36}O_2$	5	-0.071
399.25372	$C_{25}H_{36}O_4$	8	-0.909
417.26467	$C_{25}H_{38}O_5$	7	0.054
491.30187	$C_{28}H_{44}O_7$	7	0.902
745.52502	$C_{44}H_{74}O_9$	8	-1.324

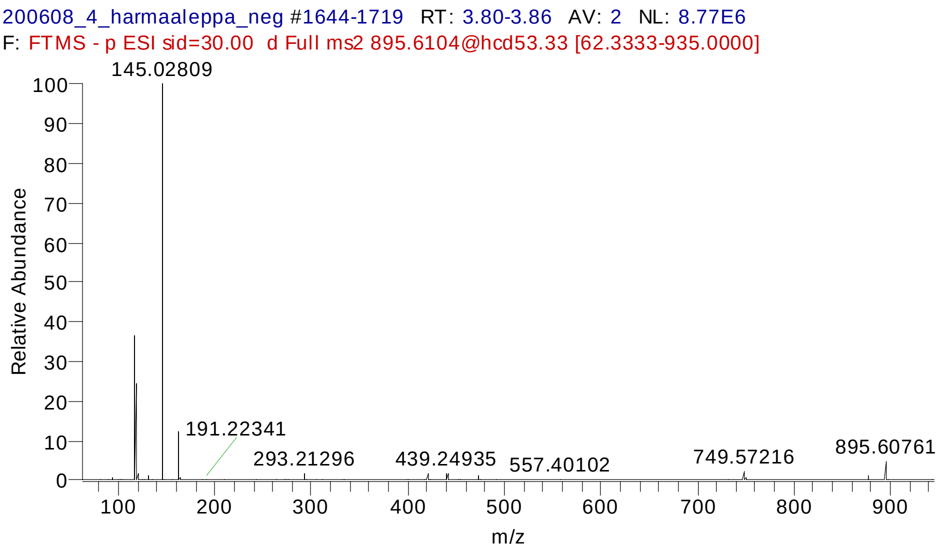

Figure S94. MS/MS spectrum of compound **41**.

Table S35. *m/z* values, molecular formulae, rings and double bond equivalents (RDB) and mass errors for the fragments of compound **41**.

m/z value	Molecular formula	RDB	Error (ppm)
117.03307	C_8H_6O	6	-12.972
119.04870	C_8H_8O	5	-12.921
145.02809	$C_9H_6O_2$	7	-9.741
163.03864	$C_9H_8O_3$	6	-8.755
749.57216	$C_{48}H_{78}O_6$	10	-0.538

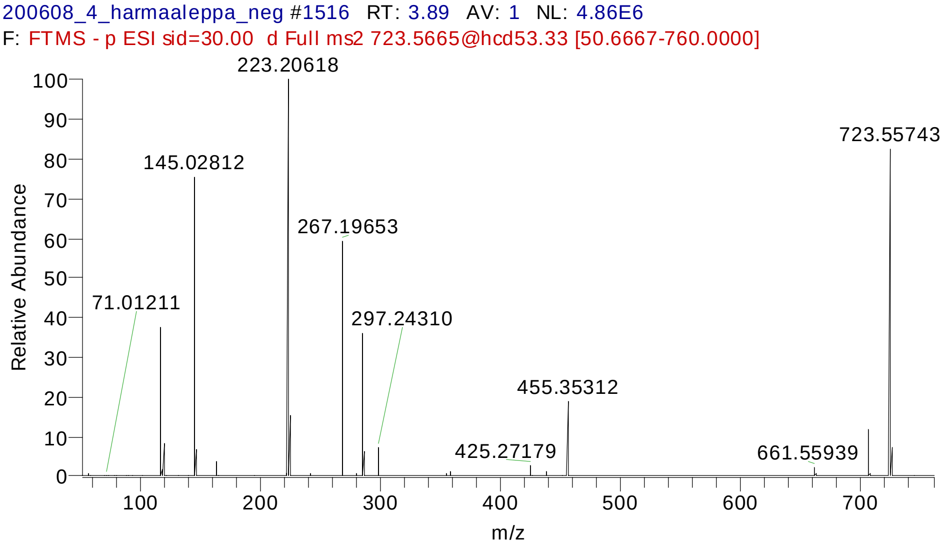

Figure S95. MS/MS spectrum of compound **42**.

Table S36. *m/z* values, molecular formulae, rings and double bond equivalents (RDB) and mass errors for the fragments of compound **42**.

m/z value	Molecular formula	RDB	Error (ppm)
117.03300	C_8H_6O	6	-13.570
145.02812	$C_9H_6O_2$	7	-9.534
223.20618	$C_{15}H_{28}O$	2	-2.504
267.19653	$C_{16}H_{28}O_3$	3	-0.142
285.20718	$C_{16}H_{30}O_4$	2	0.166
297.24310	$C_{18}H_{34}O_3$	2	-1.407
455.35312	$C_{30}H_{48}O_3$	7	0.113
705.54700	$C_{46}H_{74}O_5$	10	0.923
